# High‐throughput proteomics of breast cancer interstitial fluid: identification of tumor subtype‐specific serologically relevant biomarkers

**DOI:** 10.1002/1878-0261.12850

**Published:** 2021-01-04

**Authors:** Thilde Terkelsen, Maria Pernemalm, Pavel Gromov, Anna‐Lise Børresen‐Dale, Anders Krogh, Vilde D. Haakensen, Janne Lethiö, Elena Papaleo, Irina Gromova

**Affiliations:** ^1^ Computational Biology Laboratory Danish Cancer Society Research Center Copenhagen Denmark; ^2^ Cancer Proteomics Mass Spectrometry Science for Life Laboratory Department of Oncology‐Pathology Karolinska Institutet Stockholm Sweden; ^3^ Breast Cancer Biology Group Genome Integrity Unit Danish Cancer Society Research Center Copenhagen Denmark; ^4^ Department of Cancer Genetics Institute for Cancer Research The Norwegian Radium Hospital Oslo University Hospital Norway; ^5^ Department of Computer Science University of Copenhagen Denmark; ^6^ Department of Biology University of Copenhagen Denmark; ^7^ Translational Disease System Biology Faculty of Health and Medical Sciences Novo Nordisk Foundation Center for Protein Research University of Copenhagen Denmark

**Keywords:** breast cancer, interstitial fluid, proteome, serological markers, subtype, tumor infiltrating lymphocytes

## Abstract

Despite significant advancements in breast cancer (BC) research, clinicians lack robust serological protein markers for accurate diagnostics and tumor stratification. Tumor interstitial fluid (TIF) accumulates aberrantly externalized proteins within the local tumor space, which can potentially gain access to the circulatory system. As such, TIF may represent a valuable starting point for identifying relevant tumor‐specific serological biomarkers. The aim of the study was to perform comprehensive proteomic profiling of TIF to identify proteins associated with BC tumor status and subtype. A liquid chromatography tandem mass spectrometry (LC‐MS/MS) analysis of 35 TIFs of three main subtypes: luminal (19), Her2 (4), and triple‐negative (TNBC) (12) resulted in the identification of > 8800 proteins. Unsupervised hierarchical clustering segregated the TIF proteome into two major clusters, luminal and TNBC/Her2 subgroups. High‐grade tumors enriched with tumor infiltrating lymphocytes (TILs) were also stratified from low‐grade tumors. A consensus analysis approach, including differential abundance analysis, selection operator regression, and random forest returned a minimal set of 24 proteins associated with BC subtypes, receptor status, and TIL scoring. Among them, a panel of 10 proteins, AGR3, BCAM, CELSR1, MIEN1, NAT1, PIP4K2B, SEC23B, THTPA, TMEM51, and ULBP2, was found to stratify the tumor subtype‐specific TIFs. In particular, upregulation of BCAM and CELSR1 differentiates luminal subtypes, while upregulation of MIEN1 differentiates Her2 subtypes. Immunohistochemistry analysis showed a direct correlation between protein abundance in TIFs and intratumor expression levels for all 10 proteins. Sensitivity and specificity were estimated for this protein panel by using an independent, comprehensive breast tumor proteome dataset. The results of this analysis strongly support our data, with eight of the proteins potentially representing biomarkers for stratification of BC subtypes. Five of the most representative proteomics databases currently available were also used to estimate the potential for these selected proteins to serve as putative serological markers.

## Graphical abstract

Analysis of breast tumor interstitial fluids (TIF) may help identify protein biomarkers that are secreted in the circulation. Following extraction of breast tumor TIF, LC‐MS/MS TMT labeled proteomics, and bioinformatic analyses we identified a panel of secreted protein biomarkers. Experimental and computational validation using tissues and external datasets, respectively, indicated the potential of this biomarker panel for breast cancer subtype stratification.

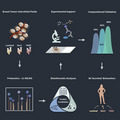

**Abbreviations**
AUC: area under the curve
BC: breast cancer
DAA: differential abundance analysis
ER: estrogen receptor
FIF: fat interstitial fluid
Her2: human epidermal growth factor receptor 2
IHC: immunohistochemistry
LASSO: least absolute shrinkage and selection operator
MPs: microparticles
NIF: normal interstitial fluid
PgR: progesterone receptor
PPI: protein‐protein interaction
RF: random forest
TIF: tumor interstitial fluid
TILs: tumor infiltrating lymphocytes
TNBC: triple‐negative breast cancer

## 1 Introduction

Breast cancer (BC) is the most prevalent form of cancer among women worldwide, with 2.1 million new cases registered in 2018 [1]. The three main BC subtypes, namely luminal, Her2, and triple negative (TNBC), have been defined based on the expression of estrogen receptor (ER), progesterone receptor (PgR), and epidermal growth factor receptor, ErbB2/Her2 [2, 3]. BC is a remarkably heterogeneous disease and molecular profiling has revealed a high level of diversity even within the same tumor subtype. However, this diversity represents a major challenge for tumor stratification, accurate patient diagnosis, and targeted treatment [4, 5]. While studies of the transcriptome and genome of BC have been conducted, the differential protein composition of breast tumors, in particular the secreted/extracellular protein complement [6, 7], has not been thoroughly investigated.

A large body of research has established that tumors represent complex systems in which numerous cell types, including inflammatory, immune, smooth muscle, and adipocyte cells, mediate varied interactions to ensure tumor survival and development [5, 8]. These signaling‐mediated, multidirectional interactions inside the tumor‐stroma milieu are facilitated via the tumor interstitial fluid (TIF). As part of the tumor microenvironment, the TIF permeates the interstitial tumor space and forms an interface between circulating bodily and intracellular fluids. TIF also serves as a transport medium for nutrients, discarded cellular waste, and as a storage space for signaling substances which are synthesized locally or which are brought to organs through the circulation [9, 10]. Molecular complements of tumor‐proximal fluids accumulate within the interstitial tumor space via classical endoplasmic reticulum/Golgi pathways [11], via noncanonical protein secretion [12], or through the shedding of membrane vesicles (i.e., exosomes) from intracellular compartments [13, 14]. Genesis, turnover, and drainage of TIF depend on many different factors, including tumor type, grade, and stage, as well as the composition of the tumor microenvironment. All of these factors are implicated in the regulation of tumor ecosystems and, therefore, are predicted to have a profound influence on the neoplastic progression of lesions. In recent years, increasing attention has been directed toward analyses of cancer secretomes. Accordingly, proximal lesion sampling, in combination with ‐omics profiling of TIF is currently considered a promising approach for gaining a greater understanding of the signaling events which underlie BC biology. Furthermore, it is hypothesized that deep proteomic analyses of TIF can lead to the identification of novel protein markers for breast tumor stratification and could form the basis for the development of new blood‐based disease diagnostics.

Blood is the most commonly analyzed clinical biospecimen, and it is considered a promising resource for the screening and diagnosis of various pathologies, including cancer. Obtaining a blood sample can also be minimally invasive for a patient [15]. However, despite tremendous efforts, no robust BC‐associated protein biomarkers in blood have been implemented into clinical practice, mainly due to difficulties in monitoring tumor heterogeneity and the very high dilution factor of a potential biomarker in blood [15]. Over the past decade, several research labs, including our own, have explored the hypothesis that biomolecules which are aberrantly externalized by breast tumor cells and stromal cells into the tumor interstitium are present at higher, detectable levels within the local tumor space [7, 9]. It has also been predicted that cancer‐related alterations specific to tumor development can be more prominent in TIFs than in nonmalignant interstitial fluids harvested from the same patient. Therefore, we hypothesize that biomarker signatures, which can be identified from the breast tumor secretome, can be used to establish a tumor‐specific, noninvasive blood‐based test for monitoring breast malignancy [7, 9].

In recent years, we have conducted a number of extensive studies to establish standard operative procedures for the collection and analysis of biomolecular complements in proximal fluids recovered from tumorigenic and normal breast tissues [9, 16]. We have also performed several types of quantitative ‐omics profiling studies with the aim of characterizing levels of cytokines, micro‐RNAs, and N‐glycans in breast TIF and corresponding serum [17, 18, 19, 20]. In addition, we have explored the proteome of proximal breast fluids with gel‐based proteomics coupled with mass spectrometry and immunohistochemistry (IHC). In the course of these studies, we have generated several representative proteome datasets, which contain complementary information regarding the secretome of breast tumor lesions, normal mammary glands, and a number of benign breast lesions [6]. We have identified a set of 26 proteins, which are upregulated in breast tumors as compared to normal and benign counterparts, and the expression levels of nine of these proteins were validated in an independent set of 70 malignant breast carcinomas of various grades of atypia [6]. Two‐dimensional gel/MALDI‐TOF‐based proteomics has also been applied by our group to mammary adipose tissues and corresponding interstitial fluids with the aim of investigating the role of adipocytes and related molecular circuitry in the breast tumor microenvironment [21]. However, since gel‐based proteomics mainly detect proteins present at moderate to high abundance [22], a more extensive characterization of the breast tumor secretome requires more sensitive tools. The latest developments in quantitative LC‐MS/MS, in combination with advanced computational algorithms and bioinformatics, can provide much better proteome coverage, as well as more robust protein identification. Therefore, multiple high‐throughput BC proteomics studies have emerged over the last decade [23, 24, 25]. However, most studies conducted so far have focused on profiling tumor tissues [26, 27, 28] or serum samples [29, 30]. To the best of our knowledge, only one pilot study conducted by Raso *et al*. [31] applied tandem mass tags (TMT) quantitative mass spectrometry combined with the MudPIT technique to breast TIF samples, which were isolated from three patients (two patients with infiltrating ductal carcinomas and one patient with a phyllodes tumor) [31]. In the latter study, the authors identified ~ 1700 proteins and demonstrated that this approach could be used to discriminate between normal and tumoral interstitial fluid samples. However, the number of proteins identified was rather limited. Moreover, important tumor characteristics such as subtype, grade, stage, and impact of the tumor microenvironment were not taken into account in this analysis.

In this study, we carried out a detailed quantitative high‐throughput LC‐MS/MS profiling of the protein complement of interstitial fluid samples, recovered from breast tumors of three main subtypes: luminal, Her2, and TNBC as well as from nonmalignant counterparts obtained from women with untreated BC, who underwent mastectomy at the Copenhagen University Hospital. The aim of this study was to identify a panel of proteins that are externalized from breast tumor components into the local interstitial site and to identify TIF proteins associated with BC tumor status and subtype. Up‐to‐date bioinformatics methods were applied to a database containing over 8800 proteins to conduct a comprehensive, system‐wide, and quantitative characterization of breast tumor secretomes. It is anticipated that this work will lead to the discovery of novel putative serological protein markers to improve our ability to detect and stratify breast malignancies.

## 2 Methods

### 2.1 Collection and handling of clinical samples

Fresh tissue samples were collected from patients defined as high‐risk according to the guidelines of the Danish Breast Cooperative Group (DBCG, www.dbcg.dk accessed 22.10.2009). Patients had undergone a mastectomy between 2003 and 2012, and samples were collected as part of the Danish Center for Translational Breast Cancer Research program at Copenhagen University Hospital, Denmark. More details on the criteria used to define high‐risk cancer patients are reported in our previous publications [17]. Normal samples were collected from nonmalignant areas located at least 5 cm from the tumors. All of the patients presented a unifocal tumor, and none of the patients had a history of breast surgery or had received preoperative treatment (naive samples). Registered clinicopathological data for the patients were retrieved from the Department of Pathology, Rigshospitalet, Copenhagen University Hospital. This study was conducted in compliance with the Helsinki II Declaration and written informed consent was obtained from all participants. The procedures of this study were approved by the Copenhagen and Frederiksberg regional division of the Danish National Committee on Biomedical Research Ethics (KF 01‐069/03).

At the time of collection, tissue specimens were divided into two pieces. One piece was stored at −80 °C and subsequently prepared as a formalin‐fixed paraffin‐embedded (FFPE) sample to undergo histological characterization, tumor subtyping, tumor infiltrating lymphocyte (TIL) scoring, and IHC analysis (see below). The second biopsy piece was placed in PBS at 4 °C within 30–45 min of surgical excision and then was subjected to interstitial fluid recovery (see below).

### 2.2 IHC of tissue biopsies: histological assessment and tumor subtyping

FFPE blocks prepared from two or three different parts of a tissue specimen were subjected to IHC analysis as previously described [6]. Then, tissue morphology, tumor cell composition, and tumor‐stroma percentages were evaluated as previously described [17, 32]. The BC subtype of each tissue sample was determined based on ER, PgR, Her2, and Ki67 status, in accordance with St. Gallen International Breast Cancer Guidelines [33]. Three major BC subtypes were identified: luminal, Her2, and TNBC. Due to the small number of samples available, the luminal type tissues analyzed in the present study included both luminal A and B subtypes as a merged category. The cutoffs used for ER, PgR, Her2, and Ki67 for tumor stratification were previously described [19]. The antibodies used in this study (including vendor, origin, dilution, and scoring criteria) are summarized in Table S1. Two researchers (IIG, PSG) blindly reviewed all IHC staining. For each staining, a positive control slide was included in accordance with the manufacturer's instructions. For a negative control, slides were incubated with PBS instead of primary antibody.

Information regarding all of the patients included in this study [i.e., patient age, tumor size, grade, receptor status, stratification of tumor subtype, and proportion of immuno‐infiltrate within corresponding biopsies (see below)] is summarized in Table S2. A commercially available tissue microarray (TMA) containing normal tissues from 33 human organs was used (Pantomics, Inc., San Francisco, CA, USA).

### 2.3 Evaluating and scoring TILs within tumor samples

We examined the most prominent components of the immune microenvironment (i.e., TILs) in the corresponding tumor biopsies used for breast TIF recovery. The number of lymphoid cells present was evaluated with hematoxylin and eosin staining as previously described [17], and with IHC staining with antibodies raised against CD45 (clone 2B11+PD7/26, DAKO) (Table S1). The proportion of TILs in the tissue sections were evaluated in accordance with recommendations of the International TILs Working Group 2014 [32]. Total leukocytes were scored as: 1+ (> 10%), 2+ (10–50%), or 3+ (> 50%). For immune cell population, the expression results were classified as low (neg and 1+) or high (2+ and 3+) (see details in Ref. [17]).

### 2.4 Interstitial fluid recovery

Tumor interstitial fluid was extracted from fresh breast tumor specimens as previously described [9]. Briefly, 0.1–0.3 g clean tissue was cut into small pieces (~ 1 mm^3^ each). After the tissue pieces were washed twice in cold PBS to remove blood and cell debris, they were incubated in PBS at 37 °C in a humidified CO_2_ incubator. After 1 h, the samples were centrifuged at 200 ***g*** and 4000 ***g*** for 2 min and 20 min, respectively, both at 4 °C. The supernatants were aspirated, and total protein concentrations were determined with the Bradford assay [34]. The same procedure was used to recover interstitial fluids from lesions enriched with both nonmalignant epithelial and adipose cells, which were dissected approximately 5 cm from a tumor margin. Corresponding normal interstitial fluid (NIF) and fat interstitial fluid (FIF) samples were prepared from 20 and 12 corresponding dissected tissue specimens and pooled for further analysis. To ensure minimum contamination by structural proteins that may originate from cell or tissue lysis, TIF, NIF, and FIF samples and corresponding tissue biopsies were originally subjected to comparative 2D‐gel electrophoresis in combination with MS analysis, as previously described [9, 16]. The protein component of breast TIF was found to be greatly depleted of structural and nuclear proteins. Quantitation of the ratios of several proteins known to be externalized from tumor tissue to three cytokeratins (CK14, 18, and 19) in both TIF samples and in corresponding whole tumor lysates yielded values that differed by a factor of 10 or more confirming that the release of nonspecific proteins due to cell death is not a significant contributor to TIF.

### 2.5 LC‐MS/MS proteomic experiments

#### 2.5.1 Sample preparation and TMT labeling

Samples were applied to 5 kD cutoff filters (Agilent Technologies, Santa Clara, CA, USA) to perform buffer exchange. Then, 5× the sample volume of 50 mm Hepes buffer (pH 7.6) was added to each sample. The filters were then centrifuged for 20 min at 2000 ***g*** and the flow through was discarded. This step was repeated three times to ensure that a complete exchange was achieved. Protein concentrations of the collected samples were subsequently determined with a DC Protein assay (Bio‐Rad, Hercules, CA, USA). The volume of each sample containing 30 μg protein was adjusted to 120 μL with the addition of 50 mm HEPES (pH 7.6). The samples were subsequently denatured at 99 °C for 5 min. Reduction and alkylation were performed by adding 13 μL of 100 mm dithiothreitol and 20 μL of 100 mm iodoacetamide to each sample. Tryptic digestion was performed overnight at 37 °C (trypsin:sample ratio, 1 : 60), followed by TMT labeling, according to the manufacturer's instructions (Thermo Scientific, Waltham, MA, USA). After digestion, 5 μL of each sample (TIF, pooled NIF and FIF) was taken off and run on a short gradient LC‐MS/MS for quality control. Pooled NIF and FIF samples were then dissolved in 15 µL of mobile phase A (95% water, 0.1% formic acid) and 1 μL and subjected to LC‐MS/MS analysis by using a hybrid Q‐Exactive mass spectrometer (Thermo Scientific) as described in 2.5.3. To create an internal standard to link the four TMT sets, a pooled internal standard from TIFs was prepared by taking 4 μg from each sample. TIF samples for in‐depth analysis were subjected to TMT labeling according to the manufacturer's instructions (Thermo Scientific). The four TMT‐labeled sets were then desalted and cleaned up by applying them to Strata SCX cartridges, according to the manufacturer's instructions (Phenomenex, Torrance, CA, USA), followed by lyophilization. The samples were stored at −20 °C until further analyzed.

#### 2.5.2 Peptide isoelectric focusing and extraction (HiRIEF)

After clean up, the TMT‐labeled samples underwent isoelectric focusing (IEF) on four 24‐cm, 3.7–4.9 immobilized pH gradient (IPG) strips (GE Healthcare, Uppsala, Sweden). Briefly, samples were rehydrated in 8 m urea with bromophenol blue and 1% Pharmalyte (GE Healthcare), loaded onto IPG strips, and separated, according to previously published protocols [35]. The IPG strips were subsequently subjected to passive elution with MilliQ water into 72 fractions by using an in‐house robot. The obtained fractions were dried with a SpeedVac and stored at −20 °C.

#### 2.5.3 LC‐MS/MS

For each LC‐MS analysis of a HiRIEF fraction, the auto sampler (Ultimate 3000 RSLC System; Thermo Scientific Dionex) dispensed 15 µL of mobile phase A (95% water, 5% dimethyl sulfoxide, 0.1% formic acid) into the corresponding well of a 96‐well V‐bottom polystyrene microtiter plate (Corning, New York, USA). After mixing the samples added to the plate by aspirating/dispensing a 10‐µL volume 10 times, a 7‐µL aliquot was injected onto a C18 guard desalting column (Acclaim Pepmap 100, 75 µm × 2 cm, NanoViper, Thermo Scientific). After 5 min with the loading pump at a flow rate of 5 µL·min^−1^, the 10‐port valve switched to analysis mode with the NC pump providing a flow rate of 250 nL·min^−1^ through the guard column. The curved gradient (curve 6 in chromeleon software, ThermoFisher Scientific, Waltham, MA, USA) was subsequently applied with 3% mobile phase B (95% acetonitrile, 5% water, 0.1% formic acid) increased to 45% mobile phase B over 50 min, followed by a wash with 99% mobile phase B and re‐equilibration. The total LC‐MS run time was 74 min. A nano EASY‐Spray column (Pepmap RSLC, C18, 2 µm bead size, 100 Å, 75 µm internal diameter, 50 cm length; ThermoFisher Scientific) was used on the nano electrospray ionization (NSI) EASY‐Spray source (ThermoFisher Scientific) at 60 °C. Online LC‐MS was performed by using a hybrid Q‐Exactive mass spectrometer (Thermo Scientific). FTMS master scans with 70 000 resolution and a mass range of 300–1700 *m/z* were followed by data‐dependent MS/MS at 35 000 resolution for the top five ions by using higher energy collision dissociation (HCD) at 30% normalized collision energy. Precursors were isolated with a 2 *m/z* window. Automatic gain control targets were 1e6 for MS1 and 1e5 for MS2. Maximum injection times were 100 ms for MS1 and 450 ms for MS2. The entire duty cycle lasted ~ 2.5 s. Dynamic exclusion was used with 60 s duration. Precursors with an unassigned charge state or a charge state of 1 were excluded. An underfill ratio of 1% was used. MS/MS data were searched by using Sequest HT of the proteome discoverer 1.4 software platform (Thermo Scientific) against the UniProt protein sequence database (140407) with a 1% peptide false discovery rate (FDR) cutoff. A precursor mass tolerance of 10 p.p.m. and product mass tolerances of 0.02 Da were used. Additional settings were as follows: trypsin with 1 missed cleavage; IAA on cysteine, TMT on lysine, N‐terminal as fixed modification, oxidation of methionine, and phosphorylation of serine, threonine, or tyrosine as variable modifications. Quantitation of TMT 10‐plex reporter ions was performed by Proteome Discoverer (Thermo Scientific) on HCD‐FTMS tandem mass spectra by using an integration window tolerance of 20 p.p.m. FDR rate was estimated by using percolator (part of PD 1.4). The mass spectrometry proteomics data obtained have been deposited into the ProteomeXchange Consortium2 via the PRIDE partner repository with the dataset identifier, PXD001686.

### 2.6 Normalization of samples, data filtering, and batch corrections

We quantified peptides with samples by using a pooled internal standard. Sample ratios were corrected based on mean protein abundance. Normalization was performed in Sequest (Proteome Discoverer User Guide, Software Version 2.2, XCALI‐97808, June 2017; Thermo Fisher), after which the data were log2 transformed for bioinformatics analyses. The proteomics data were filtered to remove proteins for which more than 12 samples had missing values (which is the size of the TNBC group) in order to improve the statistical power of the analyses. Missing value imputation with least local squares was performed to infer the remaining missing values before analysis [36]. Since the LC‐MS/MS experiments were performed with samples split into four different pools, we explored potential batch effects with a multidimensional scaling (MDS) analysis using Euclidean distance. For visualization purposes, we performed batch correction by using the Combat function [37] implemented in the R‐package, SVA, to remove technical pool variation. For differential abundance analysis (DAA), LC‐MS/MS pools were used as covariates within the design matrix. We carried out Least Absolute Shrinkage and Selection Operator (LASSO) and random forest (RF) analyses using both batch‐corrected and non‐batch‐corrected data, see Fig. S1 for a comparison of group‐wise variances and clustering of samples before and after correction for batch.

### 2.7 Hierarchical clustering

Hierarchical clustering was applied to batch‐corrected data according to Ward's clustering method [38] and algorithm ward.D2. This initial analysis was performed to identify covariates which might contribute to patient stratification and guide the design of DAA (see Results for additional details).

### 2.8 Evaluation of relevant hits

To evaluate the validity of protein candidates identified as potential serological biomarkers, we investigated their presence or absence in the following relevant publicly available databases and datasets:

‐ Human Plasma PeptideAtlas [39] https://www.hupo.org/plasma‐proteome‐project. We downloaded a total of 3529 plasma proteins from https://db.systemsbiology.net/sbeams/cgi/PeptideAtlas/buildInfo?_subtab=2.
‐ ExoCarta database [40] http://www.exocarta.org. At the time of our download, this database included entries for 9769 proteins secreted via the exosomal pathway (derived from 286 studies).
‐ Microparticles from human plasma [41]. This dataset includes 2357 proteins derived from twelve samples.
‐ A comprehensive dataset of proteins secreted from eleven breast cancer cell lines. This dataset includes 3386 entries and was downloaded from [42].
‐ MS/MS‐based dataset of breast TIF proteins derived from six samples from three patients. Approximately 1000 proteins are available [31, 43].

### 2.9 SignalP and Phobius

FASTA sequences of 6763 proteins (see above) were queried based on UniProt ID, with the R‐package, protr [44]. Sequences of 6582 of these entries could be retrieved and were included in our analyses. The remaining proteins (*n* = 181) were not included due to redundancy or discontinuation of their UniProt ID. Signal peptides were predicted from fasta by using SignalP V. 4.1 [45] (http://www.cbs.dtu.dk/services/SignalP/) and Phobius [46] (http://phobius.sbc.su.se/). For Signal P analysis, the default cutoff of the mean signal peptide score (*S*_mean_) was > 0.3 to define a signal.

### 2.10 Differential abundance analysis

The statistical software, limma (linear models for microarray data) implemented in r, is powerful for small sample sizes due to shrinkage of feature‐specific variances [47]. A number of studies have demonstrated the versatility of this software for the analysis of different ‐omics data, including proteomics data [48, 49]. Here, DAA used a corrected *P*‐value (FDR ≤ 0.05) as the cutoff for significance, as well as log‐fold change (logFC) ≥ 1 (±5%) or ≤ −1 (±5%) for up‐ or downregulated proteins, respectively. In our DAA, the following groups were used for comparisons: (a) all pairwise subtype combinations, (b) hormone receptor status (ER, PgR, Her2), (c) high TIL status (2+ and 3+) versus low TIL status (neg and 1+), and (d) high‐grade tumors (3) versus low‐grade tumors (1 and 2). Based on the hierarchical clustering, we also included information on LC‐MS/MS pools as contrasts in the design matrices for DAA.

### 2.11 LASSO regression

We performed LASSO regression with tenfold cross‐validation, as implemented in the r‐package, glmnet [50]. We used contrasts from the differential expression analysis which yielded significant hits, including (a) BC subtype, (b) TIL status, and (c) hormone receptor status of ER and PgR as a response. The full set of proteins retained after filtering were used as input for the LASSO regression. We ran each LASSO model 10 times with 10 different random seeds and extracted the overlap of selected proteins across runs to a consensus set. For the models with BC subtype, estrogen receptor status, and progesterone status, we split the dataset into a training set and test set, which was used to estimate the model accuracy—See Table S3. We did not split the dataset for LASSO regression with Her2 receptor, degree of TILs and tumor grade, as this resulted in large cross‐validation errors. The higher error rates observed for these models were in part related to a highly unbalanced number of samples within each clinicopathological group, especially for the Her2 receptor, in combination with large variances within these homogeneous groups. As such, we can only provide cross‐validation errors for these models, see Table S3. N.B we are aware that not splitting the datasets is bias and will produce over‐fitted models; however, here it is important to note that we do not use the results of LASSO regression as a stand‐alone method for selecting proteins, but as a way of filtering results from differential abundance analysis.

### 2.12 Random forest

We performed RF with the contrasts from the DAA, which yielded significant hits, including (a) breast cancer subtype; (b) TIL status, and (c) hormone receptor status of ER and PgR as a response. All proteins retained after filtering were used as input for the RF models. For the datasets which converged, defined by a class error ≤ 25%, variable selection was performed by re‐running the RF 10 times with different random seeds. The results of each run were overlapped to obtain a consensus of protein classifiers. The R‐packages, random Forest and varSelRF [51, 52], were used to conduct RF classification. Similarly to the LASSO regression, we split the datasets into training and test sets for models with BC subtype, estrogen receptor status, and progesterone receptor status. However, for the models with Her2, TILs, and tumor grade, we used all data for training due to the large out‐of‐bag (OOB) errors associated with these. Table S3 contains OBB errors and accuracies for the six RF models.

### 2.13 Protein‐protein interaction networks

All human protein‐protein interaction pairs from the STRING database V11.0 [53] (https://string‐db.org/cgi/download.pl?sessionId=sm2jwqiNPyyz) were downloaded and used for analysis. In order to get the most comprehensive networks all protein‐protein interactions (PPIs) with a score (support/confidence of interaction) above the lower 25th quantile of all scores were kept for analysis. Networks were created for sets of differentially abundant proteins from contrasts with BC subtypes (luminal, Her2, and TNBC). PPIs were retained in a given network if both proteins in the pair were significantly differentially abundant in the same contrast. Networks were visualized using cytoscape V3.8.0 [54], nodes were colored according to logFC, edges according to the directionality of node pair, and edge width in accordance with score (confidence) of interaction.

### 2.14 Comparison of quantitative proteomics with IHC scoring

Adjacent heatmaps of protein abundances from high‐throughput MS/MS were generated, along with IHC scores obtained from paired tumor tissues (see IHC of tissue biopsies: histological assessment and tumor subtyping). While the latter values were discrete (i.e., 0–3), protein abundances from MS/MS data were continuous. The level and expression pattern for the set of selected proteins that revealed subtype‐specific differential TIF abundancy quantified by LC‐MS/MS were blindly inspected and compared across the two plots (i.e., IHC vs. LC‐MS/MS). IHC scores of the proteins from luminal versus TNBC sample comparisons were evaluated with Fisher's exact test. Her2 subtypes were not included in the test due to the low number of available samples. Significance was defined as a *P*‐value < 0.05, and no correction for multiple testing was needed since only 10 tests were performed.

### 2.15 Protein biomarker sensitivity and specificity

Validation of subtype‐specific expression of the TIF proteins identified in this study was performed using the area under the curve (AUC) of receiver operating characteristic (ROC) curves on the independent dataset published [26]. The data from the Tyanova *et al*. [26] publication were generated using the super‐SILAC mass spectrometry technique, and the dataset acquired for ROC analysis, contained normalized H/L ratios between the standard and the tissue. Normalization of data had been performed using the maxquant software, see [26] for specifics. Any missing values were imputed by using the llsImpute function of the R‐package, pcaMethods [36], with k neighbors values of 4–7 yielding consistent results. Data were log2 transformed to push the protein abundance toward a normal distribution. After imputation of the missing values, and transformation, pROC [55] and nnet [56] were used to generated AUCs with 95% confidence intervals for: (a) each protein individually for relevant pairwise comparisons, (b) proteins combined for the relevant pairwise comparisons, and (c) proteins combined for all three groups together (multinomial model). For the latter setup, we split the data into a training set (which included 2/3 of the data) and a test set (which included 1/3 of the data). A few different seeds were selected as the starting point for splitting in order to evaluate variability among the samples within the dataset and AUC stability. The advantage of this score is that it is independent of threshold selection. Note that AUC varies between 0 and 1 and that an AUC score of 1 means perfect biomarker classification of a subtype.

### 2.16 Data and script availability

All the scripts, code, and documentation to reproduce our bioinformatic and biostatistical analyses are reported in the GitHub repository https://github.com/ELELAB/Proteomics‐TIF. The repository also contains the data and the outputs of the analyses. Moreover, we have published the Cancer BioMarker Prediction Pipeline (CAMPP) [19], a pipeline which may be used to perform most of the analyses.

## 3 Results and Discussion

### 3.1 An overview of the TIF proteome

**Fig. 1:**
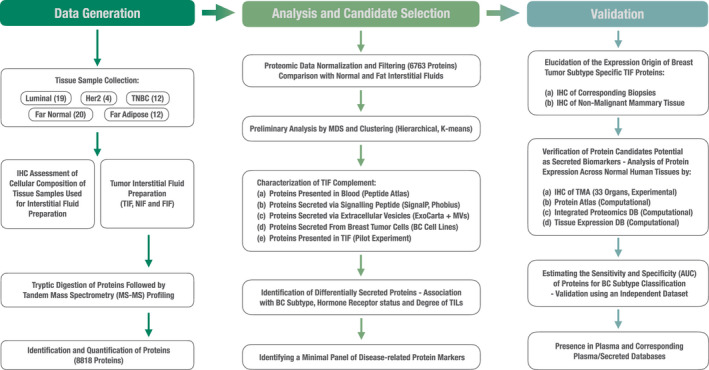
A flow chart of the experimental and computational workflow for this study. Number of tumor interstitial samples used for analysis, sample curation, and data filtering are summarized. Methodological steps in the bioinformatic/biostatistical analyses performed, along with validation (both experimental and in relation to available literature) of candidate proteins, are also presented.

To obtain an initial, yet comprehensive, characterization of the TIF proteome, we performed high‐throughput LC‐MS/MS quantitative proteome profiling of TIF samples recovered from 35 tumor specimens originating from BC patients. Our aims were to elucidate whether the composition of these TIF proteins: (I) can be used for patient subgroup stratification, (II) is dependent on the composition of the tumor microenvironment,which is known to play an essential role in tumor development, and (III) can prove useful for a putative noninvasive BC test. The experimental and computational workflow for this study is summarized in Fig. 1.

A total of 8855 proteins were identified. At a 1% peptide FDR, this coverage represents approximately six orders of magnitude of dynamic range. After normalization, filtering, and batch correction, 6763 proteins were retained, and these were included in our downstream analysis (see Fig. 1). To the best of our knowledge, this is the most comprehensive dataset of proteins externalized from breast tumors. It is worth noting that the number of proteins comprising the breast TIF dataset is less than the number reported in the largest breast tumor proteomic dataset available to date, which includes 10 135 proteins identified by high‐throughput LC‐MS/MS screening of whole breast tumor tissue samples [26].

**Fig. 2:**
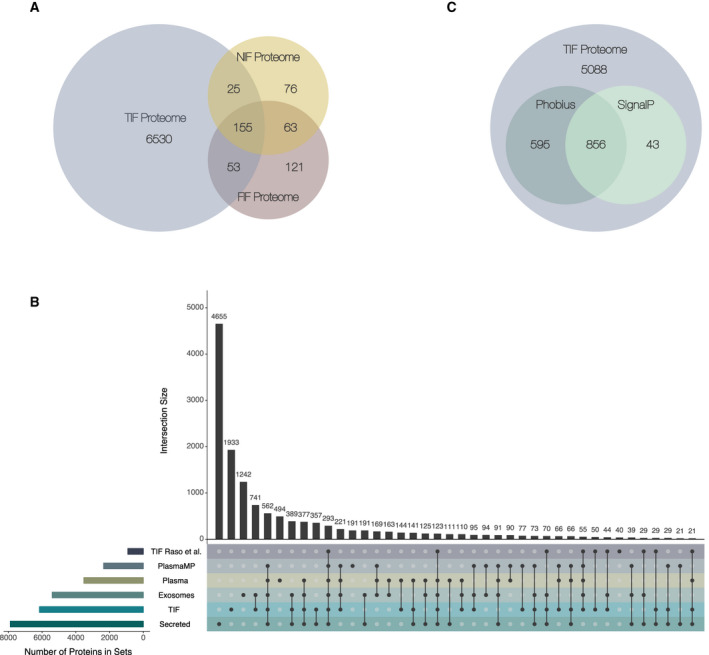
Overall characterization of the TIF dataset. Both a comparison of secretome‐related datasets and an evaluation of the secretion pathway were performed. (A) Venn diagram of the overlap between proteins identified within TIF samples and within pooled NIF and FIF samples. (B) An Upset plot illustrates the overlap of the final set of 6763 TIF proteins, 6066 unique gene symbols, included in further analyses with: (a) human plasma—PeptideAtlas database proteins [39]; (b) human exosomal proteins from the ExoCarta database (http://www.exocarta.org; [40] (c) a dataset of proteins associated with circulating human plasma microparticles [41]; (d) a secretome of 11 BC cell lines of different origins [42]; and (e) an interstitial fluid BC protein complement published by Raso *et al*. [31]. Colors denote the individual protein datasets. Horizontal bars indicate the size of each dataset; vertical bars indicate the number of proteins shared between all combinations of the six sets. (C) Venn diagram showing how many TIF proteins are predicted by SignalP [45, 103] and/or Phobius [46] to encompass a signal peptide.

In parallel with the quantitative profiling of proteins externalized from tumor masses, we examined the protein composition of interstitial fluids recovered from far‐distant tumor lesions containing a high proportion of nonmalignant mammary epithelium or adipose tissue (i.e., NIF and FIF samples, respectively) (see Methods). Protein spectra from NIF/FIF samples were analyzed with lower analytical depth and proteome coverage, thereby yielding the most abundant proteins externalized. We considered this to represent a baseline of normalcy. Within the pooled NIF and FIF samples, 318 proteins and 391 proteins were detected, respectively (Fig. 2A). A total of 155 proteins were common to all of the TIF, NIF, and FIF samples, and this subset represents approximately 50% of all the NIF and FIF proteins identified. In addition, 53 proteins are shared between the TIF and FIF samples, while 25 proteins are shared between the TIF and NIF samples. Meanwhile, 260 proteins in the FIF and/or NIF samples were not identified in any tumor fluids (Fig. 2A, Table S4).

To gain further insight into the secretion potential of proteins identified in our TIF samples, we compared our dataset to secreted protein entries in the five most representative and relevant protein datasets and databases currently available:

‐ The Human Plasma PeptideAtlas database [39] is the most comprehensive resource of proteins present in human blood, independent of origin and type of secretion. A comparative analysis between TIF and plasma protein complements has the potential to identify secreted proteins that enter the blood circulation, and thus, may assist in prioritizing candidates for further studies. In total, the concatenation of datasets from this database yielded 3529 proteins for analysis.
‐ The ExoCarta database [40] is the largest database of exosomal proteins, containing more than 40 000 protein entries (9769 proteins). Exosomes are small membranous vesicles (30–150 nm in diameter), which are released by a variety of cells into the extracellular environment. Exosomes represent a nonclassical, vesicle‐mediated secretory pathway for the transport and exchange of a variety of biomolecules between cells as a means of communication. Thus, a comparison of TIF with ExoCarta enabled us to identify proteins which are most likely externalized into breast TIF through exosome‐associated secretion pathways.
‐ A dataset of proteins associated with circulating human plasma microparticles (MPs) [41]. Plasma and other bodily fluids contain membranous MPs, which are thought to be derived from various cell types, including BC cells [57]. MPs differ from cellular exosomes in size and cellular origin, with the latter originating from intracellular multi‐vesicular bodies. The importance of MPs as mediators of cellular signaling is supported by recent data which demonstrate that MPs serve as vectors in the intercellular transfer of functional proteins and nucleic acids, and also in drug sequestration [58]. Moreover, an important role for MPs in facilitating evasion of cancer cell immune surveillance has been demonstrated [59]. In total, 2357 proteins associated with MPs are currently available for analysis.
‐ A comprehensive dataset of secreted proteins from 11 BC cell lines with different origins that are representative of different stages of BC development [42]. This is the most comprehensive dataset of proteins detected in conditioned media of BC cells, and it represents the major BC subtypes. This dataset encompasses 3386 proteins.
‐ The only high‐throughput LC‐MS/MS‐based pilot study of breast TIF protein composition published to date [31]. This dataset contains approximately 1000 proteins and derived from an analysis of breast TIF samples from three healthy individuals and three patients with tumors.

The results from comparing these datasets are presented in Fig. 2B. Overlaps (i.e., intersections) between the proteins found in the different datasets are indicated with vertical bars. In total, 4830 out of the 6763 proteins (71.4%) detected in the TIF samples were included in at least one of the datasets used for comparison. The main overlap observed involved exosomal proteins present in the ExoCarta database, with 3203 proteins found to be present in TIF and in exosomes. This result indicates that many of the TIF proteins (~ 50%) are likely externalized through exosomal signaling pathways. In addition, 2567 proteins were present in TIF and secreted from BC cell lines, while 2230 proteins and 1599 proteins were found to be shared between TIF and plasma or plasma MPs, respectively. Taken together, these results highlight the secretory nature of the TIF proteome complement. Compared with the TIF dataset published previously by Raso *et al*. [31], 775 proteins are common to the present breast cancer TIF dataset. This overlap represents approximately 84% of the 924 proteins identified by this group. These results emphasize the validity of our experimental framework and they also indicate that high compliance exists between the data obtained in both of these studies on TIF.

Additionally, an important observation is that our TIF protein complement contains 1933 unique entities which do not overlap with any of the five datasets used for comparison (Fig. 2B). These proteins were potentially identified due to the depth of our analysis. A subset of these proteins may be specific to breast TIF and may originate from malignant cells and/or cells present in the tumor microenvironment. However, we cannot exclude the possibility that some of these proteins are presented in TIF as a result of partial cellular or tissue lysis during sample preparation.

After comparing our dataset to these relevant databases and datasets, we used the prediction tools, SignalP [45] and Phobius [46], to segregate classically secreted proteins within the TIF proteome from proteins externalized via nonclassical pathway(s). With the use of these two tools, the presence of signal peptides within TIF proteins was predicted. While these signal peptides may indicate which proteins are targeted for the secretory pathway, they may not necessarily drive secretion. Among the TIF proteins, 6582 out of 6763 had FASTA sequences available for analysis. According to SignalP analysis, a total of 899 TIF proteins are predicted to contain a signaling peptide. In comparison, Phobius predicted that 1451 proteins contain a signaling peptide. The overlap between the two predictors was quite high, with 856 commonly predicted secreted proteins present in the TIF proteome (Fig. 2C). However, this set of proteins only accounted for 10% of the TIF proteome (Fig. 2C), suggesting that a significant proportion of TIF proteins undergo nonclassical secretion (i.e., though membrane pore formations or via specialized secretory autophagosomes [60]).

### 3.2 TIF proteins distinguish low‐grade versus high‐grade tumors and are associated with different levels of TILs

**Fig. 3:**
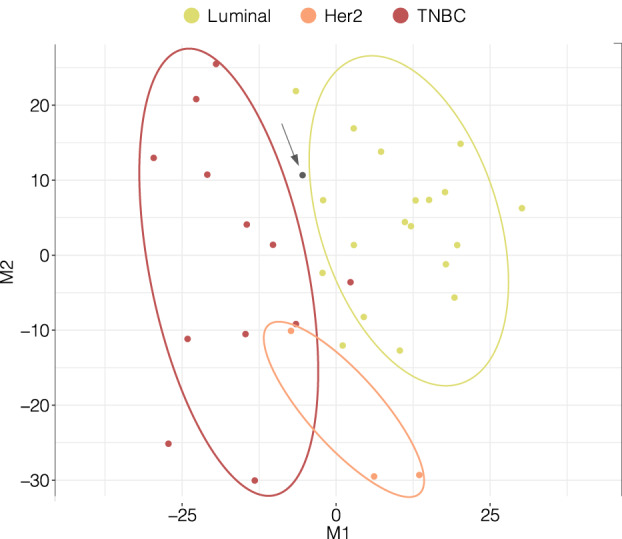
Multidimensional scaling plot of 35 BC TIF samples according to subtype based on protein abundances. The *x*‐axis and *y*‐axis denote multidimensional scaling components 1 (M1) and 2 (M2), respectively, which best retain the distance relationship (squared Euclidian) between the samples in two‐dimensional space. The single gray dot indicated with an arrow represents a Her2 outlier sample (apocrine dysplasia(s) with multiple cyst structures), which was excluded from further analysis.

To evaluate the potential for TIF proteome profiles to be segregated according to BC subtype, we stratified available tumor samples into three major subtype‐specific groups: luminal, Her2, and TNBC. Corresponding plots with multidimensional scaling (MDS) revealed considerable segregation of TIF proteomes across all three tumor subtypes (Fig. 3). A slight overlap of two samples, a Her2‐ and a TBNC‐subtype with the TBNC and luminal groups, respectively, was observed (Fig. 3). However, one Her2 sample appeared to be a very clear outlier (Fig. 3, indicated with an arrow). A morphological analysis of the corresponding tumor biopsy revealed the presence of apocrine dysplasia(s) with multiple cyst structures scattered within the tumor mass. Apocrine dysplasia tumors have a high secretion potential [6, 61], and this characteristic is consistent with the unique TIF profile of this particular Her2 tumor sample. Therefore, we removed this sample from subsequent analyses.

**Fig. 4:**
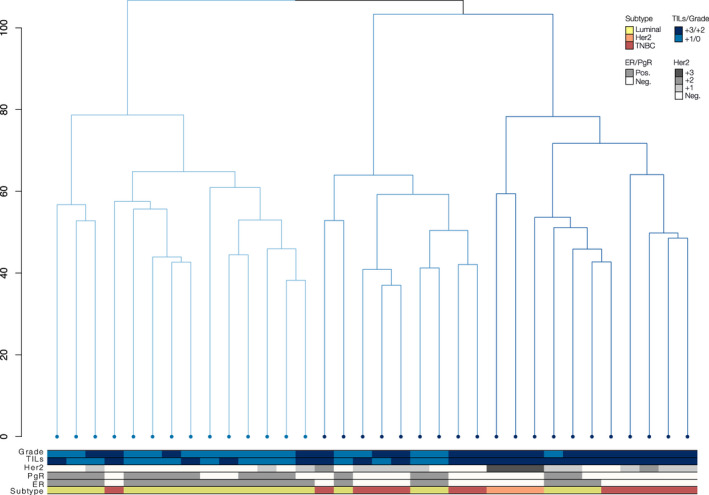
Dendrogram clustering of 34 BC samples based on TIF protein abundances. Coloring of bars as gray (positive) or white (negative) labels ER/PgR/Her2 status of the samples. Subtype, TIL grade, ER/PgR, and Her2 status are colored as indicated. TIL samples were stratified according to recommendations of the International TILs Working Group 2014 [32] and as described in Methods.

To gain insight into which clinical and morphological covariates may have an impact on the externalized protein patterns within the interstitial space, unsupervised hierarchical clustering was performed (see Hierarchical clustering). As shown in the dendrogram in Fig. 4, both hormone receptor status and level of TILs within the corresponding biopsies were able to stratify the TIF proteomes of the BC patients examined. Hierarchical clustering revealed two main clusters of TIF samples, Cluster 1 and Cluster 2. Cluster 1 almost exclusively encompassed ER+ luminal samples (93% of samples within the cluster), the majority of which were positive for PgR, and from lower grade tumors approximately, 71% of samples. Cluster 1 samples were more often characterized by a low level of immune cell infiltration (65% within‐cluster and 69% across clusters), although this pattern was more subtle. In contrast, Cluster 2 mainly consisted of ER^−^/PgR^−^ samples, 70%, and 75%, respectively, originating from the TNBC subtype (55% of samples and 92% of all TNBCs across clusters) and Her2 specimens—15% within‐cluster and 100% of Her2 samples. Most of the samples within Cluster 2 were enriched in TILs and originated from high‐grade tumors, 80% and 75%, respectively (Fig. 4). These findings are in agreement with the results of our recent publications on cytokine and N‐glycan profiling of breast tumor interstitial fluids, which demonstrate that high‐grade Her2, and especially TNBC, tumors exhibit a high level of TILs [17, 19]. Meanwhile, neither the percentage of malignant cells in the tumor samples, nor patient age, stratified the TIF proteomes. It should be noted that while there was a propensity toward the clustering of samples with similar clinicopathological characteristics. Figure 4 also highlighted sample heterogeneity, a well‐established issue within the field of breast cancer research. The distribution of different clinicopathological groups within clusters and across clusters may be found in Table S5.

### 3.3 Identification of differentially abundant (DA) proteins associated with BC subtypes, hormone receptor status, and degree of TILs

Next, we performed differential abundant analysis (DAA) to identify which secreted proteins are able to discriminate between TIFs originating from the three major BC subtypes, tumors of different grades, and tumors with varying degrees of infiltrating lymphocytes (Fig. 4). Specifically, DAA was applied to the following group comparisons: (a) all pairwise subtype combinations (i.e., luminal vs. Her2, luminal vs. TNBC, and Her2 vs. TNBC); (b) ER^+^ versus ER^−^, (c) PgR^+^ versus PgR^−^, (d) high Her2 (3^+^/2^+^) versus low Her2 (1+/0), (e) high (3^+^/2^+^) versus low (1^+^/0) TIL status, and (f) high‐grade tumors (i.e., GR3) versus low‐grade tumors (GR2/1).

From these six comparisons, a total of 174 DA proteins (FDR < 0.05 and logFC > 1 or < −1) were identified. Among these, 151 proteins were associated with BC subtypes, 64 proteins were associated with ER/PgR/Her2 status, and 15 proteins were associated with TIL scoring. Four of these proteins, ADIRF, S100A9 (both in luminal vs. TNBC), HSPB1, and POSTN (TIL associated), were found in the NIF/FIF background datasets. Despite the observed partitioning of samples based on tumor grade in Fig. 4, we did not identify any DA proteins when we compared TIF samples from high‐ versus low‐grade tumors. This result may be due to the almost total confounding of tumor grade with TIL status. Thus, when we corrected for TILs as a confounder in the statistical analysis, we lost most of the biological variance between tumor grades. The complete list of DA genes used for each comparison, as well as the directionality in the comparison (i.e., up‐ vs. downregulation), are reported in Table S6. Individual set‐wise list of DAA results, including test‐statistics, *P*‐values and logFCs, may be found in Table S7. Additionally, set‐wise heatmaps of differentially abundant proteins from each comparison are presented in Fig. S2.

**Fig. 5:**
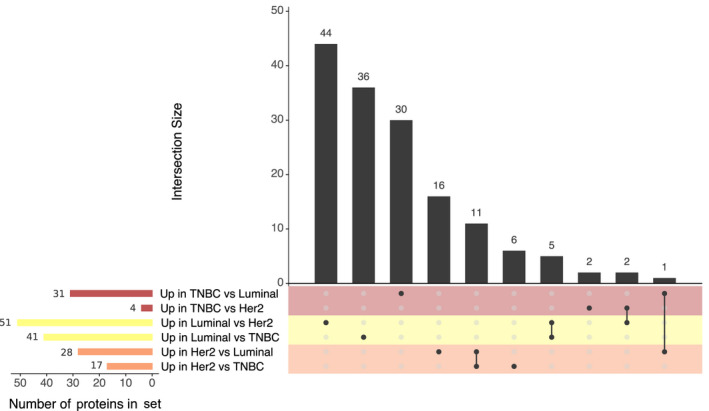
Upset plot showing the overlap of TIF proteins which exhibited DA according to BC subtype. Horizontal bars denote the size of each dataset; vertical bars indicate the number of proteins shared between all combinations of the six sets. Colors are used to indicate comparisons made with TNBC (red), Her2 (orange), or luminal (yellow) subtype samples.

Among the 151 proteins which exhibited differential abundance in the TIF samples from three BC subtypes, we identified 60 unique DA proteins when we compared Her2 and luminal samples, 66 unique DA proteins when we compared luminal and TNBC samples, and only eight DA proteins when we compared Her2 and TNBC samples. The majority of DA proteins were unique to one subtype, although a few overlapped across pairs of subtypes (Fig. 5, black vertical bars across subtype‐wise comparison). Five of the proteins (BCAM, COPS9, DNJC12, TCEAL3, and ZSCAN18) revealed higher abundances in the TIF samples with luminal origin, compared to both the Her2‐enriched and TNBC TIF samples. Meanwhile, there were 11 proteins (BCAS1, CDK12, CRYM, ERBB2, FDFT1, GRB7, HMGCS1, IDI1, MIEN1, SRCIN1, and VPS13B), which were enriched in the Her2 TIF samples compared to the luminal and TNBC TIF samples. Conversely, two proteins (LRMDA/C10orf11, TMEM51) exhibited depleted abundances in the Her2 TIF samples compared to the luminal and TNBC TIF samples, while one protein (PADI2) exhibited low abundance in the luminal TIF samples compared to both the Her2 and TNBC TIF samples. Finally, the 9 + 4 proteins identified as DA in the comparisons of high versus low Her2 and PgR^+^ versus PgR^−^ TIF samples, respectively, were redundant, with those identified in the subtype contrasts. Similarly, the majority of the 51 proteins found to be DA between the ER^+^ versus ER^−^ TIF samples were also DA between the luminal versus Her2 and/or TNBC TIF samples.

### 3.4 Identifying a minimal subset of disease‐related proteins: A consensus approach (DA analysis, LASSO regression, and random forest)

Our DAA returned a relatively large number of proteins. Therefore, we further employed two independent approaches to pinpoint the most prominent set of protein candidates, which would have the potential to discriminate different subgroups of breast tumors. These analyses included random forest (RF) classification and least absolute shrinkage and selection operator regression (LASSO) with leave‐one‐out k‐fold cross‐validation (see Methods).

For LASSO regression with BC subtypes (without Her2), estrogen, and progesterone receptors, we split the sets into training and test sets; however, for regression analysis with Her2 status, degree of TILs and tumor grade we kept all samples for training, as these models returned large cross‐validation (CV) errors even when all samples were included in the model. The larger CV errors observed for these regression models (~ 25%) were somewhat attributed to an unequal distribution of classes (subgroups), especially for Her2 status, a high degree of sample heterogeneity within these clinicopathological groups, in addition to our small sample size. Table S3 contains the variables returned from each LASSO, ordered according to the variables weight in the model, in addition to cross‐validation errors and accuracies with confidence intervals for regressions where data could be split into training and test sets. Average cross‐validation errors for models with subtype, ER, and PgR ranged from 7% to 10%, while accuracies estimated from the test sets were ~ 0.89 (CI: 0.5–0.99) for all three.

Similarly to regression analysis, random forest returned large class errors (> 25%) and poor convergence for models with Her2 receptor status, degree of TILs, and tumor grade. In contrast, convergence for RFs with BC subtype (without Her2), ER, and PgR status was okay (~ 15% misclassified), with accuracies of 94%, 89%, and 78%, respectively. See Table S3 for out‐of‐bag errors, class errors, and accuracies. Generally, RF and LASSO with Her2, TILs, and grade as outcome, had poor overlap of selected variables, and those which were identified by both approaches were weighted quite differently. This observation is supported by the large OOB and CV errors associated with these models.

**Fig. 6:**
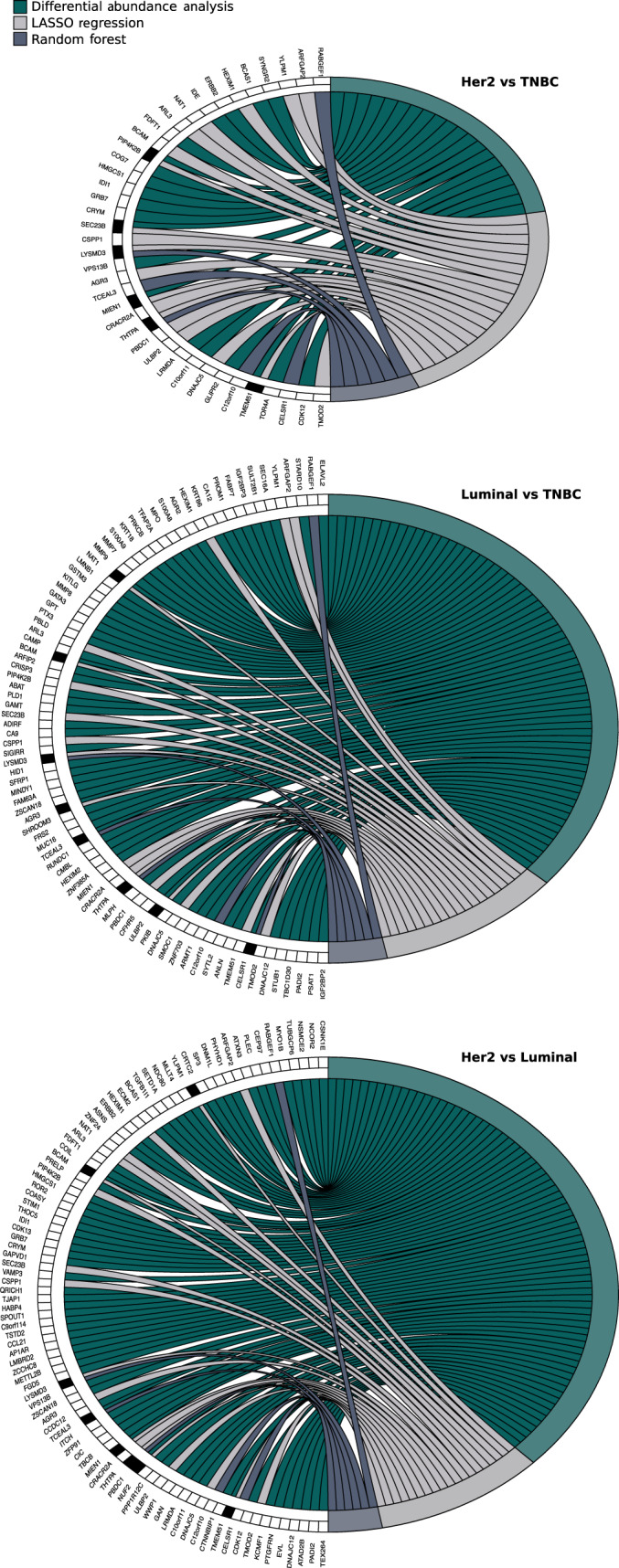
Consensus analysis of DA proteins according to BC subtype. Circle plots represent the consensus observed across DAA (green), LASSO (light gray), and RF (dark gray) analyses. Genes identified with each approach are noted on the left‐hand side of each plot. The black boxes indicate genes identified with more than one method.

We derived a list of candidates for each comparison (i.e., BC subtypes, ER/PgR/Her2 status, and TIL level) where proteins were identified by at least two out of the three methods applied (Fig. 6 and Table S8). Both RF classification and LASSO regression returned five of the original 15 DA proteins detected in the TIF samples with high versus low TILs, here among: COL5A3, HSPB1, GPC1, MAPT, and SPATA18 (Table S8). HSPB1 was excluded from the final list of proteins because it was detected in NIF/FIF samples (Table S4). RF and LASSO regression also returned protein GPC1, which had significant adjusted *P*‐values in the differential expression analysis but fell short of the logFC cutoff (logFCs −0.86 and 0.44, respectively). Accordingly, GPC1 was also excluded from the minimal consensus subset of proteins. Interestingly, all three TIL‐associated proteins exhibited an inverse directionality of correlation between their protein abundance in TIF samples and the number of TILs observed in matched tumors. Decreased abundances of COL5A3, MAPT, and SPATA18 were detected in TIF samples derived from tumor specimens with a high level of leukocyte infiltrate (+3/+2). These results imply that metabolites produced by white blood cells that penetrate tumors may suppress the production and/or secretion of these proteins by neoplastic cells. Interestingly, recent evidence suggests that one of these identified proteins, SPATA18, plays an important role in suppressing the progression of breast and colorectal cancers in a hypoxic tumor microenvironment [62, 63, 64]. Meanwhile, lower expression of MAPT in TNBC tumors (which are often enriched with TILs), and not in other BC subtypes, has been observed [65]. Darlix *et al*. [66] (and references within) have also recently demonstrated a prognostic value for the serum level of MAPT in metastatic BC patients, as well as its correlation with brain metastases).

When hormone receptor status was compared with the results of LASSO regression and RF classification, 10 proteins were identified. Six proteins were associated with estrogen status (CELSR1, SEC23B, THTPA, TCEAL3, ZNF703, ZSCAN18), two proteins were related to progesterone status (BCAM, COMP), and three proteins were related to Her2 status (ERBB2, SP3, ZNF24) (Table S8).

Out of the 24 proteins identified as a minimal subset of disease‐related proteins (Table S8), we selected 10 proteins, namely AGR3, BCAM, CELSR1, MIEN1, NAT1, PIP4K2B, SEC23B, THTPA, TMEM51, and ULBP2, which represent potential candidates for segregating TIFs from different BC subtypes. The selection was based on the commercial availability of antibodies that met the criterion for their sensitivity and specificity (Methods: IHC of tissue biopsies: histological assessment and tumor subtyping, Table S1) required for further confirmatory studies by IHC (see TIF subtype‐specific protein signatures: Expression origin and externalization pattern below).

Two of these proteins were identified in contrast with estrogen status (CELSR1, THTPA), while one was associated with progesterone status (BCAM). Based on available datasets and literature, we evaluated whether these proteins are present in human plasma, in exosomes, and/or among proteins secreted by breast cancer cell lines of different origins. In addition, we checked whether these proteins may be externalized through classical pathways based on the predicted presence of secretory signal peptides (SignalP or Phobious). Finally, to ensure that these candidate proteins originate from malignant cells and not from the normal epithelium and/or adipose cells, we investigated their presence within pooled NIF and FIF samples. None of the NIF/FIF proteins were included in the consensus set, thereby confirming the potential of this subset to serve as BC‐specific biomarkers (Table S8).

To estimate the potential significance of DA TIF proteins associated with BC subtypes, we compared them to the gene‐set of the well‐established BC classifier, PAM50. The PAM50 signature is one of the most powerful predictive and prognostic classifiers [67, 68] currently implemented in the clinic [approved by the US FDA in September 2013]. The PAM50 signature is characterized by expression levels of 50 transcripts, including mostly hormone receptors, proliferation‐related genes, and genes exhibiting myoepithelial and basal features. When we compared the DA proteins associated with BC subtypes (a total of 151 proteins, see TIF proteins distinguish low‐grade versus high‐grade tumors and are associated with different levels of TILs) to the PAM50 classifier set of genes, eight proteins (MLPH, ANLN, ERBB2, GRB7, NAT1, SFRP1, NUF2/CDCA1, and NDC80/KNTC2) were identified (Table S9). We subsequently compared the abundance directionality (up or down) of these eight TIF proteins with intratumor mRNA levels for pairwise subtype contrasts [69] and observed full concordance between mRNA expression levels and levels of the corresponding proteins (Table S9). Furthermore, only NAT1 from a minimal subset of TIF candidates (*n* = 14) identified by using a consensus approach (i.e., DAA, elastic‐net regression, and machine learning) matched the corresponding transcripts in PAM50. Tyanova *et al*. [26] previously reported a higher number of proteins (41) from BC tumor subtypes that matched the 50 transcripts of PAM50; yet only 21 had quantitative data available from more than 70 samples for which PAM50 genes enabled partial segregation of classical subtypes at the protein level. In particular, the authors highlighted four well‐described proteins for differentiating breast cancer subtypes, namely Her2, Grb7, FOXA1, and MLPH, which were clearly selected in the PAM50 and proteomic signatures. It should be noted that two of these proteins, Grb7 and MLPH, are present in our 8‐protein set, which overlaps with PAM50.

### 3.5 Protein‐protein interaction networks

**Fig. 7:**
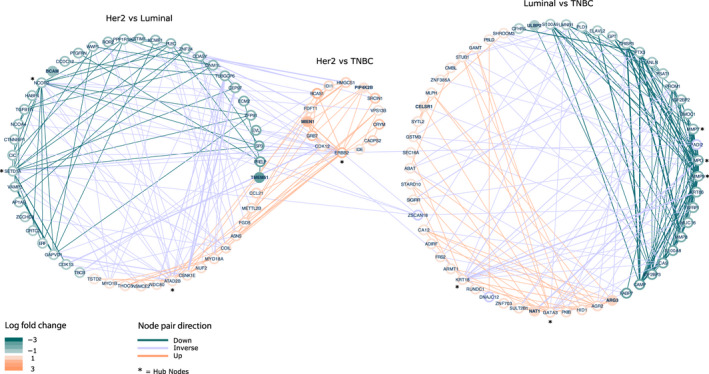
Protein‐protein interaction networks based on DA proteins from the comparison of BC subtypes (luminal, Her2, and TNBC). The plot contains three networks; (I) Her2 vs luminal, (II) Her2 vs TNBC and (III) luminal vs TNBC. Nodes (proteins) are colored according to logFC: green < −1 and orange > 1. Edges are colored based on directionality of node pair: green = both nodes down, orange = both nodes up, purple = inverse directionality of nodes. The width of edges denote the node pair interaction score (support) from STRING, and ranges from 0.25 to 1.0. Purple nodes are a part of more than one network and have opposite directionality in the networks.

To assess whether the interplay between differentially abundant proteins from contrasts with BC subtype, we constructed protein‐protein interaction (PPI) networks using the STRING database [53] and visualized with cytoscape [54] (see Methods and Table S10). The results of network analysis with DA proteins from comparisons with BC subtypes are shown in Fig. 7. Specifically, we were interested in whether protein candidates selected using the consensus approach (DAA, LASSO, and RF) were highly interconnected within the PPIs, indicating a regulatory role or potentially a driver role, or if they were leaf nodes.

The network of DA TIF proteins from the Her2 vs TNBC comparison was small and almost completely redundant with the Her2 vs luminal network—all nodes in the Her2 vs TNBC network were upregulated in Her2, see Fig. 7. All hub nodes are marked in Fig. 7 with a star. Hub nodes from the Her2 vs luminal network included ERBB2, ATAD2B both upregulated, and NCOR2, SETD1A, downregulated. ERBB2 and ATAD2B, were not hub proteins in the same subnetwork but in each their own, connecting 30 and 16 proteins, respectively. High levels of ATAD2(B) are known to be associated with increased cell survival, tumor cell migration, and a poor prognosis in patients with breast cancer, supported by multiple studies [70, 71], and in accordance with this, TIFs from Her2 samples had a greater abundance of this protein as compared to luminal samples.

The hub protein SETD1A, which was upregulated in luminal compared to Her2 samples, is a component of the histone methyltransferase (HMT) complex. SETD1A has been shown to be involved in the regulation of mitotic gene expression, and the knockdown of this gene leads to cellular senescence [72]. SETD1A is amplified in 7–24% of breast cancers and was found to promote survival and migration of ER‐positive breast cancers, specifically [73].

Inversely to SETD1A, a high level of the hub protein NCOR2 (also up in luminal vs Her2), may be associated with increased metastasis‐free‐survival in BC patients with ER‐positive tumors [74]. NCOR2 is an established tumor suppressor gene in prostate cancer and a hallmark of this cancer type (COSMIC database) [75].

Hub proteins from the luminal vs TNBC network included GATA3 and KRT18 (upregulated), both of which are well‐known markers of luminal breast cancer [76], as well as MMP7, MMP9, and MPO (downregulated). Matrix metalloproteinases MMP7 and MMP9 are thought to be drivers of tumor cell invasion and metastasis in patients with TNBC, and have therefore been proposed as therapeutic targets for drug treatment of this more aggressive type of BC [77, 78]. In accordance, the abundance of hub proteins MMP7 and MMP9 were low in luminal samples compared to TNBC.

Three proteins ZSCAN18, PADI2, and DNAJC12 connected the Her2 vs luminal network with the luminal vs TNBC network. ZSCAN18, which had a high abundance in Luminal vs Her2 and TNBC samples, was identified by the consensus method as one of the best candidates for ER+ vs ER− classification (Table S8). The role of ZSCAN18 in breast cancer is not well‐studied; however, this gene is proposed to be a strong methylation marker for colorectal, gastric, and pancreatic cancers [79]. TIFs from patients with Luminal ER+ breast tumors had a high abundance of both ZSCAN18 as well as DNAJC12. In agreement with this observation, the expression level of the DNAJC12 gene is known to be significantly positively correlated with estrogen receptor‐positive status, and may be regulated by estrogen itself via response elements in the genes promoter [80]. Inversely to ZSCAN18 and DNAJC12, the protein PADI2 was depleted in TIFs from luminal samples in comparison to Her2 tumors. Expression of the PADI2 gene, a member of the peptidyl arginine deiminase family, has been strongly linked to the amplification of Her2 (ERBB2). The inhibition of PADI2 gene expression results in a decrease in the level of cell cycle genes p21 and Ki67 [81], as well as other genes associated with aggressive breast cancer phenotypes, here among ACSL4 and BIRC3 [82]. PAD12 has been proposed as a biomarker for Her2 tumors and a potential therapeutic target for BC treatment.

Out of the 10 proteins selected as secreted candidate biomarkers for separation of BC subtypes, eight were retained in the PPI networks, while two proteins, THPTA and SEC23B, did not have any interactions annotated in STRING. Three proteins, BCAM, TMEM51, and ULBP2 were leaf nodes in their respective networks. The single interaction partner of BCAM (Her2 vs Luminal network) was ERBB2, although evidence for this interaction (score) was low, while TMEM51 was a part of a small subnetwork including PLEC, PTGFRN, and ECM2 within the larger network with hub proteins NCOR2 and SETD1A. ULBP2, included in the Luminal vs TNBC network, interacted with CAMP, which was tightly connected to the hub nodes, matrix metalloproteinases MMP7 and MMP9.

CELSR1 and NAT1, both from the Luminal vs TNBC network, had three interaction partners each (average number of interactions per node), CELRS1 was connected to SFRP1 and SHROOM3 in addition to hub node GATA3, while NAT1 interacted with GSTM3, SULT2B1, and hub node MPO.

The most interconnected of the 10 protein candidates, with 6–7 edges each, were MIEN1, PIP4K2B, and ARG3. The latter of these, ARG3 was a part of the subnetwork with hub nodes GATA3 and KRT18, along with its homolog ARG2. MIEN1 and PIP4K2B were included in the highly interconnected subnetwork with ERBB2 as the hub and interacted with each other as well a hand full of proteins, all of which were upregulated in TIFs from Her2 tumors.

### 3.6 TIF subtype‐specific protein signatures: Expression origin and externalization pattern

**Fig. 8:**
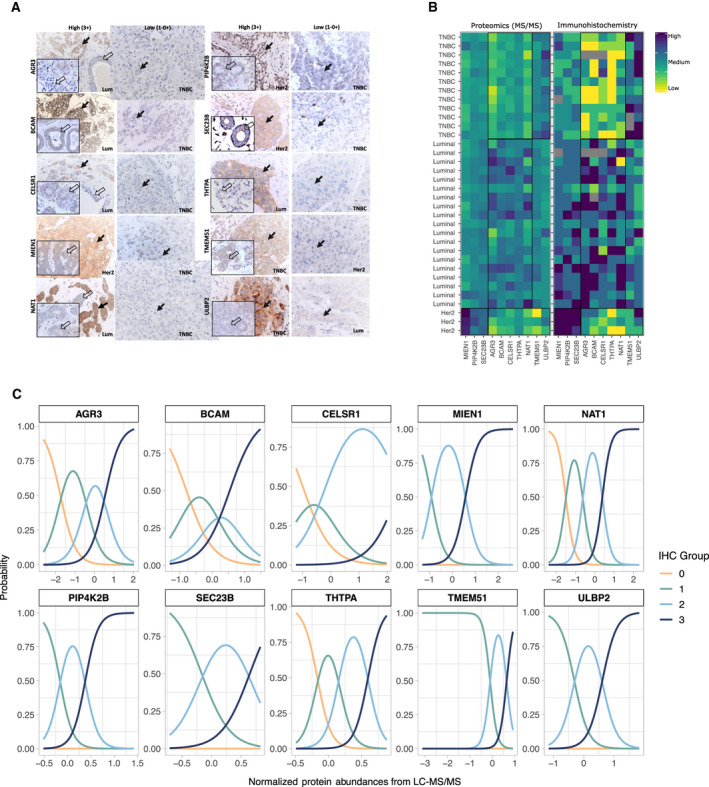
Differential intratumor and TIF abundance of 10 proteins which discriminate between BC subtypes. Intracellular expression levels of selected proteins were estimated by IHC across tumor biopsies used for TIF recovery, panel A. The expression level of each protein was considered positive if at least 10% of the tumor cells have intensities of expression scored as (0–1+), medium (2+), or high (3+), in accordance with previously described criteria (Keune *et al*. [85]). Representative examples of high (3+) versus low (0–1+) expression levels for AGR3, BCAM, CELSR1, MIEN1, NAT1, PIP4K2B, SEC23B, THTPA, TMEM51, and ULBP2 proteins are presented. BC subtypes are specified within each panel. Far‐normal areas of each tumor biopsy used for TIF recovery were analyzed in parallel and representative staining is shown (insertions within corresponding IHC panels). Black arrows within representative IHC images of the tumor biopsies indicate either positive (left panels) or negative (right panels) malignant cells. Transparent arrows indicate normal‐like mammary ducts located in close proximity of tumor cells. Magnification of images shown is 40×. Correlation between TIF and intracellular abundancy is summarized in panel B with tile plots containing: (a) batch‐corrected MS/MS‐based protein abundances of the top 10 candidates for discriminating breast cancer subtypes and (b) IHC scores of the same 10 proteins. Protein names are indicated on the *x*‐axis. Sample ID, along with assigned BC subtype, are denoted on the *y*‐axis. Coloring indicates low abundance/low IHC score (yellow), ranging to high abundance/high IHC score (blue). Black squares highlight which of the three subtypes the samples belonging to. Panel C contains logistic regression plots, one for each of the 10 proteins of interest, showing the probability of a given IHC score (*y*‐axis) in relation to normalized TIF protein abundance (*x*‐axis). Colors denote IHC scores of 0 (0–0.5), 1 (1–1.5), 2 (2–2.5), and 3.

To elucidate whether selected protein candidates with subtype‐specific patterns originate from malignant cells, normal cells, or TILs, we performed an extensive IHC analysis of 10 proteins, including anterior gradient protein 3 (AGR3), lutheran/basal cell‐adhesion molecule (BCAM), Cadherin EGF LAG seven‐pass G‐type receptor 1 (CELSR1), membrane‐anchored protein C35 (MIEN1), *N*‐acetyltransferase 1 (NAT1), phosphatidylinositol‐5‐phosphate (PtdIns5P)‐4‐kinase (PIP4K2B), Sec23 Homolog A (SEC23B), Thiamine triphosphatase (THTPA), Transmembrane protein (TMEM51), and UL16‐binding protein 2 (ULBP2) (Table 1). These proteins were selected for analysis based on the availability of highly specific antibodies and their quality and specificity exhibited in a series of control experiments (Table S1). Protein expression was analyzed for both proximal and distant samples, which were collected with TIF recovery and from paired normal lesions (Fig. 8) based on stratification criteria described in Methods (see IHC of tissue biopsies: histological assessment and tumor subtyping and Table S1). Representative examples of high (3+) versus low (0–1+) expression levels within tumor samples, as well as within nonmalignant areas, are shown in Fig. 8 (panel A).

**Table 1:** Detailed information for the top 10 candidate proteins found to be DA in TIF samples obtained from luminal, Her2, and TNBC BC subtypes. Parentheses around arrows denote a protein was borderline significant (i.e., corrected *P*‐value < 0.05, and log‐fold change close to, but not quite reaching, the cutoff of −|+ 1) (a) Primary UniProt nomenclature, (b) Primary SwissProt nomenclature, (c) see Table S6 for details, (d) Arrows denote abundance directionality (up or down) in TIF samples, (e) see Table S7 and Fig. 7 for details, (f) see Fig. 7 for details, (g) see Fig. 7 for details, (h) see Table S8 for details, (↓)—borderline significant. Additional references in table are [104, 105, 106, 107, 108, 109, 110, 111, 112, 113, 114, 115, 116, 117, 118, 119, 120, 121, 122, 123, 124, 125, 126, 127, 128, 129, 130, 131, 132, 133, 134, 135, 136, 137, 138, 139, 140, 141, 142, 143, 144, 145, 146].

Gene symbol (a)	Protein name (b)	Molecular function/Biological process	Differential abundancy in TIF (c)			Differential expression in tumor biopsy (e)			Correlation TIF/Tumor (f)	Expression in normal breast tissue (close vicinity) (g)	Far‐distant	Presence in pooled NIF/FIF	Presence in exosome database [40]	Presence in Plasma database [39, 118]	Secreted (BC cell lines) [42]	Presence in serum (PubMed)	Expression in 33 normal human tissues (h)	Expression in BC biopsies (PubMed)	Expression in other cancers (i)
			Luminal	Her2	TNBC	Luminal	Her2	TNBC											
AGR3	Anterior gradient protein 3	Dystroglycan binding; negative regulation of cell death	↑ (d)	Not significant	↓	High (3+)	Medium/Low	Low (1+ – 0)	Yes	Low/Neg	Low/Neg	No	Yes	Yes	Yes	Yes [88]	Neg but medium in bladder, stomach, small intestine, colon and rectum	mRNA [139]; protein [88, 89, 109, 146]	Liver (protein [119, 129]), ovarian (mRNA [144]; protein [141]), prostate (mRNA [122])
BCAM	Lutheran/basal cell‐adhesion molecule, Lu/Bcam/CD239	Transmembrane signaling; cell adhesion and migration	↑	↓	↓	High (3+)	Medium/Low	Low (1+ – 0)	Yes	Low/Neg	Low/Neg	No	Yes	Yes	Yes	Yes [90]	Neg but medium in bladder, fallopian tube, esophagus, heart, kidney, lung, parathyroid, prostate and thyroid	Protein [90, 91]	Colon (protein [134]), hepatocellular carcinoma (plasma [130]; protein [113]), ovarian (protein [110]), pancreatic cancer (serum [115]), skin (protein [104, 106])
CELSR1	Cadherin EGF LAG seven‐pass G‐type receptor 1	Transmembrane signaling; cell adhesion and migration	↑	(↓)	↓	High (3+)	Low (1+ – 0)	Low (1+ – 0)	Yes	Low/Neg	Low/Neg	No	No	No	Yes	No Data	Neg in all	Gene copy [99, 121]	Hepatocellular carcinoma (DNA methylation [120]), glioma (mRNA [142]), lymphocytic leukemia (protein [127])
MIEN1	membrane‐anchored protein C35, C17orf37	Regulation of cell migration and apoptosis	↓	↑	↓	Medium (2+)	High (3+)	Low (1+ – 0)	Yes	Low/Neg	Low/Neg	No	Yes	Yes	Yes	No Data	Neg in all	Protein [76, 93, 94, 111]	Ovarian (mRNA [128]), oral (mRNA & protein [132])
NAT1	*N*‐acetyltransferase 1	Drugs and carcinogens metabolizing enzyme	↑	Not significant	↓	High (3+)	Low (1+ – 0)	Low (1+‐ 0)	Yes	Low/Neg	Low/Neg	No	Yes	No	No	No Data	Neg in all but medium in bladder	mRNA [25, 105, 107, 108]; protein [92, 112, 126]	No data
PIP4K2B	PhosphatidyliNositol‐5‐phosphate (PtdIns5P)‐4‐kinase	Stress‐regulated lipid kinase; cell surface receptor signaling pathway	Not significant	↑	↓	Medium (2+)	High (3+)	Low (1+ – 0)	Yes	Comparable	Comparable	No	Yes	Yes	No	No Data	Neg or very low in all	mRNA & protein [85, 125]	Lung adenocarcinoma (mRNA [138]), myeloid leukemia (mRNA [145])
SEC23B	Sec23 Homolog A	GTPase activator activity; cellular transport	Not significant	↑	↓	Medium (2+)	High (3+)	Low (1+ – 0)	Yes	Low/Neg	Low/Neg	No	Yes	No	Yes	No Data	Neg in all but medium in eye	mRNA & protein [133]	Thyroid & endometrial (protein [133])
THTPA	Thiamine triphosphatase	Hydrolase activity; metabolic and energy processes	↑	Not significant	↓	High (3+)	Low (1+ – 0)	Low (1+ – 0)	Yes	Low/Neg	Low/Neg	No	No	No	No	No Data	Neg or very low in all	No data	No data
TMEM51	Transmembrane protein	Unknown function	↑	↓	↑	Medium (2+)	Low (1+ – 0)	High (3+)	Yes	Low	Low	No	Yes	No	No	No Data	Neg in all but medium in bladder, kidney and pancreas	No data	Pancreatic (mRNA [136])
ULBP2	UL16‐binding protein 2	Cell surface glycoprotein; natural killer cell activation; immune response	↓	Not significant	↑	Low (1+ – 0)	Low (1+ – 0)	High (3+)	Yes	Low/Neg	Low/Neg	No	No	No	Yes	Yes [124, 131]	Neg in all but medium in heart	mRNA [143]; protein [96]	Colon (mRNA [140]), lung (plasma [123]), ovarian (mRNA [116]; protein [114], pancreatic (protein [135, 137]; plasma [117])

A key advantage of IHC analysis is that it provides visualization of spatial tissue architecture, including inter‐ and intracellular expression context. Upon the first examination of the IHC images obtained, we observed that most of the proteins exhibited expression patterns in both the cytoplasm and membrane, which is consistent with available literature. PIP4K2B exhibited strong nucleic positivity in several samples in addition to classical cytoplasmic and apical membrane staining, and this result is also consistent with previously published data [83]. It has been hypothesized that differential intracellular localization of PIP4K2B may be associated with the cellular functions of particular PI5P4K isoforms in different tumor subtypes [84]. With the exception of PI5P4K, all of the proteins exhibited significantly higher expression levels in malignant cells compared with normal cells (see the inset panels within the corresponding IHC panels of Fig. 8 and data presented in Table 1). The expression of PIP4K3B in normal mammary epithelium is consistent with data published by Keune *et al*. [85]. Interestingly, we also observed that the expression levels of all 10 proteins were significantly lower in the TILs located inside corresponding tumor lesions, regardless of subtype. In addition, positivity was not detected for any of the 10 proteins in adipose cells, nor in infiltrated or distant lesions (results not shown). Thus, collectively, our IHC data are in agreement with our comparative MS‐based analysis [see Identifying a minimal subset of disease‐related proteins: A consensus approach (DA analysis, LASSO regression, and random forest)], which did not detect any of these proteins in the NIF or FIF pooled samples. Taken together, these results clearly indicate that the abundance of our selected 10‐protein panel in the tumor interstitium is predominantly due to externalization of these proteins from neoplastic cells rather than from nonmalignant ductal epithelium, adipose cells, or the immuno‐complement of the tumor microenvironment.

Next, we compared the abundance of these 10 proteins across tumor tissues from different BC subtypes in order to validate a presumed correlation between intratumor expression levels and abundance within TIF. Due to the small sample size of the Her2 group, we were only able to apply Fisher's exact test of IHC score distribution to the luminal versus TNBC samples. Significant *P*‐values were obtained for all of the proteins except MIEN1 and TMEM51. This is in full accordance with the differential abundance/expression of MIEN1 and TMEM51 associated with the Her2 subtype, and these samples were not included in the test (Table 1 and Table S11). These results also support the TIF‐MS data in terms of differential expression/abundance levels of particular proteins detected with the paired tumor comparison of BC subtypes. To visualize the correlation between TIF and intratumor protein abundance, we generated tile plots of (a) batch‐corrected, MS‐based protein abundances of the top 10 candidates for discriminating BC subtypes and (b) IHC scores of the same 10 proteins (Table S11). The patterns of protein abundance/expression for the two plots (Fig. 8, panel B) were highly comparable, and importantly, a strong association was observed for Her2 samples, which were not subjected to Fisher's exact test. To better assess the correlation observed between the two tile plots, we performed logistic regression using the discrete IHC scores from solid tissues as the response and the normalized TIF protein abundances from LC‐MS/MS as the predictor. Results are visualized in Fig. 8 (panel C). The plot shows the probability of a given IHC score in response to the normalized protein abundance in TIF. All logistic regression models (one for each of the 10 proteins of interest) had an overall significant *P*‐value, indicating that TIF protein level was indeed predictive of IHC score, see Table S12. However, as was evident from the group‐specific *P*‐values in Table S12, TIF protein abundances were not found to have a significant effect on all levels of IHC for all proteins. In the case of ARG3, NAT1, and THTPA, there was a good correlation between the level of protein in TIF and all IHC scores, supported by the distinct probability peaks in Fig. 8 (panel C). In contrast, proteins BCAM and CELSR1 displayed less clear‐cut patterns, specifically with respect to the intermediate IHC score of 1–2, in accordance with accompanying *P*‐values.

For a molecule to be considered a serological cancer‐specific biomarker, it should be secreted predominantly from malignant tissues, not normal tissues. Therefore, we also analyzed expression levels of the selected candidate proteins in normal human tissues, using a TMA of normal human tissues deriving from 33 different organs (Pantomics, Inc., San Francisco, CA, USA), which is recommended by the FDA in its guidelines for testing cross‐reactivity. The results obtained are summarized in Table S13. We supplemented these data with publicly available information regarding the expression patterns of our candidates in normal tissues from: (a) the Protein Atlas (https://www.proteinatlas.org/), (b) the Integrated Proteomics Database (https://www.proteomicsdb.org/proteomicsdb/#overview), and (c) the Tissues Expression Database (https://tissues.jensenlab.org/Search). The latter is often used as a reference for protein expression in literature. We observed good consensus between the data available in these databases and our IHC results. For example, CELSR1, MIEN1, NAT1, PIP4K2B, SEC23B, THPPA, and ULBP1 were only detected at background levels in almost all of the normal tissues analyzed. Meanwhile, AGR3, BCAM, and TMEM51 exhibited noticeable expression levels in a number of normal tissues.

It is interesting to note that the minimal TIF protein set proposed in our study does not overlap with the protein signature of breast cancer subtypes described in the comprehensive proteome dataset of BC tissue biopsies published by Tyanova *et al*. [26]. This discrepancy may be due to significant differences in the methods used to obtain protein lysates for subsequent MS/MS analysis in these two studies. In contrast to our work in which fresh tissue was used, Tyanova *et al*. extracted proteins from FFPE tissue blocks with deparaffinization in xylene and ethanol. It cannot be ruled out that such differences in procedure may have affected the protein profiles of the samples examined. In addition, different statistical algorithms used in the two studies may have resulted in differences in the corresponding hits.

Therefore, to further validate the breast cancer subtype‐specific expression pattern of the 10 TIF proteins and to evaluate their potential clinical applicability as candidates for classification of BC subtype, we examined their sensitivity and specificity on Tyanova's proteome dataset. An AUC analysis was performed for eight of the 10 proteins (i.e., AGR3, BCAM, CELSR1, MIEN1, NAT1, PIP4K2B, SEC23B, and THTPA) because TMEM51 and ULBP2 had almost exclusively missing values, and thus, could not be included in the analysis. In light of more recent demonstrations that use of multiple markers can significantly increase the specificity and sensitivity of disease classification compared to the use of single biomarkers [86, 87], we estimated AUCs for both individual proteins and various protein combinations. Briefly, AUCs were estimated for: (a) individual AUCs for proteins DA within pairwise subtype contrasts, (b) combined AUC for multiple proteins associated with pairwise subtype contrasts, and (c) combined AUC for all proteins with all three BC subtypes included. The results obtained are summarized in Table 2.

**Table 2:** Validation of BC subtype‐specific expression profiles of selected protein biomarkers. AUC was estimated by using protein abundances quantified from 40 BC specimens (subtypes: luminal, Her2, and TNBC) by Tyanova *et al*. [26]. AUCs were estimated from: (a) models with individual markers used as classifiers of BC subtypes (pairwise), (b) a generalized linear model with additive combinations of markers in BC subtypes (pairwise), and (c) a multinomial log‐linear model fit via a neural network (Venables and Ripley [56]) by using a train/test set‐up with additive combinations of markers in BC subtypes (all). Parentheses around a protein name indicate that the corresponding AUC only increased minimally, or not at all when this protein was included in the model. A period indicates that the marker was not relevant for a given pairwise comparison. CI = 95% confidence interval was relevant/possible.

(a) Individual AUC scores with 95% confidence intervals for each protein associated with a pairwise subtype contrast								
	AGR3	BCAM	CELSR1	MIEN1	NAT1	PIP4K2B	SEC23B	THTPA
Luminal vs TNBC	0.85 (CI: 0.67–1)	0.80 (CI: 0.59–1)	0.77 (CI: 0.56–0.98)	.	0.84 (CI: 0.65–1)	.	.	0.77 (CI: 0.56–0.97)
Luminal vs Her2	.	0.87 (CI: 0.72–1.0)	.	0.83 (CI: 0.67–0.1.0)	.	.	.	.
Her2 vs TNBC	.	.	.	1.0 (CI: NA)	.	0.91 (CI: 0.8–1.0)	0.74 (CI: 0.55–0.93)	.
(b) AUC score with 95% confidence interval for combined proteins associated with a pairwise subtype contrast								
Luminal vs TNBC	**ARG3 + BCAM (+ CELSR1 + NAT1 + THTPA)**							
	0.92 (CI: 0.8–1.0)							
Luminal vs Her2	**BCAM + MIEN1**							
	0.94 (CI: 0.87–1.0)							
Her2 vs TNBC	**PIP4K2B (+ SEC23B)**				**MIEN1 + PIP4K2B (+ SEC23B)**			
	0.91 (CI: 0.8–1.0)				1.0 (CI: NA)			
(c) AUC scores (min. observed – max. observed) for the combination of all markers and three subtypes together. AUCs from a train/test multinomial log‐linear model, fit via neural network (cite)								
**Luminal vs Her2 vs TNBC**	**ARG3 + BCAM + MIEN1 + PIP4K2B (+ CELSR1 + NAT1 + SEC23B + THTPA)**							
	*0.85–0.91 (CI:NA)*							

The AUCs of the individual proteins ranged from 0.74 to 0.91, with SEC23B and PIPI4K2B having the lowest and highest specificity/sensitivity values, respectively, for classification of Her2 versus TNBC subtypes. Strikingly, MIEN1 had AUC = 1.0, which we partly attribute to the abundance of this protein being completely distinct between Her2 samples and TNBC samples. However, since MIEN1 was also one of the proteins which had the largest number of missing values (~ 35%), it was difficult to determine an exact AUC for this protein. Moreover, although the specificity/sensitivity of MIEN1 is likely to be high, it is doubtful that it would reach 1.0 in a larger dataset. For pairwise subtype classification by using a combination of markers, the AUCs were good, with values > 0.9 obtained for all three models (Table 2). In the combined model with luminal versus TNBC and Her2 versus TNBC, we observed a redundancy of markers. Additionally, we observed that use of more than two markers did not increase AUC in any noticeable manner. This is shown in Table 2 where a dual‐protein combination which maximized AUC is shown first, and AUCs associated with the remaining proteins which are specific for various subtype comparisons (denoted in parentheses) are below. Lastly, the combined AUC estimate for classification in the multinomial set‐up with all three subtypes in one model ranged from 0.85 to 0.91 (Table 2). The difference in AUC depended on how the dataset was split into test and training sets (see Methods) and essentially reflected how a small dataset with a large variance is highly sensitive to division. Overall, the AUC scores strongly support our observation that eight of the proteins selected (AGR3, BCAM, CELSR1, MIEN1, NAT1, PIP4K2B, SEC23B, and THTPA) exhibit potential as biomarkers for stratification of BC subtypes. However, their possible validity as serological markers for breast malignancy should be investigated, using an independent serum proteome dataset from BC patients.

Unfortunately, we were not able to verify the presence of the identified protein panel directly in matched blood samples since the latter samples had been consumed in previous studies [17, 18]. We also could not find any publicly available database of plasma proteins classified by BC subtype and containing information about potential serological BC subtype‐related biomarkers. Therefore, we indirectly evaluated the validity of our 10‐protein set as a potential serological signature by curating relevant databases (Fig. 2). The information used included: (a) a breast TIF MS‐dataset acquired from a pilot experiment [31], (b) the most updated versions of plasma and exosome databases [39, 40], (c) a secretome dataset derived from BC cell lines [42], and (d) the presence of these proteins in human serum according to available literature. The results of these curations, in combination with relevant protein characteristics (i.e., differential abundance in TIF and correlation with expression levels in matched tumors), are summarized in Table 1. A PubMed search revealed that six out of the 10 subtype‐specific proteins identified in our study have previously been characterized as differentially expressed in breast tumors at the protein and/or mRNA levels, compared to nonmalignant counterparts. These include AGR3 [88, 89], BCAM [90, 91], NAT1 [92], MIEN1 [93, 94, 95], PIP4K2B [85], and ULBP2 [96]. It should be noted, however, that with a few exceptions, most of these studies analyzed protein/mRNA levels in tumors without regard to subtyping, intratumor context, or secretion status. It has been reported that AGR3 is associated with less aggressive breast tumors and better BC patient outcome [89]. However, a PubMed search of thiamine triphosphatase (THTPA), transmembrane protein 51 (TMEM51), and cadherin EGF LAG seven‐pass G‐type receptor 1 (CELSR1), did not return any relevant literature about their expression in breast tumors. Thus, to the best of our knowledge, the present results appear to provide a first indication of the value of these three proteins for tumor subtype stratification.

**Fig. 9:**
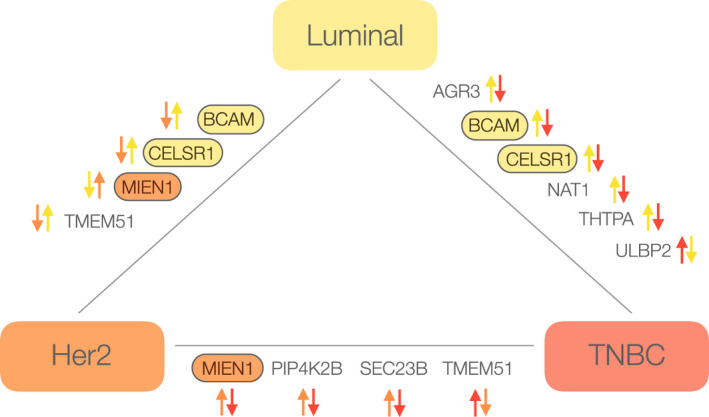
A schematic which represents the regulation pattern of 10 protein candidates in tumor and matched TIF samples according to luminal (yellow), Her2 (orange), and TNBC (red) tumor subtypes. Comparative directionality of protein abundance between subtypes is represented as arrows in corresponding colors. The proteins that are specifically upregulated in one subtype compared to the other two subtypes are marked with an oval filled with the corresponding color.

The expression profile of all 10 protein candidates, along with relative abundances in relation to the three main tumor subtypes, are schematically presented in Fig. 9. Six of the ten proteins (AGR3, BCAM, CELSR1, NAT1, THTPA, and TMEM51) were found to be significantly upregulated in the tumor/TIF of the luminal subtype samples. In particular, BCAM and CELSR1 are significantly elevated in the luminal subtype compared to the TNBC and Her2 subtypes (Fig. 9). Thus, these two proteins may represent a specific biomarker signature which can discriminate luminal subtype breast tumors.

BCAM, also known as CD239, is a plasma membrane glycoprotein and a receptor for the extracellular matrix protein, laminin [97]. Expression of CD239/BCAM is increased in invasive ductal carcinomas [91], and has also been found to be elevated level in a subset of BC tissues, particularly Her2‐negative tumors [91]. The present results are in agreement with these results. Furthermore, it has been hypothesized that BCAM represents a promising antigen for antibody‐drug conjugate‐based BC therapy [91]. BCAM is a secreted protein, and the significantly higher serum level of BCAM, determined by ELISA, has been reported in BC patients compared to normal individuals [90]. However, in the latter study, the tumors analyzed were not stratified, according to subtype.

Another member of our luminal‐specific signature is CELSR1, a protein shown to have a key role in epithelial planar cell polarity [98]. In general, very little is known about the potential role of this protein in carcinogenesis, and particularly in BC progression. A recently published study [99] demonstrated that CELSR1 is commonly amplified in pure, yet not mixed, ductal carcinoma *in situ* (DCIS) and is associated with invasion. Amplification of the 22q arm of chromosome 13, the position of *CELSR1*, is also frequently observed in DCIS [99]. In our IHC analysis, CELSR1 positivity was mainly associated with the cytoplasmic compartment, as expected for primary breast carcinomas. Moreover, CELSR1 positivity strongly correlated with less aggressive luminal type tumors. In contrast, TNBC tumors and normal ducts distant from the tumor site were almost exclusively negative for CELSR1 (Fig. 8, panel A).

Three proteins, MIEN1, PIP412B, SEC23B, were upregulated in tumor/TIF samples of the Her2‐enriched subtype compared to the levels detected in the luminal (MIEN1) and TNBC (MIEN1, PIP412B, and SEC23B) subtypes (Fig. 9). However, only the expression of MIEN1 was found to be specific to Her2 tumors (Fig. 9). MIEN1, migration and invasion enhancer 1, is a membrane‐anchored protein, which is highly expressed in various types of cancer. It was recently reported that expression of MIEN1 in human BC tissue is higher than in adjacent noncancerous breast tissue [93], which is consistent with our data. Notably, a direct correlation between upregulation of MIEN1 and upregulation of neighboring genes, ERBB2 and GRB7, was recently shown in a variety of cancers, including BC [93, 100, 101]. Meanwhile, *in vitro*, it has been demonstrated that overexpression of MIEN1 may promote cell dissemination and invasion in breast cancer by regulating cytoskeletal‐focal adhesion dynamics [102]. There is no information currently available regarding MIEN1 in human plasma. However, with curation of the Human Plasma PeptideAtlas [39], it has been confirmed that MIEN1 is present in circulation (Table 1). Thus, MIEN1 is a component of the breast tumor secretome in Her2‐positive patients, and targeting MIEN1 in the bloodstream may represent a promising approach to prevent breast tumor metastasis, especially for Her2‐enriched cancers.

In summary, our studies have led to the identification of three proteins, which have the potential to specifically discriminate between BC subtypes, particularly luminal (BCAM and CELSR1) and Her2 (MIEN1) enriched subtypes. The expression of TMEM51 was also found to be specific to the Her2 subtype (Fig. 9), although its downregulation in Her2 samples diminishes its value as a potential biomarker. Six additional proteins which we identified also exhibited expression levels relative to the BC subtypes examined, which manifested as pairwise differences.

## 4 Conclusions

Overall, by characterizing breast TIF proteome with high‐throughput LC‐MS/MS and bioinformatics analyses, we generated a database containing over 8800 proteins externalized from breast tumors into the tumor microenvironment,which represents the most comprehensive BC secretome dataset published to date. To maximize the probability of finding protein signature(s) associated with BC subtypes, ER/PgR/Her2 status and scoring of TILs we used a consensus bioinformatics approach including DAA, LASSO, and RF that led to the identification a minimal panel of 24 proteins, 10 of which namely, AGR3, BCAM, CELSR1, MIEN1, NAT1, PIP4K2B, SEC23B, THTPA, TMEM51, and ULBP2 were analyzed by IHC on matched tumor tissue samples, confirming their potential to stratify the BC tumor subtype‐specific TIFs. In particular, increased abundancy of BCAM and CELSR1 in TIF differentiates luminal, while upregulation of MIEN1 differentiates Her2 subtypes. The sensitivity and specificity were estimated for this 10‐protein panel in an independent, comprehensive breast tumor proteome dataset [26] using the AUC scores and the results strongly support our evidence that eight of the proteins (AGR3, BCAM, CELSR1, MIEN1, NAT1, PIP4K2B, SEC23B, and THTPA) might serve as biomarkers for stratification of luminal, Her 2 and TNBC tumor subtypes. Curation of the most relevant and current datasets of secreted and plasma proteins hypothesized the potential of identified proteins to serve as tumor‐specific biomarkers for plasma screening. Further studies are warranted to confirm the validity of these deregulated proteins as classifiers for particular breast tumor subtypes and to evaluate their value as potential serological biomarkers. We believe that the results presented in our study provide a system‐wide, quantitative baseline map and data resource of the breast interstitial fluid proteome, which extends the existing human tissue proteome databases.

## Conflict of interest

The authors declare no conflict of interest.

## Author contributions

IIG, EP, TT, and MP conceived and designed the project. MP acquired the data. IIG, EP, TT, MP, and PG analyzed and interpreted the data. IIG, EP, and TT wrote the manuscript. PSG and IIG collected the material and participated in the evaluation of data. All of the authors read and revised the manuscript critically and approved the final manuscript.

### Peer Review

The peer review history for this article is available at https://publons.com/publon/10.1002/1878‐0261.12850.

## Supporting information


**Fig. S1.** Figure showing the effects of batch correction on sample clustering and variance of protein abundance.

**Fig. S2.** Six set‐wise heatmaps (A‐F) with differentially abundant proteins from DAA comparisons.

**Table S1.** List of the antibodies used in this study.

**Table S2.** Clinicopathological characteristics of the breast cancer TIF samples which were examined in this study.

**Table S3.** Proteins returned from LASSO regression and Random Forest models.

**Table S4.** An alphabetical list of proteins and their detection profile in tumor, normal, and fat interstitial fluid samples (TIF, NIF, and FIF, respectively).

**Table S5.** Table with fractions (percentages) of BC samples within clusters belonging to each clinicopathological subgroup.

**Table S6.** An alphabetical list of 174 (176) differentially abundant TIF proteins according to limma analysis.

**Table S7.** The results of the differential abundance analysis with limma.

**Table S8.** Results from differential abundance analysis, LASSO regression, and random forest.

**Table S9.** Comparison of the expression profiles for eight of the TIF proteins identified in the present study with the PAM50 prognostic signature.

**Table S10.** Protein‐protein interaction networks from analysis using the STRING database.

**Table S11.** A panel of 10 proteins identified in TIF samples in the present study are segregated according to immunohistochemistry (IHC) scores from paired tumor tissues and BC subtype (Her2, luminal, TNBC).

**Table S12.** Test‐statistics and p‐values from ordinal logistic regression with ten protein candidates.

**Table S13.** Expression profiles for 10 selected proteins in 33 normal human tissues (MNO661; Pantomics, USA) according to immunohistochemistry (IHC) scores.

**Supplementary Material**
