# Supplementary figures and images for: High‐throughput proteomics of breast cancer interstitial fluid: identification of tumor subtype‐specific serologically relevant biomarkers
Source: Mol Oncol. 2021 Jan 4;15(2):429–61. doi: 10.1002/1878-0261.12850 (PMC7858121; doi:10.1002/1878-0261.12850)

A

Before Batch Correstion

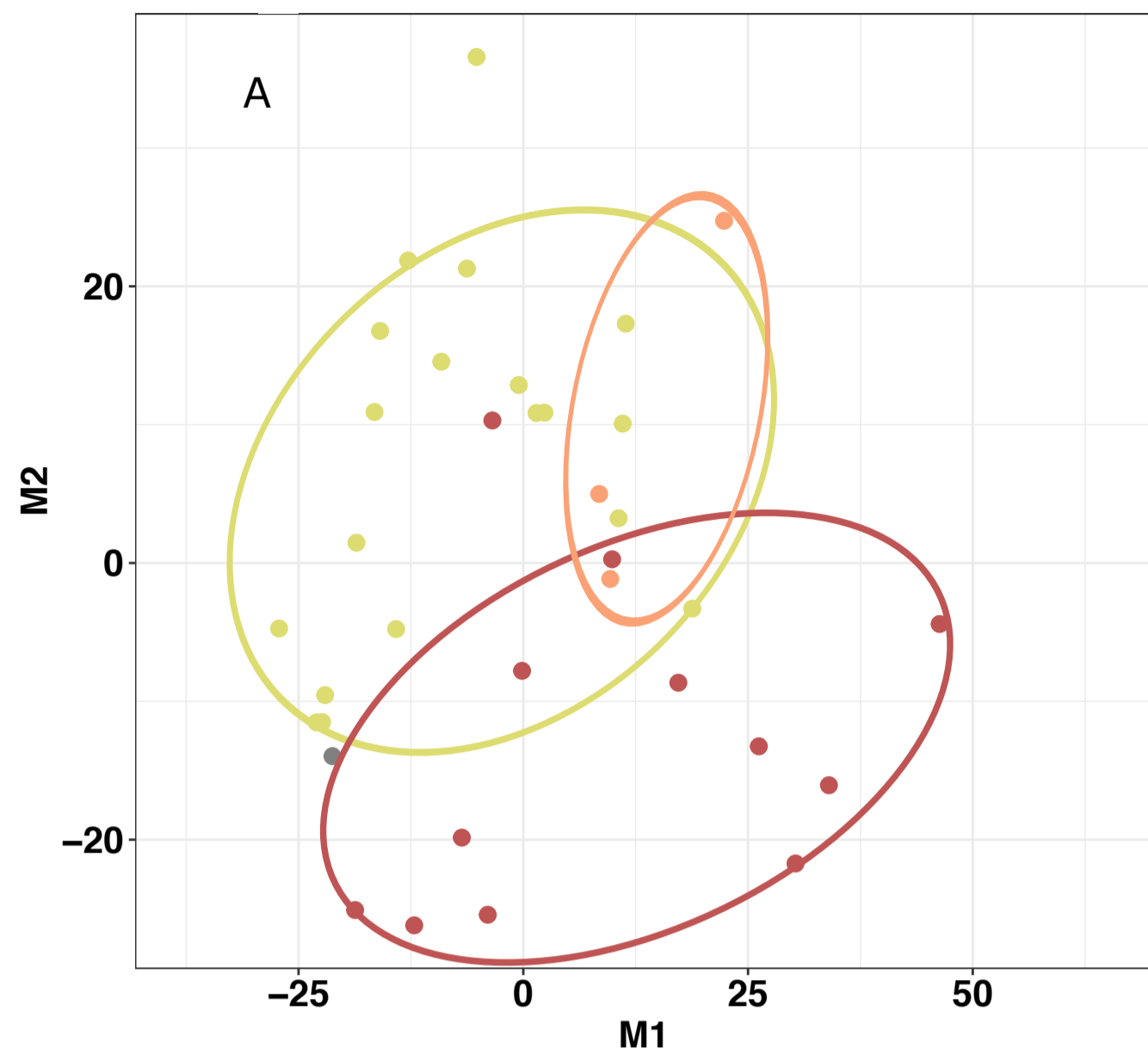

B

After Batch Correstion

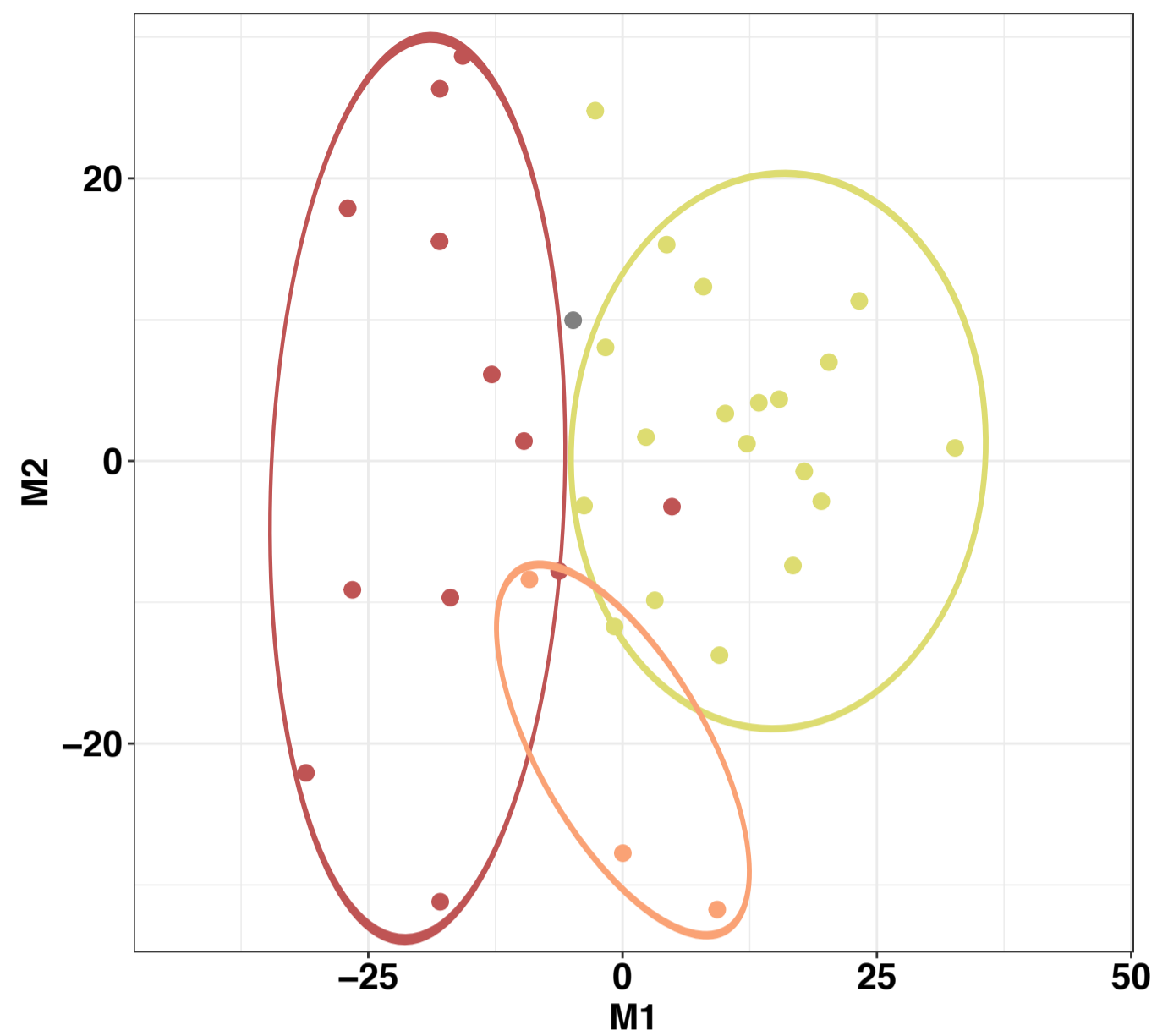

● Luminal  
● Her2  
● TNBC

C

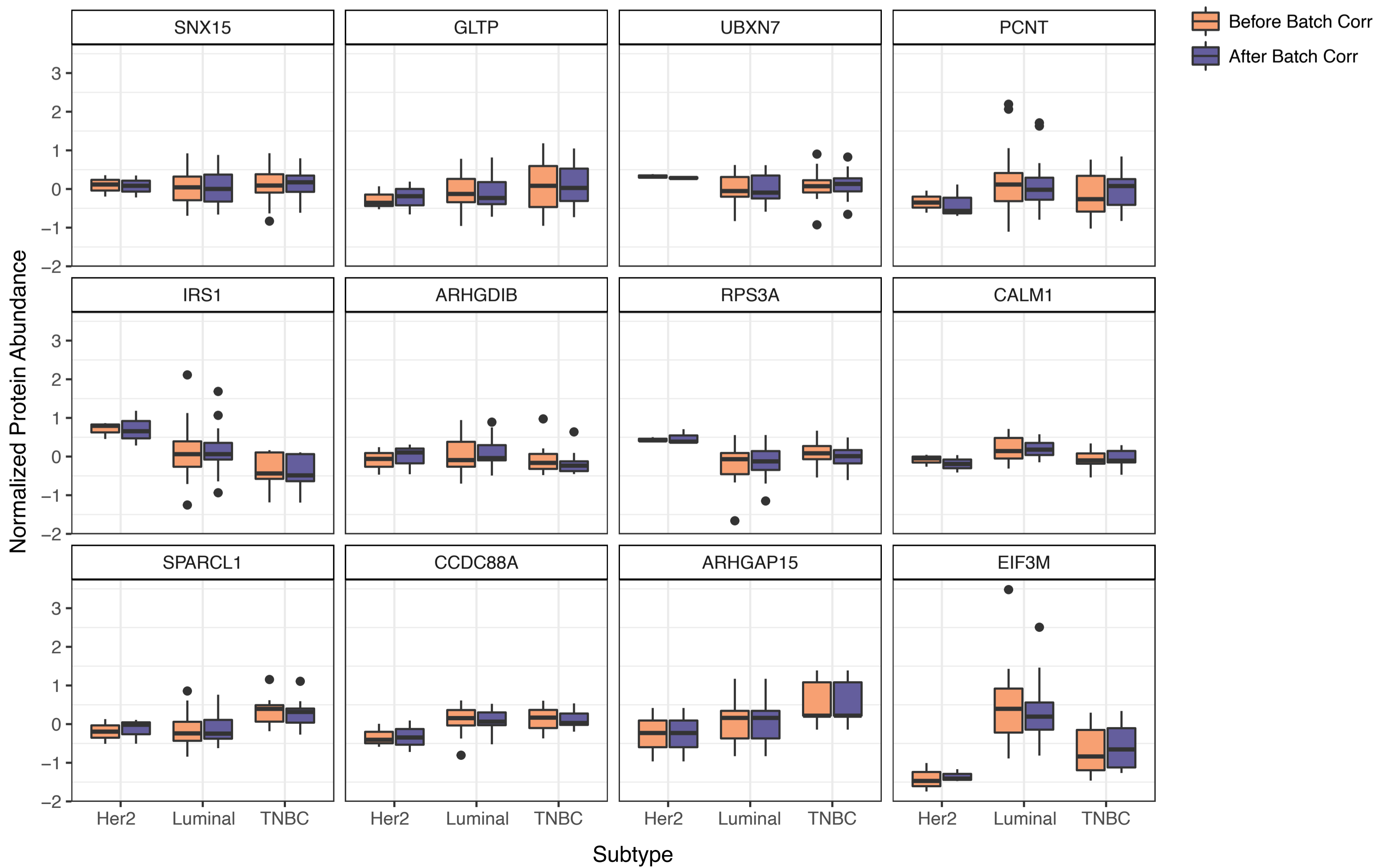

Supplement: Supplementary file 1 — Fig. S1. Figure showing the effects of batch correction on sample clustering and variance of protein abundance. [file MOL2-15-429-s001.pdf]

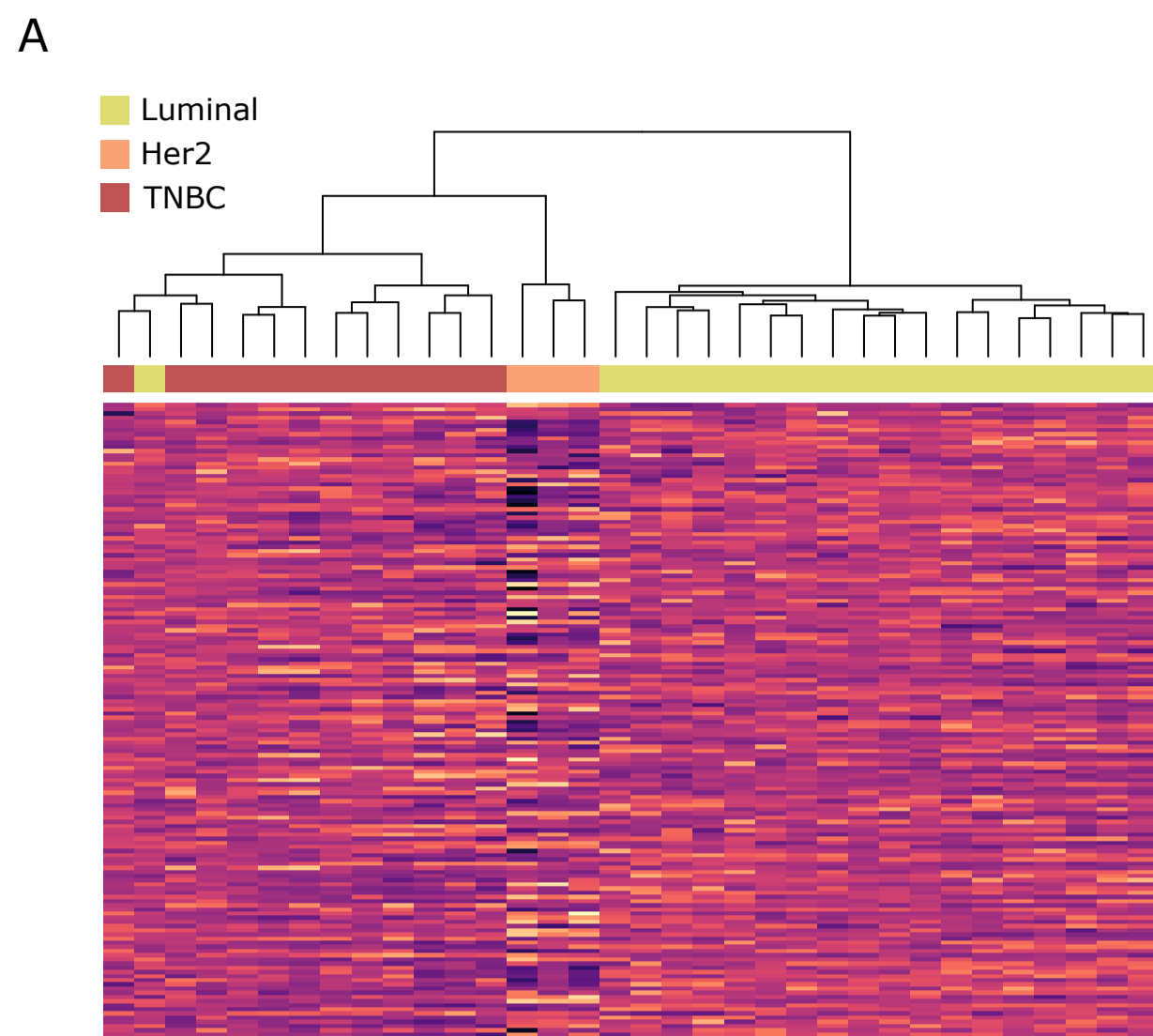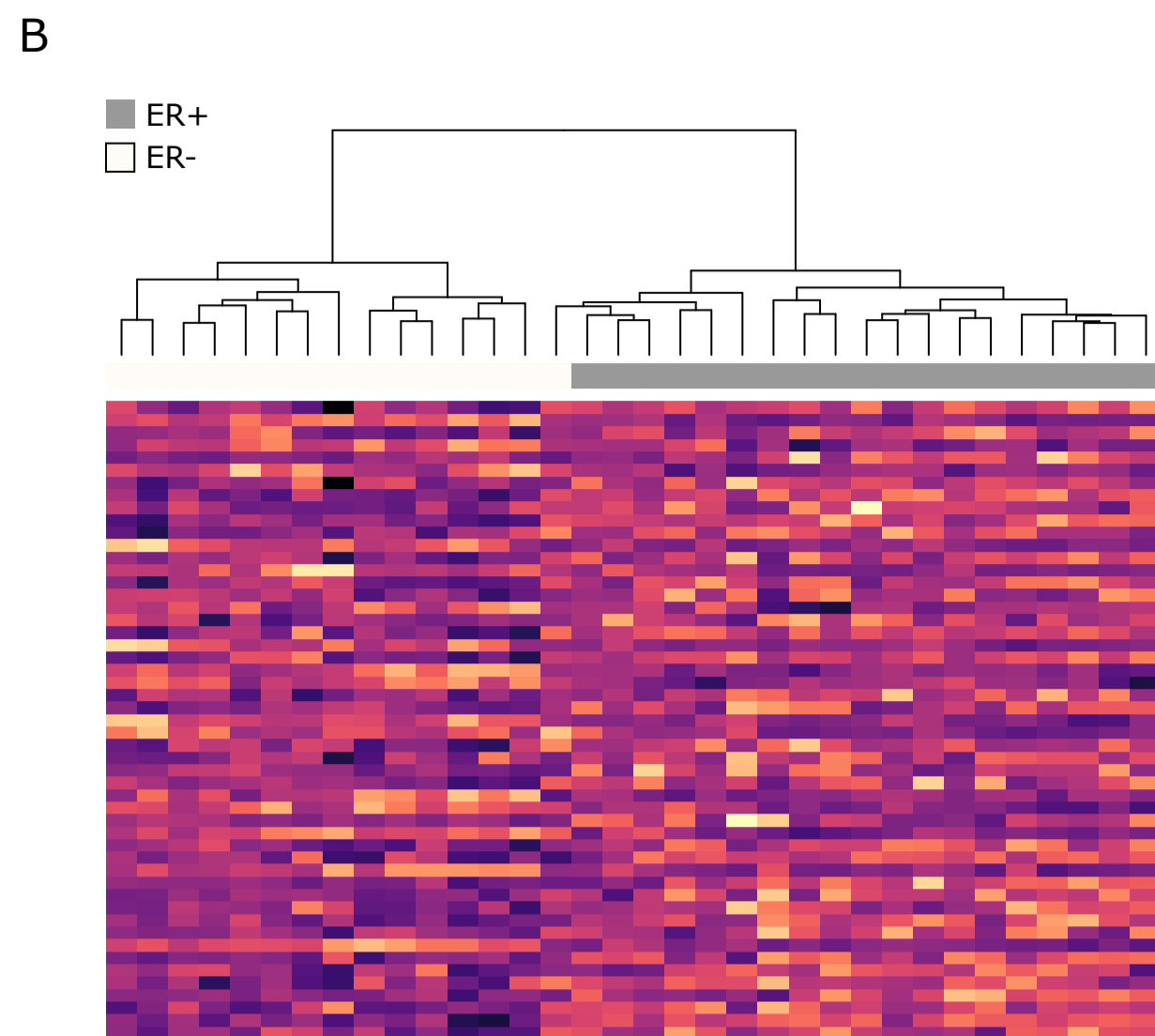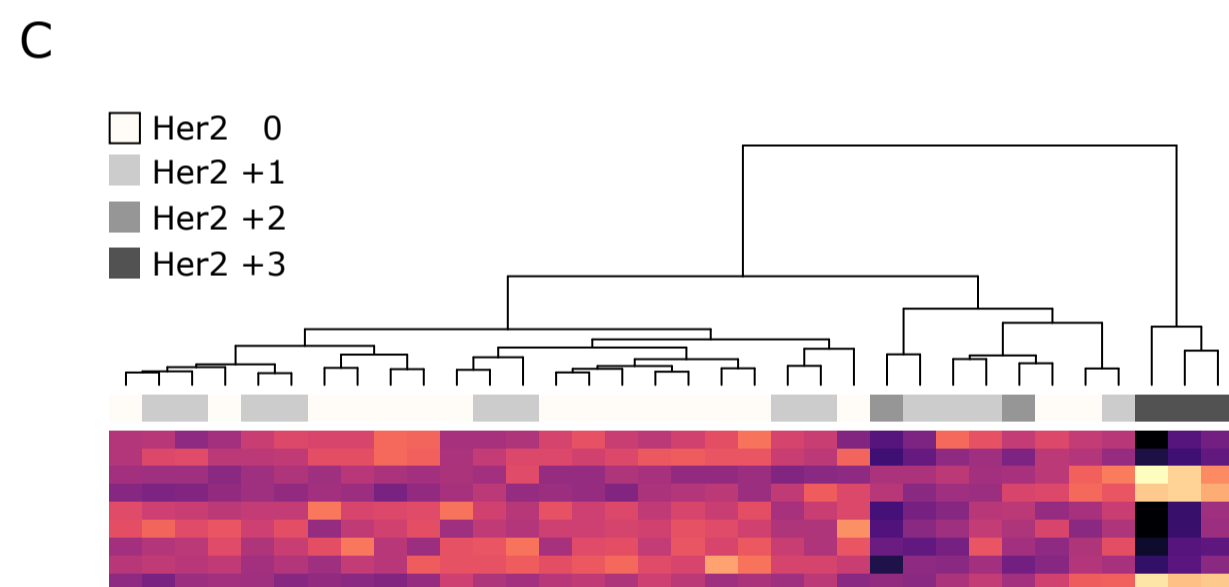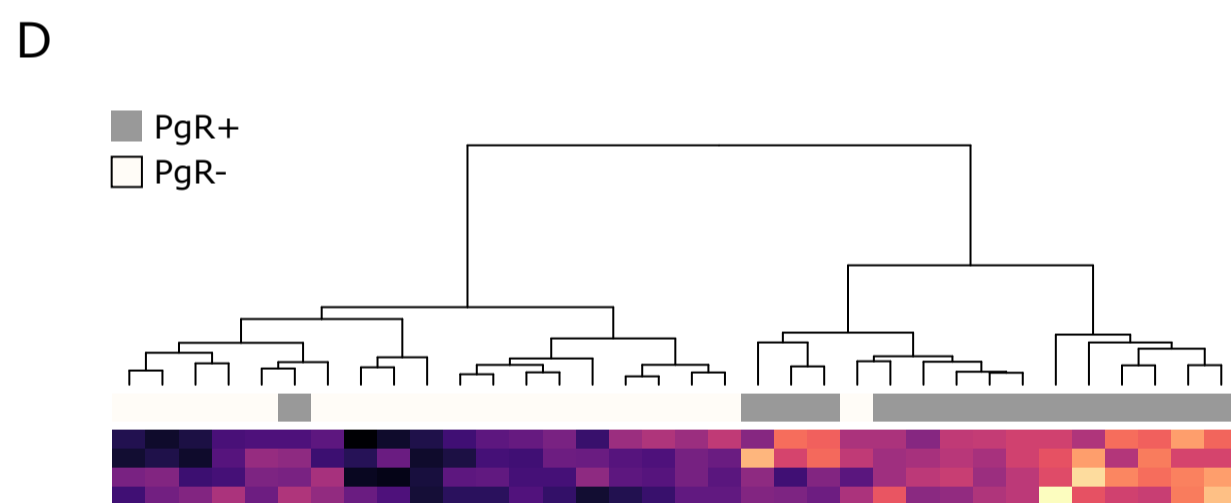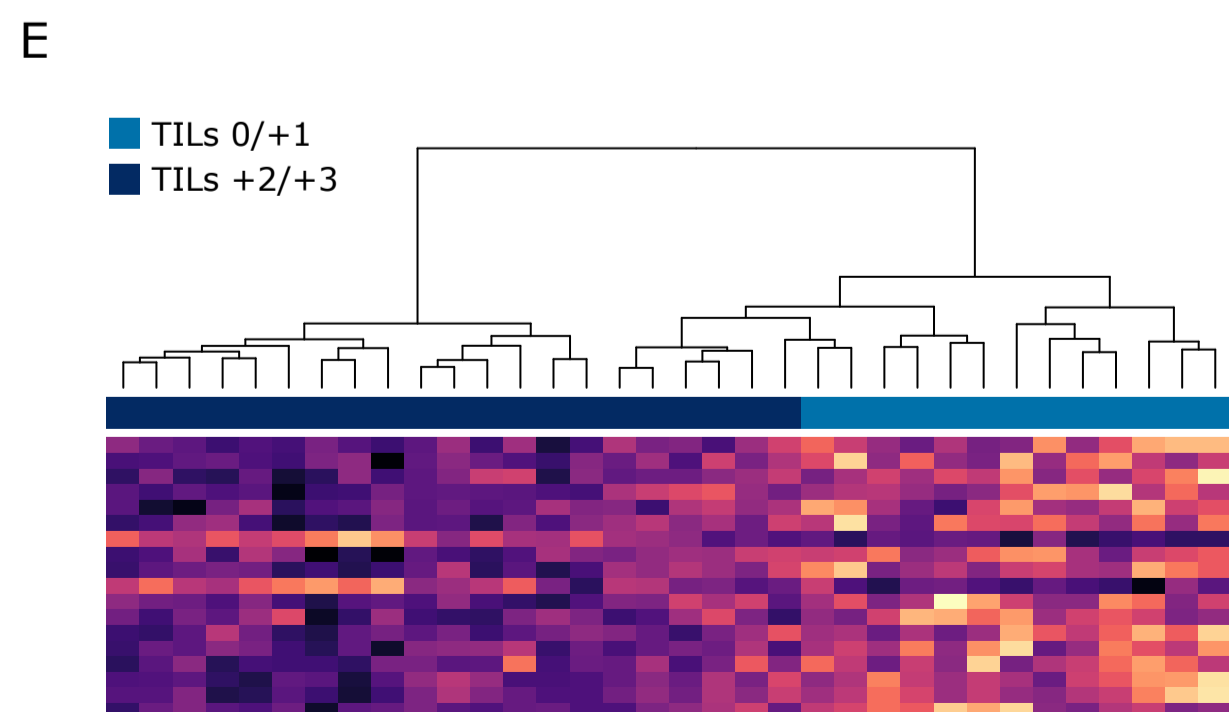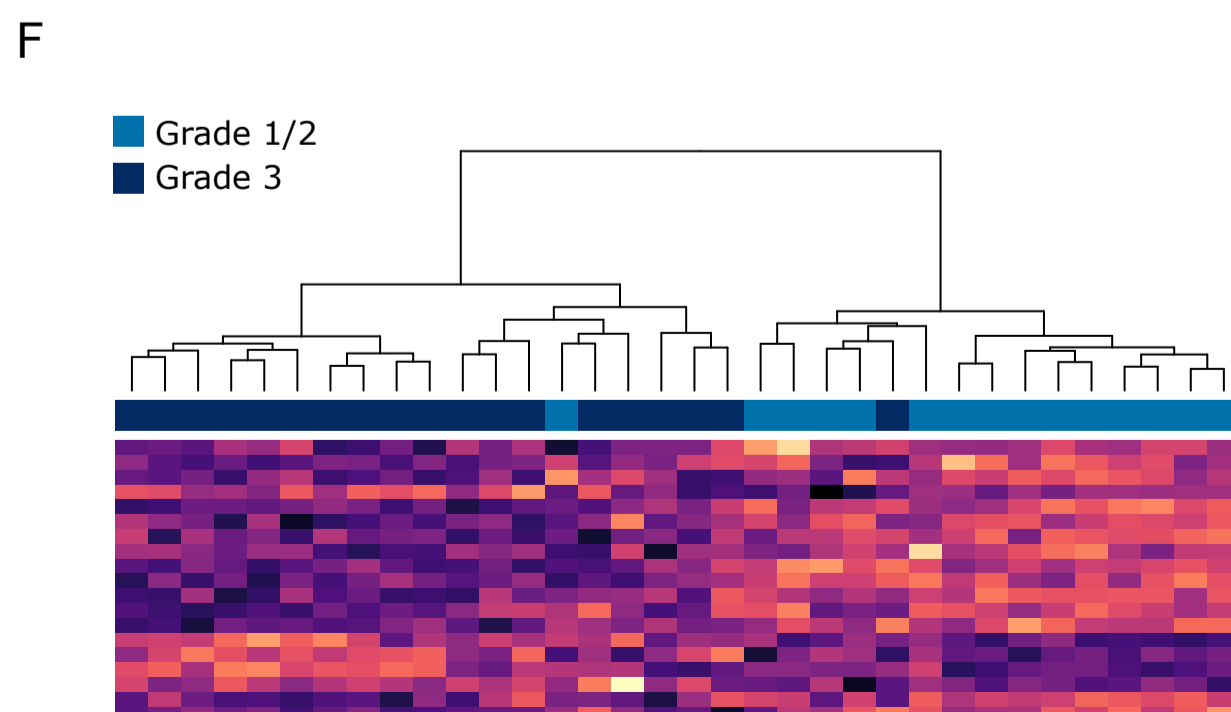

Supplement: Supplementary file 2 — Fig. S2. Six set‐wise heatmaps (A‐F) with differentially abundant proteins from DAA comparisons. [file MOL2-15-429-s002.pdf]
