# Supplementary material for: High‐throughput proteomics of breast cancer interstitial fluid: identification of tumor subtype‐specific serologically relevant biomarkers
Source: Mol Oncol. 2021 Jan 4;15(2):429–61. doi: 10.1002/1878-0261.12850 (PMC7858121; doi:10.1002/1878-0261.12850)
Supplement: Supplementary file 3 — Table S1. List of the antibodies used in this study. [file MOL2-15-429-s003.pdf]

Supplementary Table S1. *List of the antibodies used in this study.*

Antigen	Antibody	Specificity	Vendor	IHC dilution	Scoring
**ER**	Monoclonal mouse	clone 1D5	DAKO	1:200	Estrogen receptor (ER) status was assigned in accordance with the St. Gallen International Breast Cancer Guidelines (Esposito *et al*., 2015) as 1% cutoff
**PgR**	Monoclonal mouse	synthetic peptide directed against the *N* terminal end	DAKO	1:200	Progesteron receptor (PgR) status was assigned in accordance with the St. Gallen International Breast Cancer Guidelines (Esposito *et al*., 2015) as 1% cutoff
**Her2**	Polyclonal rabbit	HercepTest	DAKO	1:300	Her2-positivity was assigned if membrane positivity was 3+ and/or the fluorescence in situ hybridization (FISH) ratio of Her2 to CEP17 was 2.0. In case of IHC score of 2+ additional FISH was performed: value <2.0 was considered negative and a value > 2.0 was considered positive (Esposito *et al*., 2015)
**ki67**	Monoclonal mouse	clone MIB-1	DAKO	1:200	The cutoff for Ki67 positivity was assigned in accordance with currently accepted criteria (Esposito *et al*., 2015). Ki67 index values were measured using the open access web application, ImmunoRatio, which performs automated image analysis (Espinoza *et al*., 2016)
**CD45**	Monoclonal mouse	clone 2B11+PD7/26	DAKO	1:400	The stratification was performed as described previously (Espinoza *et al.,* 2016)
**AGR3**	Poyclonal rabbit	*N*-terminal fragment (1-132 aa) of human AGR3	Signalway Antibody (SAB)	1:500	The expression of AGR3 considered as positive if at least 10% of the tumor cells with the intensities of expression scored as (1-0+), medium (2) and high (3+) were detected as described (Obacz *et al.* 2015)
**BCAM**	Monoclonal rabbit	synthetic peptide directed towards the *C* terminal end	abcam	1:200	The same as above
**CELSR1**	Polyclonal rabbit	synthetic peptide corresponding to hCELSR1	Merck	1:200	The same as above
**MIEN**	Monoclonal mouse	full lenght recom. protein	Novusbio	1:200	The same as above and similar to (Katz *et al.,* 2010)
**NAT1**	Poyclonal rabbit	central part of recom. protein	GeneTex	1:400	The same as above and similar to (Savci-Heijink *et al.,* 2019)
**PIP4K2B**	Poyclonal rabbit	synthetic peptide directed against 286-317aa	LSBio	1:100	The same as above and similar to (Keune *et al*., 2013)
**SEC23B**	Poyclonal rabbit	synthetic peptide directed against the *N* terminal end	Sigma-Aldrich	1:800	The same as above
**THTPA**	Monoclonal mouse	full lenght recom. protein	Sigma-Aldrich	1:100	The same as above
**TMEM51**	Polyclonal rabbit	synthetic peptide directed against central part	Sigma-Aldrich	1:100	The same as above
**ULBP2**	Poyclonal rabbit		Cloud-Clone Corp	1:100	The same as above
