# Supplementary material for: High‐throughput proteomics of breast cancer interstitial fluid: identification of tumor subtype‐specific serologically relevant biomarkers
Source: Mol Oncol. 2021 Jan 4;15(2):429–61. doi: 10.1002/1878-0261.12850 (PMC7858121; doi:10.1002/1878-0261.12850)
Supplement: Supplementary file 4 — Table S2. Clinicopathological characteristics of the breast cancer TIF samples which were examined in this study. [file MOL2-15-429-s004.pdf]

Supplementary Table S2. *Clinicopathological characteristics of the breast cancer TIF samples examined in this study.*

**Variable**	**Levels**	**n**	**%**
**Age (in Years at Time of Surgery)**	30-40	3	8.6
	40-50	7	20
	50-60	4	11.4
	60-70	7	20
	70-80	7	20
	80-90	7	20
	all	35	100
**Tumor Type**	Apocrine	1	2.9
	Ductal	34	97.1
	all	35	100
**Tumor Size (in mm)**	<20mm	4	11.4
	20mm-30mm	14	40
	30mm-40mm	7	20
	40mm-50mm	4	11.4
	50mm-60mm	3	8.6
	>60mm	3	8.6
	all	35	100
**Tumor Grade**	1	3	8.6
	2	13	37.1
	3	19	54.3
	all	35	100
**Human Epidermal Growth Factor Receptor [illegible]**	Neg	17	48.6
	+1	12	34.3
	+2	2	5.7
	+3	4	11.4
	all	35	100
**Estrogen Receptor (ER)**	ER-	16	45.7
	ER+	19	54.3
	all	35	100
**Progesterone Receptor (PgR)**	PgR-	20	57.1
	PgR+	15	42.9
	all	35	100
**Tumor Infiltrating Lymphocytes (TILs)**	Neg	2	5.7
	+1	12	34.3
	+2	12	34.3
	+3	9	25.7
	all	35	100
**Breast Cancer Subtype**	Her2	4	11.4
	luminal	19	54.3
	TNBC	12	34.3
	all	35	100
