# Supplementary material for: High‐throughput proteomics of breast cancer interstitial fluid: identification of tumor subtype‐specific serologically relevant biomarkers
Source: Mol Oncol. 2021 Jan 4;15(2):429–61. doi: 10.1002/1878-0261.12850 (PMC7858121; doi:10.1002/1878-0261.12850)
Supplement: Supplementary file 5 — Table S3. Proteins returned from LASSO regression and Random Forest models. [file MOL2-15-429-s005.pdf]

Supplementary Table S3.1: Table containing proteins from LASSO models with BC subtypes (Luminal, Her2 and TNBC), estrogen receptor (ER), progesterone receptor (PgR), Her2 receptor (Her2), tumor-infitrating lymphocytes (TILs) and tumour grade. The table contains uniprot accession, gene name and weight. Proteins are ordered set wise according to weight.

	Accession	name	weight
**Subtypes**			
	Q9BRT3	MIEN1	0.33206
	Q7Z3D4	LYSMD3	0.33022
	Q9BU02	THTPA	0.28812
	O94992	HEXIM1	0.22099
	Q9H3Z4	DNAJC5	0.19785
	E7EV62	ARFGAP1	0.17757
	Q15437	SEC23B	0.14596
	Q9NZR1	TMOD2	0.09977
	Q9BVG4	PBDC1	0.09907
	P78356	PIP4K2B	0.09834
	P36405	ARL3	0.07718
	P50895	BCAM	0.07097
	H0YIQ2	YLPM1	0.06088
	Q9BSW2	CRACR2A	0.05163
	G5E9L0	ARFGAP2	0.03447
	Q9NW97	TMEM51	0.01103
	Q8TD06	AGR3	0.00739
	Q1MSJ5	CSPP1	0.00138
	Q9BZM5	ULBP2	0.00107
	P18440	NAT1	0.00096
**ER**			
	Q9BU02	THTPA	0.8570
	Q7Z3D4	LYSMD3	0.4888
	Q9HB07	C12orf10	0.4417
	G5E9L0	ARFGAP2	0.4145
	O94992	HEXIM1	0.2810
	Q969E4	TCEAL3	0.2592
	P43405	SYK	0.1607
	Q8TBC5	ZSCAN18	0.1099
	P50895	BCAM	0.1033
	Q8TD06	AGR3	0.0582
	Q9H7S9	ZNF703	0.0392
	O95049	TJP3	0.0035

**PgR**			
	P50895	BCAM	0.500
	E7EV62	ARFGAP1	0.442
	B7ZLZ0	GMIP	0.371
	H0YIQ2	YLPM1	0.140
	G3XAP6	COMP	0.090
	P35568	IRS1	0.083
	Q13576	IQGAP2	0.058
	Q8WXE0	CASKIN2	0.055
	O15394	NCAM2	0.050
**Her2**			
	Q9NRR5	UBQLN4	0.13269
	H0Y6K5	SP3	0.12145
	P04626	ERBB2	0.04903
	P17028	ZNF24	0.04753
	M0QZL8	JOSD2	0.04515
	Q05193	DNM1	0.03298
	H0Y6I0	GOLGA4	0.00965
	Q6DT37	CDC42BPG	0.00201
	Q92667	AKAP1	0.00024
**TILs**			
	B9A018	USP39	0.5790
	Q15393	SF3B3	0.1906
	Q14116	IL18	0.1372
	Q9BU02	THTPA	0.1063
	P04792	HSPB1	0.0836
	Q8NFP7	NUDT10	0.0686
	P25940	COL5A3	0.0676
	Q9BY32	ITPA	0.0602
	P10636	MAPT	0.0476
	Q8WVQ1	CANT1	0.0474
	Q9UJA5	TRMT6	0.0447
	Q96NL8	C8orf37	0.0441
	O43505	B4GAT1	0.0345
	P35052	GPC1	0.0336
	P16885	PLCG2	0.0305
	E7EVV3	SPATA18	0.0190
	B1ALD9	POSTN	0.0105
	P20645	M6PR	0.0047

Grade			
	Q9NX94	WBP1L	0.7805
	Q9UMY4	SNX12	0.6577
	Q9UPQ3	AGAP1	0.5837
	C9J2C3	GALNT3	0.3955
	Q9HB07	C12orf10	0.3847
	Q5T447	HECTD3	0.3245
	E9PFN4	SLC4A7	0.2247
	P08243	ASNS	0.2088
	Q92692	NECTIN2	0.2013
	D6RFH4	CYB5B	0.1526
	O94910	ADGRL1	0.1040
	Q5T013	HYI	0.0893
	P04066	FUCA1	0.0560
	Q96EY4	TMA16	0.0547
	O60701	UGDH	0.0140
	Q8TB36	GDAP1	0.0032

Supplementary Table S3.2: Table containing cross-validation errors and accuracies with confidence intervals from LASSO regression models.

	Cross-validation error	Accuracy	Accuracy Conf.Int.
**Subtype**	0.11	0.88	[0.49-0.99]
**ER**	0.07	0.89	[0.52-0.99]
**PgR**	0.09	0.88	[0.52-0.99]
**Her2**	0.13	NA	NA
**TILs**	0.29	NA	NA
**Grade**	0.24	NA	NA

Supplementary Table S3.3: Table containing proteins from Random Forest models with BC subtypes (Luminal, Her2 and TNBC), estrogen receptor (ER), progesterone receptor (PgR), Her2 receptor (Her2), tumor-infitrating lymphocytes (TILs) and tumour grade. The table contains uniprot accession, gene name and weight. Proteins are ordered set wise according to weight.

	Accession	name	weight
**Subtypes**			
	Q7Z3D4	LYSMD3	0.095
	E9PFK9	RABGEF1	0.092
	Q9BU02	THTPA	0.091
	Q9HB07	C12orf10	0.083
	J3KNL6	SEC16A	0.080
	Q969E4	TCEAL3	0.067
	Q9NYQ6	CELSR1	0.048
**ER**			
	Q9BU02	THTPA	0.133
	Q969E4	TCEAL3	0.119
	Q9HB07	C12orf10	0.106
	E9PFK9	RABGEF1	0.103
	J3KNL6	SEC16A	0.100
	Q9H7S9	ZNF703	0.075
	Q7Z3D4	LYSMD3	0.070
	Q9NYQ6	CELSR1	0.058
**PgR**			
	P50895	BCAM	0.127
	E9PG22	CEP97	0.102
	E9PGQ4	FNBP1	0.100
	B7ZLZ0	GMIP	0.088
	E7EV62	ARFGAP1	0.076
	Q9BU02	THTPA	0.058
	Q6UXD5	SEZ6L2	0.049
	Q12756	KIF1A	0.049
**Her2**			
	P17028	ZNF24	0.059
	H0Y6K5	SP3	0.056
	P08243	ASNS	0.055
	Q96QR8	PURB	0.050
	P42330	AKR1C3	0.049
	O15047	SETD1A	0.047
	Q92667	AKAP1	0.038
	C9J2C3	GALNT3	0.038
**TILs**			
	P35052	GPC1	0.117
	B9A018	USP39	0.090
	Q5JY65	CRNKL1	0.076
	J3KND6	DTWD2	0.017
**Grade**			
	Q5T013	HYI	0.089
	Q92692	NECTIN2	0.086
	Q9NW97	TMEM51	0.065
	Q8TB36	GDAP1	0.062
	B0QYP2	CBX7	0.044
	P20827	EFNA1	0.037

Supplementary Table S3.4: Table containing out-of-bag (OBB) errors, class errors and accuracies with confidence intervals from Random Forest models.

	Class error	Out-of-bag (OBB) error	Accuracy	Accuracy Conf.Int.
Subtype		0.17	0.94	[0.55-1.0]
	Luminal	0.03		
	TNBC	0.20		
ER		0.16	0.89	[0.52-1.0]
	ER+	0.07		
	ER-	0.27		
PgR		0.19	0.78	[0.40-0.97]
	PgR+	0.32		
	PgR-	0.07		
Her2		0.15	NA	NA
TILs		0.24	NA	NA
Grade		0.27	NA	NA
