# Supplementary material for: High‐throughput proteomics of breast cancer interstitial fluid: identification of tumor subtype‐specific serologically relevant biomarkers
Source: Mol Oncol. 2021 Jan 4;15(2):429–61. doi: 10.1002/1878-0261.12850 (PMC7858121; doi:10.1002/1878-0261.12850)
Supplement: Supplementary file 6 — Table S4. An alphabetical list of proteins and their detection profile in tumor, normal, and fat interstitial fluid samples (TIF, NIF, and FIF, respectively). [file MOL2-15-429-s006.pdf]

Supplementary Table S4: *An alphabetical list of proteins and their detection profile in tumor, normal, and fat interstitial fluid samples (TIF, NIF, and FIF, respectively).* [.] denotes that a protein was not in a given set. Only proteins identified in more than one set are included in this table.

Accession (SwissProt)	Gene Symbol	TIF	NIF	FIF
P04217	A1BG	Yes	Yes	Yes
P01023	A2M	Yes	Yes	Yes
P60709	ACTB	Yes	Yes	Yes
H9KV75	ACTN1	Yes	Yes	Yes
P00325	ADH1B	Yes	Yes	Yes
Q15847	ADIRF	Yes	Yes	Yes
P43652	AFM	Yes	Yes	Yes
P01019	AGT	Yes	Yes	Yes
Q09666	AHNAK	Yes	Yes	Yes
P02765	AHSG	Yes	Yes	Yes
P14550	AKR1A1	Yes	Yes	Yes
P02768	ALB	Yes	Yes	Yes
P00352	ALDH1A1	Yes	Yes	Yes
P02760	AMBP	Yes	Yes	Yes
P04083	ANXA1	Yes	Yes	Yes
P07355	ANXA2	Yes	Yes	Yes
P08758	ANXA5	Yes	Yes	Yes
P02743	APCS	Yes	Yes	Yes
P02647	APOA1	Yes	Yes	Yes
P02652	APOA2	Yes	Yes	Yes
P06727	APOA4	Yes	Yes	Yes
P04114	APOB	Yes	Yes	Yes
P02656	APOC3	Yes	Yes	Yes
P05090	APOD	Yes	Yes	Yes
P02649	APOE	Yes	Yes	Yes
P02749	APOH	Yes	Yes	Yes
P25311	AZGP1	Yes	Yes	Yes
P61769	B2M	Yes	Yes	Yes
P01024	C3	Yes	Yes	Yes
P0C0L5	C4B	Yes	Yes	Yes
P0C0L5	C4B_2	Yes	Yes	Yes
P04003	C4BPA	Yes	Yes	Yes
P13671	C6	Yes	Yes	Yes
P10643	C7	Yes	Yes	Yes
P07357	C8A	Yes	Yes	Yes
P02748	C9	Yes	Yes	Yes

P00915	CA1	Yes	Yes	Yes
P07451	CA3	Yes	Yes	Yes
P27797	CALR	Yes	Yes	Yes
P04040	CAT	Yes	Yes	Yes
O43866	CD5L	Yes	Yes	Yes
P00746	CFD	Yes	Yes	Yes
P08603	CFH	Yes	Yes	Yes
G3V1A4	CFL1	Yes	Yes	Yes
E9PHK0	CLEC3B	Yes	Yes	Yes
O00299	CLIC1	Yes	Yes	Yes
P00450	CP	Yes	Yes	Yes
P22792	CPN2	Yes	Yes	Yes
O95865	DDAH2	Yes	Yes	Yes
P68104	EEF1A1	Yes	Yes	Yes
P06733	ENO1	Yes	Yes	Yes
P00488	F13A1	Yes	Yes	Yes
P15090	FABP4	Yes	Yes	Yes
P49327	FASN	Yes	Yes	Yes
P02675	FGB	Yes	Yes	Yes
P02792	FTL	Yes	Yes	Yes
P04406	GAPDH	Yes	Yes	Yes
D6RF35	GC	Yes	Yes	Yes
P21695	GPD1	Yes	Yes	Yes
P06744	GPI	Yes	Yes	Yes
P09211	GSTP1	Yes	Yes	Yes
P69905	HBA1	Yes	Yes	Yes
P69905	HBA2	Yes	Yes	Yes
P68871	HBB	Yes	Yes	Yes
P02042	HBD	Yes	Yes	Yes
P00738	HP	Yes	Yes	Yes
P02790	HPX	Yes	Yes	Yes
P04196	HRG	Yes	Yes	Yes
P07900	HSP90AA1	Yes	Yes	Yes
P08238	HSP90AB1	Yes	Yes	Yes
P11021	HSPA5	Yes	Yes	Yes
P04792	HSPB1	Yes	Yes	Yes
P01876	IGHA1	Yes	Yes	Yes
P01877	IGHA2	Yes	Yes	Yes
P01880	IGHD	Yes	Yes	Yes

P01857	IGHG1	Yes	Yes	Yes
P01859	IGHG2	Yes	Yes	Yes
P01860	IGHG3	Yes	Yes	Yes
P01861	IGHG4	Yes	Yes	Yes
P01871	IGHM	Yes	Yes	Yes
P04220	IGHM	Yes	Yes	Yes
P23083	IGHV1-2	Yes	Yes	Yes
P23083	IGHV1-2	Yes	Yes	Yes
P01743	IGHV1-46	Yes	Yes	Yes
P01743	IGHV1-46	Yes	Yes	Yes
P01766	IGHV3-13	Yes	Yes	Yes
P01766	IGHV3-13	Yes	Yes	Yes
P01781	IGHV3-7	Yes	Yes	Yes
P01834	IGKC	Yes	Yes	Yes
P04430	IGKV1-16	Yes	Yes	Yes
P04430	IGKV1-16	Yes	Yes	Yes
P01598	IGKV1-5	Yes	Yes	Yes
P01611	IGKV1D-12	Yes	Yes	Yes
P01611	IGKV1D-12	Yes	Yes	Yes
P01605	IGKV1D-33	Yes	Yes	Yes
P01613	IGKV1D-33	Yes	Yes	Yes
P01617	IGKV2D-28	Yes	Yes	Yes
P04433	IGKV3-11	Yes	Yes	Yes
P04433	IGKV3-11	Yes	Yes	Yes
P01625	IGKV4-1	Yes	Yes	Yes
P0CG05	IGLC2	Yes	Yes	Yes
B9A064	IGLL5	Yes	Yes	Yes
P01714	IGLV3-19	Yes	Yes	Yes
P01714	IGLV3-19	Yes	Yes	Yes
P01717	IGLV3-25	Yes	Yes	Yes
P01717	IGLV3-25	Yes	Yes	Yes
P04264	KRT1	Yes	Yes	Yes
P13796	LCP1	Yes	Yes	Yes
P07195	LDHB	Yes	Yes	Yes
P09382	LGALS1	Yes	Yes	Yes
Q08380	LGALS3BP	Yes	Yes	Yes
P02750	LRG1	Yes	Yes	Yes
P51884	LUM	Yes	Yes	Yes
P29966	MARCKS	Yes	Yes	Yes

P35579	MYH9	Yes	Yes	Yes
P02763	ORM1	Yes	Yes	Yes
P19652	ORM2	Yes	Yes	Yes
P30086	PEBP1	Yes	Yes	Yes
P07737	PFN1	Yes	Yes	Yes
P18669	PGAM1	Yes	Yes	Yes
P00558	PGK1	Yes	Yes	Yes
Q96PD5	PGLYRP2	Yes	Yes	Yes
P36871	PGM1	Yes	Yes	Yes
P01833	PIGR	Yes	Yes	Yes
P12273	PIP	Yes	Yes	Yes
P14618	PKM	Yes	Yes	Yes
P00747	PLG	Yes	Yes	Yes
O60240	PLIN1	Yes	Yes	Yes
P62937	PPIA	Yes	Yes	Yes
Q06830	PRDX1	Yes	Yes	Yes
P32119	PRDX2	Yes	Yes	Yes
P07602	PSAP	Yes	Yes	Yes
Q5T123	Q5T123	Yes	Yes	Yes
Q5VY30	RBP4	Yes	Yes	Yes
C9J9K3	RPSA	Yes	Yes	Yes
P31949	S100A11	Yes	Yes	Yes
P26447	S100A4	Yes	Yes	Yes
P06702	S100A9	Yes	Yes	Yes
P35542	SAA4	Yes	Yes	Yes
O95969	SCGB1D2	Yes	Yes	Yes
Q13228	SELENBP1	Yes	Yes	Yes
P01009	SERPINA1	Yes	Yes	Yes
P01011	SERPINA3	Yes	Yes	Yes
P08185	SERPINA6	Yes	Yes	Yes
P01008	SERPINC1	Yes	Yes	Yes
P36955	SERPINF1	Yes	Yes	Yes
B4E1H2	SERPING1	Yes	Yes	Yes
P00441	SOD1	Yes	Yes	Yes
P08294	SOD3	Yes	Yes	Yes
Q01995	TAGLN	Yes	Yes	Yes
P37802	TAGLN2	Yes	Yes	Yes
P02787	TF	Yes	Yes	Yes
B4E022	TKTL1	Yes	Yes	Yes

P62328	TMSB4X	Yes	Yes	Yes
P67936	TPM4	Yes	Yes	Yes
P02766	TTR	Yes	Yes	Yes
P07437	TUBB	Yes	Yes	Yes
P68371	TUBB4B	Yes	Yes	Yes
P22314	UBA1	Yes	Yes	Yes
Q99536	VAT1	Yes	Yes	Yes
P08670	VIM	Yes	Yes	Yes
P04004	VTN	Yes	Yes	Yes
P61981	YWHAG	Yes	Yes	Yes
P27348	YWHAQ	Yes	Yes	Yes
P63104	YWHAZ	Yes	Yes	Yes
P80723	BASP1	Yes	Yes	.
P07360	C8G	Yes	Yes	.
B1AK87	CAPZB	Yes	Yes	.
P31944	CASP14	Yes	Yes	.
G3XAM2	CFI	Yes	Yes	.
P21291	CSRP1	Yes	Yes	.
P01034	CST3	Yes	Yes	.
P63241	EIF5A	Yes	Yes	.
P00734	F2	Yes	Yes	.
F8WAN4	FRMD4A	Yes	Yes	.
B4E351	IGFBP4	Yes	Yes	.
P06310	IGKV2-30	Yes	Yes	.
P06310	IGKV3-11	Yes	Yes	.
D6RD17	JCHAIN	Yes	Yes	.
P13645	KRT10	Yes	Yes	.
P08727	KRT19	Yes	Yes	.
P04259	KRT6B	Yes	Yes	.
P14174	MIF	Yes	Yes	.
P26038	MSN	Yes	Yes	.
Q5T6W5	Obsolete	Yes	Yes	.
P27169	PON1	Yes	Yes	.
F5H628	POSTN	Yes	Yes	.
P05386	RPLP1	Yes	Yes	.
P06703	S100A6	Yes	Yes	.
P63313	TMSB10	Yes	Yes	.
P10599	TXN	Yes	Yes	.
O43707	ACTN4	Yes	.	Yes

P11766	ADH5	Yes	.	Yes
Q15848	ADIPOQ	Yes	.	Yes
P42330	AKR1C3	Yes	.	Yes
P05091	ALDH2	Yes	.	Yes
P49189	ALDH9A1	Yes	.	Yes
P04075	ALDOA	Yes	.	Yes
P03950	ANG	Yes	.	Yes
P01031	C5	Yes	.	Yes
P00918	CA2	Yes	.	Yes
P22676	CALB2	Yes	.	Yes
P62158	CALM1	Yes	.	Yes
Q6NZI2	CAVIN1	Yes	.	Yes
O95810	CAVIN2	Yes	.	Yes
Q969G5	CAVIN3	Yes	.	Yes
P31146	CORO1A	Yes	.	Yes
Q14019	COTL1	Yes	.	Yes
P07108	DBI	Yes	.	Yes
P13639	EEF2	Yes	.	Yes
Q9NZN4	EHD2	Yes	.	Yes
Q01469	FABP5	Yes	.	Yes
P16930	FAH	Yes	.	Yes
Q04446	GBE1	Yes	.	Yes
P35754	GLRX	Yes	.	Yes
P15104	GLUL	Yes	.	Yes
P07203	GPX1	Yes	.	Yes
P06396	GSN	Yes	.	Yes
Q9Y5Z4	HEBP2	Yes	.	Yes
P08107	HSPA1A	Yes	.	Yes
O75874	IDH1	Yes	.	Yes
P01602	IGKV1-5	Yes	.	Yes
P11047	LAMC1	Yes	.	Yes
Q15691	MAPRE1	Yes	.	Yes
P43121	MCAM	Yes	.	Yes
P43121	MCAM	Yes	.	Yes
Q9NQR4	NIT2	Yes	.	Yes
G5EA52	Obsolete	Yes	.	Yes
P20774	OGN	Yes	.	Yes
Q99497	PARK7	Yes	.	Yes
Q9NRX4	PHPT1	Yes	.	Yes

Q96Q06	PLIN4	Yes	.	Yes
P30041	PRDX6	Yes	.	Yes
P31323	PRKAR2B	Yes	.	Yes
Q969G5	PRKCDBP	Yes	.	Yes
Q6NZI2	PTRF	Yes	.	Yes
P13489	RNH1	Yes	.	Yes
P23297	S100A1	Yes	.	Yes
O95810	SDPR	Yes	.	Yes
P35237	SERPINB6	Yes	.	Yes
O76070	SNCG	Yes	.	Yes
Q01082	SPTBN1	Yes	.	Yes
P61764	STXBP1	Yes	.	Yes
E9PGF5	TNS1	Yes	.	Yes
P13693	TPT1	Yes	.	Yes
P15374	UCHL3	Yes	.	Yes
P55072	VCP	Yes	.	Yes
P04275	VWF	Yes	.	Yes
Q02952-3	AKAP12 (isoform 3)	.	Yes	Yes
M0QZL1	BLVRB	.	Yes	Yes
Q5TEZ5	C6orf163	.	Yes	Yes
B8ZZL6	CAPG	.	Yes	Yes
B4DFK7	CBR1	.	Yes	Yes
M0R3H1	CD22	.	Yes	Yes
P00751	CFB	.	Yes	Yes
P10909-4	CLU (isoform 4)	.	Yes	Yes
O60888-3	CUTA (isoform 3)	.	Yes	Yes
F8VX58	DCN	.	Yes	Yes
P59665	DEFA1	.	Yes	Yes
P59665	DEFA1B	.	Yes	Yes
Q12805-2	EFEMP1 (isoform 2)	.	Yes	Yes
J3KS93	EIF4A1	.	Yes	Yes
U3KQT1	ESD	.	Yes	Yes
P00748	F12	.	Yes	Yes
P23142-4	FBLN1 (isoform 4)	.	Yes	Yes
P02671-2	FGA (isoform 2)	.	Yes	Yes
P02679-2	FGG (isoform 2)	.	Yes	Yes
Q13642-1	FHL1 (isoform 1)	.	Yes	Yes
Q5HY54	FLNA	.	Yes	Yes
P02751-12	FN1 (isoform 12)	.	Yes	Yes

Q96QV1	HHIP	.	Yes	Yes
Q5T7C4	HMGB1	.	Yes	Yes
P22626-2	HNRNPA2B1 (isoform 2)	.	Yes	Yes
D6RF44	HNRNPD	.	Yes	Yes
P11142-2	HSPA8 (isoform 2)	.	Yes	Yes
Q16270-2	IGFBP7 (isoform 2)	.	Yes	Yes
P01764	IGHV3-23	.	Yes	Yes
P01779	IGHV3-23	.	Yes	Yes
P01620	IGKV3-20	.	Yes	Yes
P01621	IGKV3-20	.	Yes	Yes
P01699	IGLV1-44	.	Yes	Yes
P01699	IGLV1-44	.	Yes	Yes
P04208	IGLV1-47	.	Yes	Yes
P80748	IGLV3-21	.	Yes	Yes
P80748	IGLV3-21	.	Yes	Yes
Q5T985	ITIH2	.	Yes	Yes
H7C0L5	ITIH4	.	Yes	Yes
Q92953	KCNB2	.	Yes	Yes
P01042-3	KNG1 (isoform 3)	.	Yes	Yes
K7EQQ3	KRT9	.	Yes	Yes
P02545-2	LMNA (isoform 2)	.	Yes	Yes
P02788-2	LTF (isoform 2)	.	Yes	Yes
P40925-2	MDH1 (isoform 2)	.	Yes	Yes
B4DSQ1	Microtubule-associated protein	.	Yes	Yes
F8VPF3	MYL6	.	Yes	Yes
F5H7E1	Obsolete	.	Yes	Yes
J3KNT0	Obsolete	.	Yes	Yes
Q5TCU6	Obsolete	.	Yes	Yes
I3NI03	P4HB	.	Yes	Yes
P12955-2	PEPD (isoform 2)	.	Yes	Yes
Q6UXB8-2	PI16 (isoform 2)	.	Yes	Yes
B1AH77	RAC2	.	Yes	Yes
H0Y579	RAD23B	.	Yes	Yes
P08697-2	SERPINF2 (isoform 2)	.	Yes	Yes
P61956-2	SUMO2 (isoform 2)	.	Yes	Yes
F2Z393	TALDO1	.	Yes	Yes
P60174-1	TPI1 (isoform 1)	.	Yes	Yes
P68363	TUBA1B	.	Yes	Yes
Q8NBS9-2	TXNDC5 (isoform 2)	.	Yes	Yes

J3QSA3	UBB	.	Yes	Yes
P18206-2	VCL (isoform 2)	.	Yes	Yes
Q14508-2	WFDC2 (isoform 2)	.	Yes	Yes
P62258-2	YWHAE (isoform 2)	.	Yes	Yes
