# Supplementary material for: High‐throughput proteomics of breast cancer interstitial fluid: identification of tumor subtype‐specific serologically relevant biomarkers
Source: Mol Oncol. 2021 Jan 4;15(2):429–61. doi: 10.1002/1878-0261.12850 (PMC7858121; doi:10.1002/1878-0261.12850)
Supplement: Supplementary file 7 — Table S5. Table with fractions (percentages) of BC samples within clusters belonging to each clinicopathological subgroup. [file MOL2-15-429-s007.pdf]

**Supplementary Table S5.** This table contains information about the fraction (percentage) of samples belonging to each clinicopathological subgroup within the two clusters (Cluster 1 and Cluster 2) observed in Figure 4. The table has two sub-tables, one containing percentages within clusters and one with percentages [illegible]

**Table S5.1**

| Clinical Variable | Cluster1 | | Cluster 2 | |
|---|---|---|---|---|
| | *n* | % | *n* | % |
| **ER+** | 13 | 93% | 6 | 30% |
| **ER-** | 1 | 7% | 14 | 70% |
| | | | | |
| **PgR+** | 10 | 71% | 5 | 25% |
| **PgR-** | 4 | 29% | 15 | 75% |
| | | | | |
| **Her2 0** | 11 | 79% | 6 | 30% |
| **Her2 +1** | 3 | 21% | 9 | 45% |
| **Her2 +2** | 0 | 0% | 2 | 10% |
| **Her2 +3** | 0 | 0% | 3 | 15% |
| | | | | |
| **TILs 0/+1** | 9 | 64% | 4 | 20% |
| **TILs +2/+3** | 5 | 36% | 16 | 80% |
| | | | | |
| **Grade 1/2** | 10 | 71% | 5 | 25% |
| **Grade 3** | 4 | 29% | 15 | 75% |
| | | | | |
| **Luminal** | 13 | 93% | 6 | 30% |
| **Her2** | 0 | 0% | 3 | 15% |
| **TNBC** | 1 | 7% | 11 | 55% |
| | | | | |
| **Total** | 14 | 100% | 20 | 100% |

**Table S5.2**

| | | Clinical Variable | | | | | | | | | | | | | | |
|---|---|---|---|---|---|---|---|---|---|---|---|---|---|---|---|---|
| | | ER+ | ER- | PgR+ | PgR- | Her2 0 | Her2 +1 | Her2 +2 | Her2 +3 | TILs 0/+1 | TILs +2/+3 | Grade 1/2 | Grade 3 | Luminal | Her2 | TNBC |
| **Cluster1** | ***n*** | 13 | 1 | 10 | 4 | 11 | 3 | 0 | 0 | 9 | 5 | 10 | 4 | 13 | 0 | 1 |
| | **%** | 68% | 7% | 67% | 27% | 65% | 25% | 0% | 0% | 69% | 24% | 67% | 21% | 68% | 0% | 6% |
| **Cluster 2** | ***n*** | 6 | 14 | 5 | 15 | 6 | 9 | 2 | 3 | 4 | 16 | 5 | 15 | 6 | 3 | 11 |
| | **%** | 32% | 93% | 33% | 73% | 35% | 75% | 100% | 100% | 31% | 76% | 33% | 79% | 32% | 100% | 92% |
| **Total** | ***n*** | 19 | 15 | 15 | 19 | 17 | 12 | 2 | 3 | 13 | 21 | 15 | 19 | 19 | 3 | 12 |
| | **%** | 100% | 100% | 100% | 100% | 100% | 100% | 100% | 100% | 100% | 100% | 100% | 100% | 100% | 100% | 100% |
