# Supplementary material for: High‐throughput proteomics of breast cancer interstitial fluid: identification of tumor subtype‐specific serologically relevant biomarkers
Source: Mol Oncol. 2021 Jan 4;15(2):429–61. doi: 10.1002/1878-0261.12850 (PMC7858121; doi:10.1002/1878-0261.12850)
Supplement: Supplementary file 8 — Table S6. An alphabetical list of 174 (176) differentially abundant TIF proteins according to limma analysis. [file MOL2-15-429-s008.pdf]

Supplementary Table S6. *An alphabetical list of 174 (176) differentially abundant TIF proteins according to limma analysis.* Increases or decreases in expression are presented according to BC subtype, hormone receptor status, and TIL level. It is also indicated whether each protein is detected (Y) or absent (.) in BC cell lines, in the human plasma proteome , in TIF samples from another cohort of BC patients, and/or is externalized via exosomes or plasma microvesicles. Criterion for significance was an adjusted p.value (FDR) < 0.05 and a log2 fold change of +1 (up-regulated) and -1 (down-regulated). Two of the genes, POSTN and SYTL2, have two different UniProt IDs assigned.

SwissProt	Gene symbol	BC Subtypes			ER+ vs ER-	PgR+ vs PgR-	Her2 High (+3/+2) vs Low (+1/0)	TILs High (+3/+2) vs Low (+1/0)	Secreted	Plasma	Exosomes	Plasma Microvesicles	TIF
		Her2 vs TNBC	Her2 vs Luminal	Luminal vs TNBC									
P80404	ABAT	.	.	up	up	.	.	.	.	Y	.	.	.
Q15847	ADIRF	.	.	up	.	.	.	.	Y	Y	Y	.	.
O95994	AGR2	.	.	up	up	.	.	.	Y	Y	Y	.	.
Q8TD06	AGR3	.	.	up	up	.	.	.	Y	Y	Y	.	.
Q9NQW6	ANLN	.	.	down	.	.	.	.	Y	.	Y	.	.
Q63HQ0	AP1AR	.	down	.	.	.	.	.	.	.	.	.	.
P53365	ARFIP2	.	.	up	up	.	.	.	Y	.	.	.	.
Q9H993	ARMT1	.	.	up	up	.	.	.	Y	Y	Y	.	.
P08243	ASNS	.	up	.	.	.	.	.	Y	.	Y	.	.
Q9ULI0	ATAD2B	.	up	.	.	.	.	.	.	.	Y	.	.
G3V3R7	ATXN3	.	down	.	.	.	.	.	.	.	.	.	.
P50895	BCAM	.	down	up	up	up	.	.	Y	Y	Y	Y	.
O75363	BCAS1	up	up	.	.	.	.	.	Y	.	.	.	.
O43570	CA12	.	.	up	up	.	.	.	Y	Y	Y	.	.
Q16790	CA9	.	.	down	down	.	.	.	.	.	Y	.	.
F8W8P5	CADPS2	up	.	.	.	.	.	.	.	.	.	.	.
P49913	CAMP	.	.	down	down	.	.	.	.	Y	.	Y	.
F5H2U1	CBS	.	.	down	.	.	.	.	.	.	.	.	.
Q8WUD4	CCDC12	.	down	.	.	.	.	.	Y	.	.	.	.
Q5VZ73	CCL21	.	up	.	.	.	.	.	.	.	.	.	.
Q9NYV4	CDK12	up	up	.	.	.	.	.	.	.	Y	.	.
Q14004	CDK13	.	down	.	.	.	.	.	.	Y	Y	Y	.
E7EQD6	CEGP1	.	.	.	.	.	.	down	.	.	.	.	.
Q9NYQ6	CELSR1	.	.	up	up	.	.	.	Y	.	.	.	.
E9PG22	CEP97	.	down	.	.	.	.	.	.	.	Y	.	.
Q9BXR6	CFHR5	.	.	down	.	.	.	.	.	Y	.	Y	.
Q96RK0	CIC	.	down	.	.	.	.	.	.	.	.	.	.
Q96DG6	CMBL	.	.	up	.	.	.	.	Y	Y	Y	.	.
Q13057	COASY	.	down	.	.	.	.	.	Y	Y	Y	Y	.
P83436	COG7	up	.	.	.	.	.	.	.	.	Y	.	Y
P38432	COIL	.	up	.	.	.	.	.	.	.	.	.	.
P25940	COL5A3	.	.	.	.	.	.	down	.	Y	.	.	.
G3XAP6	COMP	.	.	.	.	up	.	.	.	.	Y	.	.

J3QT29	COPS9	.	down	up	up	up	.	.	.	.	.	.	.
P54108	CRISP3	.	.	down	.	.	.	.	Y	Y	.	.	.
H0YDQ8	CRTC2	.	down	.	.	.	.	.	.	.	Y	.	.
Q14894	CRYM	up	up	.	.	.	.	.	.	Y	Y	.	.
B0QY35	CSNK1E	.	up	.	.	.	.	.	.	.	.	.	.
Q9NSA3	CTNNBIP1	.	down	.	.	.	.	.	.	.	.	.	.
B4DDP6	DBNL	.	down	.	.	.	.	.	Y	.	.	Y	.
E7EUD0	DKK3	.	.	.	.	.	.	down	.	.	.	.	.
Q9UKB3	DNJC12	.	down	up	up	.	.	.	.	.	.	.	.
G8JLD5	DNM1L	.	down	.	up	.	.	.	.	.	Y	Y	.
Q16610	ECM1	.	.	.	.	.	.	down	Y	Y	Y	Y	.
O94769	ECM2	.	down	.	.	.	.	.	.	Y	.	.	.
B1AM48	ELAVL2	.	.	down	.	.	.	.	.	.	.	.	.
P04626	ERBB2	up	up	.	.	.	up	.	Y	Y	Y	.	.
P21860	ERBB3	.	.	.	up	.	.	.	Y	Y	Y	.	.
B7Z4R0	ERF	.	down	.	.	.	.	.	.	.	.	.	.
Q9UI08	EVL	.	down	.	up	.	.	.	.	.	Y	.	.
O15540	FABP7	.	.	down	.	.	.	.	.	.	.	.	.
P37268	FDFT1	up	up	.	.	.	.	.	.	.	Y	.	.
Q6ZNL6	FGD5	.	up	.	.	.	.	.	.	.	.	.	.
Q9H479	FN3K	.	.	.	up	.	.	.	Y	Y	Y	Y	.
Q8WU20	FRS2	.	.	up	.	.	.	.	.	.	.	.	.
P19883	FST	.	.	.	.	.	.	down	Y	Y	Y	.	.
Q14353	GAMT	.	.	up	.	.	.	.	Y	Y	.	.	.
Q9H2C0	GAN	.	up	.	.	.	.	.	.	.	.	.	.
Q14C86	GAPVD1	.	down	.	.	.	.	.	Y	.	.	.	.
P23771	GATA3	.	.	up	up	.	.	.	.	.	.	.	.
Q9H4G4	GLIPR2	down	.	.	.	.	.	.	.	Y	Y	Y	.
P24298	GPT	.	.	down	.	.	.	.	Y	Y	Y	.	.
Q14451	GRB7	up	up	.	down	.	up	.	Y	.	.	.	.
P21266	GSTM3	.	.	up	.	.	.	.	Y	Y	Y	Y	.
Q5JVS0	HABP4	.	down	.	.	.	.	.	.	.	.	.	.
Q96MH2	HEXIM2	.	.	up	.	.	.	.	.	.	.	.	.
Q8IV36	HID1	.	.	up	up	.	.	.	Y	.	Y	.	.
Q01581	HMGCS1	up	up	.	.	.	.	.	Y	Y	Y	.	.
P04792	HSPB1	.	.	.	.	.	.	down	Y	Y	Y	Y	Y
P14735	IDE	up	.	.	.	.	.	.	Y	Y	Y	.	.
Q13907	IDI1	up	up	.	.	.	.	.	Y	.	Y	.	.
P14902	IDO1	.	.	.	down	.	.	.	.	.	.	.	.

Q9Y6M1	IGF2BP2	.	.	down	down	.	.	.	.	.	Y	.	.
O00425	IGF2BP3	.	.	down	down	.	.	.	.	.	Y	.	.
D6RJC3	INPP4B	.	.	up	up	.	.	down	.	.	.	Y	.
P35568	IRS1	.	.	.	up	.	.	.	.	.	.	.	.
Q96J02	ITCH	.	down	.	.	.	.	.	.	.	Y	.	.
Q9P0J7	KCMF1	.	down	.	.	.	down	.	Y	.	.	.	.
P21583	KITLG	.	.	up	up	.	.	.	.	Y	.	.	.
P05783	KRT18	.	.	up	.	.	.	.	Y	Y	Y	.	.
O43790	KRT86	.	.	down	down	.	.	.	.	Y	.	Y	.
Q68DH5	LMBRD2	.	down	.	up	.	.	.	Y	.	.	.	.
P20700	LMNB1	.	.	down	.	.	.	.	Y	Y	Y	.	.
Q9H2I8	LRMDA|C10orf11	down	down	.	.	.	down	.	.	.	.	.	.
P10636	MAPT	.	.	.	up	up	.	down	Y	Y	.	.	.
B2CPU0	MATN3	.	.	.	up	.	.	down	.	.	.	.	.
Q9NU22	MDN1	.	.	.	down	.	.	.	.	.	Y	.	.
Q6P1Q9	METTL2B	.	up	.	.	.	.	.	.	.	.	.	.
Q9BRT3	MIEN1	up	up	.	.	.	up	.	Y	Y	Y	.	.
Q8N5J2	MINDY1|FAM63A	.	.	up	.	.	.	.	.	Y	.	.	.
J3KN01	MLLT4	.	down	.	.	.	.	.	.	.	.	.	.
H7C371	MLPH	.	.	up	up	.	.	.	.	.	.	.	.
Q9BV36	MLPH	.	.	up	.	.	.	.	Y	.	.	.	.
P09237	MMP7	.	.	down	.	.	.	.	Y	.	Y	.	.
P22894	MMP8	.	.	down	down	.	.	.	.	Y	.	.	.
P14780	MMP9	.	.	down	down	.	.	.	Y	Y	Y	Y	.
P05164	MPO	.	.	down	.	.	.	.	Y	Y	Y	Y	.
Q8WXI7	MUC16	.	.	down	.	.	.	.	Y	.	Y	.	.
J3KNX9	MYO18A	.	up	.	.	.	.	.	.	.	.	Y	.
E9PDF6	MYO1B	.	up	.	.	.	.	.	.	.	Y	.	.
B4DZ85	NCOA4	.	down	.	.	.	.	.	.	.	.	.	.
C9JE98	NCOR2	.	down	.	.	.	.	.	.	.	.	Y	.
O14777	NDC80	.	up	.	.	.	.	.	Y	.	.	.	.
E9PDL6	NDRG1	.	.	down	down	.	.	.	.	.	.	Y	.
Q7Z3B1	NEGR1	.	.	.	.	.	.	down	.	Y	.	.	.
E5RFJ1	NSMCE2	.	up	.	.	.	.	.	.	.	.	.	.
P18440	NT1	.	.	up	up	.	.	.	.	.	Y	.	.
Q9BZD4	NUF2	.	up	.	.	.	.	.	Y	.	.	.	.
Q9Y2J8	PADI2	.	up	down	down	.	.	up	Y	.	Y	.	.
A6NDB9	PALM3	.	.	.	up	.	.	.	.	.	.	.	.
Q9BVG4	PBDC1	.	up	.	.	.	.	.	Y	Y	.	Y	Y

P30039	PBLD	.	.	up	.	.	.	.	.	Y	Y	.	.
G5E9M0	PHYHD1	.	down	.	.	.	.	.	.	.	.	.	.
P78356	PIP4K2B	up	.	.	.	.	.	.	.	Y	Y	Y	.
Q9C010	PKIB	.	.	up	up	.	.	.	.	.	.	.	.
Q13393	PLD1	.	.	down	.	.	.	.	.	.	Y	.	.
E9PMV1	PLEC	.	down	.	.	.	.	.	.	.	Y	Y	.
B1ALD9	POSTN	.	.	.	.	.	.	down	Y	.	Y	.	.
F5H628	POSTN	.	.	.	.	.	.	down	Y	.	Y	.	.
Q9BZL4	PPP1R12C	.	down	.	.	.	.	.	.	.	.	.	.
P51888	PRELP	.	down	.	.	.	.	.	Y	Y	Y	.	Y
P05771	PRKCB	.	.	down	.	.	.	.	.	Y	Y	Y	.
O43490	PROM1	.	.	down	.	.	.	.	Y	.	Y	.	.
Q9Y617	PSAT1	.	.	down	down	.	.	.	Y	Y	Y	.	.
Q9P2B2	PTGFR.	.	down	.	.	.	.	.	Y	Y	Y	.	.
P26022	PTX3	.	.	down	.	.	.	.	Y	Y	Y	.	Y
Q2TAL8	QRICH1	.	down	.	.	.	.	.	.	.	.	.	.
Q01974	ROR2	.	down	.	.	.	.	.	.	.	.	.	.
Q96C34	RUNDC1	.	.	up	.	.	.	.	.	.	.	.	.
P05109	S100A8	.	.	down	down	.	.	.	Y	Y	Y	Y	Y
P06702	S100A9	.	.	down	.	.	.	.	Y	Y	Y	Y	Y
O76054	SEC14L2	.	.	.	.	.	.	down	Y	Y	.	.	.
J3KNL6	SEC16A	.	.	up	up	.	.	.	.	.	Y	.	.
Q15437	SEC23B	up	.	.	.	.	.	.	Y	.	Y	.	.
O15047	SETD1A	.	down	.	.	.	.	.	.	.	.	.	.
Q8N474	SFRP1	.	.	down	down	.	.	.	Y	Y	Y	.	Y
Q8TF72	SHROOM3	.	.	up	up	.	.	.	.	Y	Y	Y	.
Q6IA17	SIGIRR	.	.	up	.	.	.	.	Y	.	Y	.	.
Q9NTJ3	SMC4	.	.	.	.	.	.	up	Y	.	Y	.	.
Q9H4F8	SMOC1	.	.	down	.	.	.	.	Y	Y	.	.	.
H0Y6K5	SP3	.	down	.	.	.	down	.	.	.	.	.	.
E7EVV3	SPATA18	.	.	.	.	.	.	down	.	.	.	.	.
Q5T280	SPOUT1|C9orf11	.	down	.	.	.	.	.	.	.	.	.	.
F5H376	SRCIN1	up	up	.	.	.	.	.	.	.	.	.	.
F5H1Z6	STARD10	.	.	up	.	.	.	.	.	.	.	.	Y
Q13586	STIM1	.	down	.	.	.	.	.	Y	Y	Y	Y	.
Q9UNE7	STUB1	.	.	up	.	.	.	.	Y	.	Y	.	.
O00204	SULT2B1	.	.	up	up	.	.	.	Y	.	.	.	.
K7EJ35	SYNGR2	up	.	.	.	.	.	.	.	.	Y	Y	.
Q9UMS6	SYNPO2	.	.	.	.	.	.	down	.	.	.	.	.

J3KP28	SYTL2	.	.	up	.	.	.	.	.	.	.	.	.
Q9HCH5	SYTL2	.	.	up	up	.	.	.	.	.	Y	.	.
Q9Y2I9	TBC1D30	.	.	up	up	.	.	.	.	.	.	.	.
Q99426	TBCB	.	down	.	.	.	.	.	Y	Y	Y	Y	.
Q969E4	TCEAL3	.	down	up	up	.	.	.	Y	.	.	.	.
Q9Y6I9	TEX264	.	down	.	.	.	.	.	Y	Y	.	Y	.
P05549	TFAP2A	.	.	down	down	.	.	.	.	.	.	.	.
O43294	TGFB1I1	.	down	.	.	.	.	.	.	Y	.	Y	.
Q13769	THOC5	.	up	.	.	.	.	.	.	.	Y	.	.
Q5JTD0	TJAP1	.	down	.	.	.	.	.	.	.	.	.	.
Q9NW97	TMEM51	down	down	.	.	.	.	.	.	.	Y	.	.
Q9UQP3	TNN	.	.	.	.	.	.	down	.	Y	.	.	.
Q9NXH8	TOR4A	down	.	.	.	.	.	.	.	Y	Y	Y	.
Q5T7W7	TSTD2	.	up	.	.	.	.	.	.	.	.	.	.
E7EQL8	TUBGCP6	.	down	.	.	.	.	.	.	.	.	.	.
Q9BZM5	ULBP2	.	.	down	.	.	.	.	Y	.	.	.	.
Q15836	VAMP3	.	down	.	.	.	.	.	Y	Y	Y	Y	Y
Q7Z7G8	VPS13B	up	up	.	.	.	.	.	.	.	.	.	.
Q9H0M0	WWP1	.	down	.	.	.	.	.	.	.	Y	.	.
H0YIQ2	YLPM1	.	down	.	.	.	down	.	.	.	.	Y	.
Q6NZY4	ZCCHC8	.	down	.	.	.	.	.	Y	Y	.	.	.
Q96JP5	ZFP91	.	down	.	.	.	down	.	.	.	.	.	.
P17028	ZNF24	.	down	.	.	.	down	.	.	.	.	.	.
Q96PM9	ZNF385A	.	.	up	up	.	.	.	.	.	.	.	.
Q9H7S9	ZNF703	.	.	up	up	.	.	.	.	.	.	.	.
Q8TBC5	ZSCAN18	.	down	up	up	.	.	.	.	.	.	.	.
