# Supplementary material for: High‐throughput proteomics of breast cancer interstitial fluid: identification of tumor subtype‐specific serologically relevant biomarkers
Source: Mol Oncol. 2021 Jan 4;15(2):429–61. doi: 10.1002/1878-0261.12850 (PMC7858121; doi:10.1002/1878-0261.12850)
Supplement: Supplementary file 9 — Table S7. The results of the differential abundance analysis with limma. [file MOL2-15-429-s009.pdf]

Supplementary Table S7. This table contains the results of the differential abundance analysis with limma. There are eight sub-tables from contanst with BC subtypes (pairwise), esterorgen receptor, progesterone receptor, Her2 receptor, degree of tumor-infiltrating lymphocytes. Each table contains protein ID, gene name, tests-statistics, p-values and log fold changes (logFC). All proteins in this table were significantly DA after correction for multiple testing and filtering on logFC.

Her2 vs Lum

Accession	logFC	t	P.Value	adj.P.Val	B	dir	name
Q14451	3.354572728	7.990165844	3.11E-09	1.68E-05	10.62671491	up	GRB7
Q9BRT3	2.723692742	7.820738964	4.98E-09	1.68E-05	10.21399669	up	MIEN1
P04626	2.921066669	6.608826649	1.59E-07	0.000357458	7.146148059	up	ERBB2
Q9Y2J8	3.11288295	5.671380503	2.50E-06	0.003377471	4.666015699	up	PADI2
Q13907	1.672412594	5.560326671	3.47E-06	0.003910303	4.368692028	up	IDI1
Q14894	2.855619369	5.176794648	1.08E-05	0.0073047	3.340103598	up	CRYM
Q9NYV4	1.416137401	5.013586254	1.75E-05	0.009103053	2.902619238	up	CDK12
Q7Z7G8	1.105451861	4.949078224	2.12E-05	0.010225663	2.729915617	up	VPS13B
F5H376	1.923021564	4.69921659	4.42E-05	0.015165449	2.062918083	up	SRCIN1
Q01581	1.960543329	4.664613133	4.89E-05	0.015235723	1.970867254	up	HMGCS1
Q9ULI0	1.262276491	4.557622742	6.68E-05	0.016143142	1.686893407	up	ATAD2B
P37268	1.698351787	4.495878825	8.00E-05	0.017122041	1.523504895	up	FDFT1
Q5VZ73	1.848132415	4.475555017	8.49E-05	0.017401742	1.469810381	up	CCL21
P08243	1.654257345	4.365867444	0.000116791	0.0219404	1.180837063	up	ASNS
Q6P1Q9	1.704990507	4.282530133	0.000148647	0.025689116	0.96229798	up	METTL2B
Q9BVG4	1.0815031	4.175873151	0.000202119	0.031066589	0.684055381	up	PBDC1
Q5T7W7	1.032686605	4.08958627	0.000258832	0.036335792	0.46027813	up	TSTD2
E9PDF6	1.139325648	3.979602637	0.00035411	0.039745058	0.176960928	up	MYO1B
B0QY35	1.319200509	3.938661634	0.000397709	0.040134609	0.072091152	up	CSNK1E
E5RFJ1	1.232897582	3.937315152	0.000399228	0.040134609	0.068647909	up	NSMCE2
Q6ZNL6	1.677857454	3.865882477	0.000488495	0.042334686	-0.113476639	up	FGD5
Q9H2C0	1.152933602	3.847115471	0.000515005	0.042334686	-0.161142676	up	GAN
P38432	1.593065196	3.842555866	0.000521654	0.042334686	-0.172711764	up	COIL
Q13769	1.229038425	3.825523989	0.000547239	0.042962593	-0.215885511	up	THOC5
J3KNX9	1.548713716	3.804386409	0.000580694	0.044126256	-0.26937508	up	MYO18A
O14777	1.250180932	3.718010165	0.000739292	0.049750774	-0.486859995	up	NDC80
Q9BZD4	1.388004306	3.717191851	0.000740979	0.049750774	-0.488911741	up	NUF2
O75363	2.353540003	3.714309047	0.000746953	0.049750774	-0.496138423	up	BCAS1
Q9NW97	-2.068450559	-5.936489223	1.14E-06	0.001927988	5.373658679	down	TMEM51
H0Y6K5	-1.926238151	-5.504795098	4.09E-06	0.003950868	4.219880866	down	SP3
Q68DH5	-1.842027413	-5.231543549	9.19E-06	0.0073047	3.486963134	down	LMBRD2
Q9H2I8	-2.155769589	-5.183872685	1.06E-05	0.0073047	3.359088066	down	LRMDA|C10orf11
Q9BZL4	-1.328704264	-5.094620552	1.38E-05	0.007922154	3.119757747	down	PPP1R12C
Q63HQ0	-1.164320551	-5.087706213	1.41E-05	0.007922154	3.10122339	down	AP1AR
H0YIQ2	-1.483576965	-4.896620317	2.47E-05	0.01114049	2.589597853	down	YLPM1
Q96JP5	-1.595430444	-4.843195895	2.89E-05	0.011824613	2.446830935	down	ZFP91
E7EQL8	-1.505068917	-4.833890771	2.97E-05	0.011824613	2.421980259	down	TUBGCP6
B4DDP6	-1.350915035	-4.771408708	3.57E-05	0.013420805	2.255245217	down	NA
Q2TAL8	-1.816702052	-4.654459597	5.03E-05	0.015235723	1.943875243	down	QRICH1
Q9P0J7	-1.393193464	-4.633611552	5.35E-05	0.015235723	1.888479817	down	KCMF1
O15047	-1.196954365	-4.618793024	5.59E-05	0.015235723	1.84912789	down	SETD1A
E9PG22	-1.529552484	-4.60328198	5.85E-05	0.015235723	1.807957502	down	CEP97

Q5JVS0	-1.281186822	-4.597104772	5.96E-05	0.015235723	1.791567552	down	HABP4
Q969E4	-1.579172138	-4.589861092	6.08E-05	0.015235723	1.772352349	down	TCEAL3
Q01974	-1.321479087	-4.529270813	7.26E-05	0.016555129	1.611819712	down	ROR2
O43294	-1.280677333	-4.491679153	8.10E-05	0.017122041	1.512405917	down	TGFB1I1
Q6NZY4	-1.138524892	-4.43235417	9.63E-05	0.019152522	1.355827946	down	ZCCHC8
Q5JTD0	-1.276279419	-4.379099192	0.000112393	0.021717603	1.215619217	down	TJAP1
Q15836	-1.16690882	-4.277088532	0.000151002	0.025689116	0.948061363	down	VAMP3
Q13057	-1.475862884	-4.274945817	0.000151939	0.025689116	0.94245663	down	COASY
B7Z4R0	-1.061709797	-4.257975974	0.000159567	0.026132327	0.898091666	down	ERF
H0YDQ8	-1.086613381	-4.252112759	0.000162289	0.026132327	0.882772937	down	CRTC2
J3QT29	-1.980694463	-4.231159686	0.000172394	0.027113934	0.828070678	down	COPS9
Q5T280	-1.144387734	-4.11468512	0.000240895	0.035416846	0.525239838	down	SPOUT1|C9orf114
Q13586	-1.361153271	-4.100822643	0.000250646	0.03606633	0.489346968	down	STIM1
Q8WUD4	-1.302608912	-4.082880245	0.000263842	0.036335792	0.442940009	down	CCDC12
Q9Y6I9	-1.4274222	-4.041146914	0.000297223	0.037284905	0.335221684	down	TEX264
Q9UI08	-1.753329439	-4.039446135	0.000298667	0.037284905	0.330838524	down	EVL
Q99426	-1.015263502	-4.027815594	0.000308732	0.037284905	0.300879316	down	TBCB
Q9UKB3	-1.668626197	-3.984949089	0.000348773	0.039745058	0.190680399	down	DNAJC12
P50895	-1.293595793	-3.984857752	0.000348863	0.039745058	0.190445972	down	BCAM
Q9H0M0	-1.309660009	-3.984185216	0.000349531	0.039745058	0.188719891	down	WWP1
Q96RK0	-1.201653471	-3.963807066	0.000370348	0.039971172	0.136461036	down	CIC
Q14004	-1.037469991	-3.947131894	0.000388279	0.040134609	0.093759913	down	CDK13
P17028	-1.440793249	-3.931480628	0.000405878	0.040134609	0.053732073	down	ZNF24
Q14C86	-1.058849519	-3.900463768	0.000443086	0.041619322	-0.025443255	down	GAPVD1
G3V3R7	-1.177220319	-3.88522322	0.000462567	0.042334686	-0.06427275	down	ATXN3
Q9NSA3	-1.217529786	-3.862845325	0.000492693	0.042334686	-0.121195915	down	CTNNBIP1
G8JLD5	-1.49181449	-3.850381049	0.000510293	0.042334686	-0.152854081	down	DNM1L
E9PMV1	-1.41367571	-3.846588695	0.000515769	0.042334686	-0.162479503	down	PLEC
P51888	-2.016763341	-3.839728711	0.000525819	0.042334686	-0.179882775	down	PRELP
Q96J02	-1.016403605	-3.826256854	0.000546114	0.042962593	-0.214029136	down	ITCH
C9JE98	-1.290511646	-3.817951632	0.000559003	0.042962593	-0.235059442	down	NCOR2
Q9P2B2	-1.308529865	-3.817935457	0.000559028	0.042962593	-0.235100384	down	PTGFRN
J3KN01	-1.744008397	-3.767593628	0.000643732	0.047321285	-0.362233338	down	MLLT4
O94769	-1.593993248	-3.727986623	0.000719019	0.049750774	-0.461832805	down	ECM2
G5E9M0	-1.052435265	-3.724694687	0.000725648	0.049750774	-0.470093762	down	PHYHD1
Q8TBC5	-1.245842839	-3.713439629	0.000748763	0.049750774	-0.498317492	down	ZSCAN18
B4DZ85	-1.231103062	-3.713092312	0.000749488	0.049750774	-0.49918794	down	NCOA4

Her2 vs TNBC

Accession	logFC	t	P.Value	adj.P.Val	B	dir	name
Q9BRT3	2.9398202	7.7710164	5.72E-09	3.87E-05	9.77674	up	MIEN1
P04626	3.1573192	6.5761054	1.74E-07	0.0005898	6.8636384	up	ERBB2
Q14451	2.7774513	6.0902122	7.25E-07	0.0016337	5.6290195	up	GRB7
F5H376	2.5564068	5.7509375	1.97E-06	0.0033363	4.7549023	up	SRCIN1
Q13907	1.6635398	5.0916372	1.39E-05	0.0147063	3.0410434	up	IDI1
O75363	3.4833127	5.0607665	1.52E-05	0.0147063	2.9606624	up	BCAS1
Q15437	1.0832039	4.757115	3.73E-05	0.0314922	2.171344	up	SEC23B
F8W8P5	1.9984952	4.6852767	4.60E-05	0.0329099	1.9851989	up	CADPS2
K7EJ35	2.1689829	4.666073	4.87E-05	0.0329099	1.9354934	up	SYNGR2
P78356	1.0685668	4.5862764	6.15E-05	0.0348891	1.7292312	up	PIP4K2B
Q14894	2.7466389	4.5838412	6.19E-05	0.0348891	1.7229441	up	CRYM
Q9NYV4	1.3747661	4.4806322	8.37E-05	0.0435252	1.4569471	up	CDK12
Q01581	2.0044854	4.3904543	0.0001088	0.0480373	1.225363	up	HMGCS1
P14735	1.0996354	4.3741442	0.000114	0.0480373	1.1835696	up	IDE
P83436	1.2082988	4.3734472	0.0001143	0.0480373	1.1817843	up	COG7
Q7Z7G8	1.0492897	4.3246133	0.0001316	0.0480373	1.0568372	up	VPS13B
P37268	1.7501983	4.2652215	0.0001563	0.0480373	0.9052671	up	FDFT1
Q9NW97	-1.996696	-5.275502	8.06E-06	0.0109082	3.5198858	down	TMEM51
Q9NXH8	-1.44615	-4.351781	0.0001217	0.0480373	1.1263132	down	TOR4A
Q9H4G4	-1.524703	-4.297522	0.0001423	0.0480373	0.9876442	down	GLIPR2
Q9H2I8	-1.939798	-4.294137	0.0001437	0.0480373	0.9790061	down	LRMDA|C10orf11

Luminal vs TNBC

Accession	logFC	t	P.Value	adj.P.Val	B	dir	name
Q969E4	1.373693313	6.323726658	3.65E-07	0.001233638	6.453978112	up	TCEAL3
Q8IV36	1.585085321	6.132731423	6.39E-07	0.001318026	5.94309259	up	HID1
Q96C34	1.337618783	5.694956343	2.33E-06	0.002353623	4.763186042	up	RUNDC1
Q8TBC5	1.202694288	5.677819629	2.45E-06	0.002353623	4.716811954	up	ZSCAN18
P53365	1.191531895	5.634615916	2.78E-06	0.002353623	4.599853584	up	ARFIP2
Q96MH2	1.011732232	5.360530328	6.27E-06	0.004100818	3.85687087	up	HEXIM2
P50895	1.089662568	5.316422611	7.14E-06	0.004100818	3.737229189	up	BCAM
Q9H7S9	1.449387537	5.214632657	9.66E-06	0.004278878	3.461155372	up	ZNF703
P05783	1.33680455	5.210757743	9.77E-06	0.004278878	3.450647843	up	KRT18
J3KNL6	1.136238425	5.210151778	9.79E-06	0.004278878	3.449004679	up	SEC16A
Q9UKB3	1.35905828	5.140614494	1.20E-05	0.004278878	3.26048437	up	DNAJC12
P23771	1.560992242	5.03717349	1.63E-05	0.005256005	2.980258721	up	GATA3
Q6IA17	1.155750053	4.888964423	2.53E-05	0.006154697	2.579439017	up	SIGIRR
Q8WU20	1.265346914	4.82440632	3.06E-05	0.006890289	2.405192282	up	FRS2
Q9UNE7	1.066116252	4.760571803	3.69E-05	0.007793496	2.233155679	up	STUB1
J3QT29	1.402208002	4.744250428	3.87E-05	0.007928525	2.189214351	up	COPS9
O00204	1.468123149	4.687769937	4.57E-05	0.008256716	2.037310517	up	SULT2B1
F5H1Z6	1.155362417	4.546588724	6.90E-05	0.010609342	1.658810326	up	STARD10
Q8N5J2	1.062377004	4.519223324	7.48E-05	0.010852589	1.585670247	up	MINDY1|FAM63A
Q8TD06	1.773649242	4.439384574	9.43E-05	0.011891429	1.37275657	up	AGR3
D6RJC3	1.320038204	4.424657763	9.85E-05	0.011891429	1.333564641	up	NA
Q96DG6	1.072488957	4.415335084	0.000101169	0.011972535	1.308768277	up	CMBL
Q9H993	1.267284215	4.372098501	0.000114699	0.012387526	1.193911548	up	ARMT1

Q9C010	1.567443123	4.37001384	0.000115395	0.012387526	1.188379812	up	PKIB
Q96PM9	1.093084443	4.341951983	0.000125172	0.012826361	1.113972914	up	ZNF385A
P21266	1.133260854	4.297006314	0.000142557	0.01388187	0.995023746	up	GSTM3
H7C371	1.531607322	4.256251188	0.000160363	0.014655851	0.887416379	up	NA
Q14353	1.057272607	4.224929155	0.000175516	0.015025514	0.804885723	up	GAMT
O95994	1.647661556	4.200170362	0.000188482	0.01544107	0.739757157	up	AGR2
Q15847	1.252462919	4.18332647	0.000197836	0.015928139	0.695505211	up	ADIRF
Q9Y2I9	1.323944426	4.136526734	0.000226282	0.016210527	0.572800944	up	TBC1D30
P18440	1.535095037	4.022442763	0.000313493	0.018436104	0.275305591	up	NAT1
P30039	1.036694601	3.990599681	0.000343218	0.019355805	0.192706937	up	PBLD
P80404	1.143680338	3.97204175	0.000361794	0.019892771	0.144662035	up	ABAT
Q9BV36	1.099440134	3.957225981	0.000377326	0.020408687	0.106355481	up	MLPH
Q9NYQ6	1.103334271	3.935134601	0.000401701	0.021391374	0.04932189	up	CELSR1
Q8TF72	1.030697766	3.926906707	0.000411167	0.021724395	0.028105974	up	SHROOM3
Q9HCH5	1.126111469	3.831773823	0.000537712	0.024738413	-0.216126864	up	SYTL2
O43570	1.215215897	3.816687148	0.000560991	0.025624815	-0.254671026	up	CA12
P21583	1.179398295	3.549809922	0.001177194	0.038490241	-0.927046605	up	KITLG
J3KP28	1.161548446	3.432808494	0.001620034	0.04654106	-1.215545516	up	NA
Q8N474	-1.607596643	-6.065440196	7.80E-07	0.001318026	5.762454903	down	SFRP1
Q9Y6M1	-1.334080603	-5.855649694	1.45E-06	0.001958362	5.197468778	down	IGF2BP2
O00425	-1.979113675	-5.156533098	1.15E-05	0.004278878	3.303632808	down	IGF2BP3
P26022	-1.18856541	-4.936229132	2.20E-05	0.006154697	2.707154416	down	PTX3
P14780	-1.553283069	-4.904291453	2.42E-05	0.006154697	2.620842118	down	MMP9
Q16790	-1.930282789	-4.771073217	3.58E-05	0.007793496	2.261438316	down	CA9
E9PDL6	-1.100947512	-4.671580517	4.79E-05	0.008256716	1.993816397	down	NA

P22894	-1.716827793	-4.434672828	9.56E-05	0.011891429	1.360214501	down	MMP8
P05771	-1.041325018	-4.375980777	0.000113415	0.012387526	1.20421485	down	PRKCB
Q9Y2J8	-1.48240698	-4.277664131	0.000150751	0.013988179	0.943923235	down	PADI2
O43490	-1.311324662	-4.277118722	0.000150989	0.013988179	0.942483101	down	PROM1
P49913	-2.050809651	-4.243949251	0.000166154	0.014766097	0.854983921	down	CAMP
P05109	-1.898813809	-4.155679937	0.000214185	0.016210527	0.622974108	down	S100A8
P05164	-1.494084372	-4.151233545	0.000216936	0.016210527	0.611320897	down	MPO
O15540	-2.063244772	-4.133598233	0.00022819	0.016210527	0.565135078	down	FABP7
Q9NQW6	-1.197235866	-3.954521957	0.00038023	0.020408687	0.099369022	down	ANLN
Q9BZM5	-1.003106992	-3.913080456	0.000427566	0.021916701	-0.007513117	down	ULBP2
B1AM48	-1.038845933	-3.911584624	0.000429378	0.021916701	-0.011364216	down	ELAVL2
Q13393	-1.0348629	-3.864639169	0.00049021	0.023347094	-0.131979869	down	PLD1
Q9H4F8	-1.376374576	-3.846995739	0.000515178	0.02402863	-0.177183916	down	SMOC1
Q8WXI7	-1.69114569	-3.748614784	0.0006788	0.02942771	-0.427913145	down	MUC16
F5H2U1	-1.221041095	-3.694058069	0.000790253	0.032195679	-0.56593217	down	NA
O43790	-1.598424562	-3.639996709	0.000918108	0.034306136	-0.701936938	down	KRT86
P06702	-1.011149908	-3.620832539	0.000968071	0.035199395	-0.749960972	down	S100A9
P20700	-1.026641839	-3.590802367	0.001051695	0.036012902	-0.825010883	down	LMNB1
Q9Y617	-1.215392709	-3.586239371	0.001064998	0.036012902	-0.836392443	down	PSAT1
P05549	-1.151397658	-3.528195366	0.00124907	0.039846504	-0.98065278	down	TFAP2A
P24298	-1.123483914	-3.430329107	0.001630967	0.04654106	-1.221613562	down	GPT
P09237	-1.447072153	-3.420612476	0.001674501	0.046990259	-1.245375229	down	MMP7
Q9BXR6	-1.001325579	-3.406723087	0.001738675	0.047799437	-1.279288897	down	CFHR5
P54108	-1.149893329	-3.401584384	0.001763012	0.048077615	-1.291820338	down	CRISP3

ER+ vs ER-

Accession	logFC	t	P.Value	adj.P.Val	B	dir	name
Q969E4	1.424071326	7.294946495	1.84E-08	0.00012476	9.209136871	up	TCEAL3
Q8TBC5	1.21327318	6.400872069	2.56E-07	0.000864723	6.807785614	up	ZSCAN18
P50895	1.13966164	6.183581607	4.89E-07	0.000926403	6.213010716	up	BCAM
Q9UKB3	1.434956205	6.027638808	7.79E-07	0.000926403	5.784218807	up	DNAJC12
Q9H7S9	1.482061964	5.956198166	9.65E-07	0.000926403	5.587327491	up	ZNF703
J3QT29	1.544037694	5.732181247	1.89E-06	0.001277711	4.96849062	up	COPS9
P23771	1.534264548	5.534072991	3.42E-06	0.001699172	4.420006341	up	GATA3
J3KNL6	1.064181946	5.405085348	5.05E-06	0.001795814	4.062644158	up	SEC16A
Q8IV36	1.327778685	5.316154678	6.59E-06	0.002157209	3.816286093	up	HID1
Q68DH5	1.14475766	5.309526056	6.72E-06	0.002157209	3.797926723	up	LMBRD2
P53365	1.009002156	5.026237402	1.57E-05	0.004086038	3.014339612	up	ARFIP2
Q9H479	1.004615982	4.954964064	1.94E-05	0.004240666	2.817699952	up	FN3K
P21860	1.020555552	4.902241581	2.28E-05	0.00452557	2.672428369	up	ERBB3
Q9H993	1.265909455	4.884425376	2.40E-05	0.004636242	2.623377525	up	ARMT1
Q8TD06	1.729246978	4.837155667	2.76E-05	0.005189511	2.493342531	up	AGR3
Q8TF72	1.147285384	4.808337953	3.01E-05	0.005501739	2.414147733	up	SHROOM3
Q9NYQ6	1.217635838	4.789883945	3.18E-05	0.005536516	2.363467613	up	CELSR1
O00204	1.342526918	4.730781172	3.79E-05	0.006030062	2.201343636	up	SULT2B1
Q9Y2I9	1.34813568	4.709091959	4.04E-05	0.006030062	2.141924915	up	TBC1D30
O43570	1.360337654	4.697012202	4.19E-05	0.006030062	2.108850564	up	CA12
G8JLD5	1.03023498	4.566105767	6.18E-05	0.008031258	1.751366793	up	DNM1L
P18440	1.552859599	4.551182699	6.45E-05	0.008234859	1.710732114	up	NAT1
P80404	1.141225447	4.432715205	9.15E-05	0.009375188	1.389115601	up	ABAT

Q96PM9	1.001534845	4.399434111	0.000100887	0.009888437	1.299094439	up	ZNF385A
H7C371	1.380094291	4.227316309	0.000166852	0.013014916	0.836169144	up	MLPH
Q9C010	1.382502899	4.217878101	0.000171498	0.013031888	0.81092237	up	PKIB
Q9HCH5	1.094777458	4.162273751	0.000201555	0.014537323	0.662499835	up	SYTL2
Q9UI08	1.047685451	4.042780526	0.000284672	0.017943233	0.345489223	up	EVL
D6RJC3	1.100668622	3.949597251	0.000371954	0.020842093	0.100287522	up	INPP4B
P21583	1.164715206	3.920638822	0.000404047	0.020859291	0.02447235	up	KITLG
P10636	1.06497005	3.878432173	0.000455704	0.022353303	-0.085683944	up	MAPT
O95994	1.390324154	3.827992543	0.000525909	0.023870609	-0.216774324	up	AGR2
A6NDB9	1.194028335	3.691947354	0.000771799	0.028434393	-0.567150076	up	PALM3
P35568	1.038764115	3.691175139	0.000773472	0.028434393	-0.569124864	up	IRS1
B2CPU0	1.067083873	3.663587435	0.000835586	0.029742458	-0.639566846	up	MATN3
Q9Y2J8	-1.88215688	-5.488709407	3.92E-06	0.001699172	4.294332423	up	PADI2
Q9NU22	-1.031294172	-5.417264297	4.86E-06	0.001795814	4.096386802	down	MDN1
E9PDL6	-1.113313712	-5.280347403	7.34E-06	0.002157209	3.717118341	down	NA
Q9Y6M1	-1.008061457	-4.263320424	0.000150239	0.012273598	0.932616805	down	IGF2BP2
Q8N474	-1.179667382	-4.146308768	0.000211101	0.014718327	0.619987904	down	SFRP1
P05549	-1.147747013	-3.933916551	0.00038901	0.020842093	0.059210921	down	TFAP2A
Q16790	-1.518004217	-3.871115609	0.00046529	0.022636236	-0.104737254	down	CA9
O00425	-1.470248026	-3.762094409	0.000633636	0.026129743	-0.387092101	down	IGF2BP3
P14780	-1.181609248	-3.757958481	0.000641069	0.026276042	-0.39774464	down	MMP9
Q9Y617	-1.127677507	-3.701111926	0.000752214	0.028434393	-0.543701011	down	PSAT1
P22894	-1.361144219	-3.679231299	0.000799794	0.029023313	-0.599647981	down	MMP8
O43790	-1.448807055	-3.655529551	0.000854622	0.030103156	-0.660101636	down	KRT86
Q14451	-1.258079531	-3.648331492	0.000871981	0.03039796	-0.67842975	down	GRB7

P49913	-1.64534096	-3.600994745	0.000994934	0.031788753	-0.798591482	down	CAMP
P14902	-1.173330403	-3.534023172	0.001197865	0.036005146	-0.96746507	down	IDO1
P05109	-1.507759675	-3.482337515	0.001381206	0.03908409	-1.096851928	down	S100A8

PgR+ vs PgR-

Accession	logFC	t	P.Value	adj.P.Val	B	dir	name
P50895	1.16784994	6.145613187	5.48E-07	0.003709081	5.819657804	up	BCAM
J3QT29	1.455326682	4.897942114	2.31E-05	0.02600247	2.56808306	up	COPS9
P10636	1.23431186	4.662748657	4.64E-05	0.044858759	1.955515739	up	MAPT
G3XAP6	1.326345699	4.584053748	5.86E-05	0.049548042	1.751375408	up	COMP

High Her2 vs Low Her2

Accession	logFC	AveExpr	t	P.Value	adj.P.Val	B	dir	name
P04626	2.106844836	-0.472077241	5.470594165	4.18E-06	0.01373284	3.997661	up	ERBB2
Q14451	2.027962171	-0.726499877	4.745914375	3.64E-05	0.04487225	2.132305	up	GRB7
Q9BRT3	1.658632363	-0.308486661	4.548925495	6.53E-05	0.049043114	1.628201	up	MIEN1
H0Y6K5	-1.612491459	-0.072742711	-6.122248548	5.93E-07	0.004009871	5.662717	down	SP3
P17028	-1.388675309	-0.202148735	-5.344655615	6.09E-06	0.01373284	3.673514	down	ZNF24
Q96JP5	-1.250924518	-0.094188503	-4.9125969	2.22E-05	0.03750885	2.560581	down	ZFP91
Q9H2I8	-1.567943966	-0.043569347	-4.70810441	4.08E-05	0.04487225	2.035347	down	LRMDA|C10orf11
H0YIQ2	-1.133932071	-0.061252575	-4.634878509	5.06E-05	0.04487225	1.847824	down	YLPM1
Q9P0J7	-1.073632237	0.020659762	-4.618889074	5.31E-05	0.04487225	1.806925	down	KCMF1

High TILs vs Low TILs

Accession	logFC	t	P.Value	adj.P.Val	B	dir	name
Q9NTJ3	1.0099165	5.17404115	1.01E-05	0.023628855	3.348808804	up	SMC4
Q9Y2J8	1.508988	3.90476085	0.00042319	0.043206258	0.021826905	up	PADI2
P10636	-1.2549822	-5.1285114	1.16E-05	0.023628855	3.227402003	down	MAPT
P04792	-1.0112035	-4.9282789	2.11E-05	0.023628855	2.694112091	down	HSPB1
B1ALD9	-1.1711345	-4.890194	2.36E-05	0.023628855	2.592850393	down	POSTN
F5H628	-1.3914263	-4.888721	2.37E-05	0.023628855	2.588935494	down	POSTN
E7EVV3	-1.0899967	-4.7805968	3.27E-05	0.024607865	2.301880347	down	SPATA18
P19883	-1.2577421	-4.5396047	6.69E-05	0.027821288	1.665091464	down	FST
E7EUD0	-1.0172224	-4.4006055	0.00010068	0.031930105	1.300337727	down	DKK3
P25940	-1.0666356	-4.3463515	0.00011803	0.031930105	1.158589878	down	COL5A3
Q9UMS6	-1.0609608	-4.2474414	0.00015755	0.032795625	0.901192013	down	SYNPO2
O76054	-1.0952554	-4.2453819	0.0001585	0.032795625	0.895847365	down	SEC14L2
E7EQD6	-1.774282	-4.1546746	0.00020629	0.037992375	0.661105884	down	CEGP1
D6RJC3	-1.1218425	-4.1359196	0.0002178	0.037992375	0.612735364	down	INPP4B
Q7Z3B1	-1.1692093	-4.1296277	0.00022181	0.037992375	0.596521336	down	NEGR1
Q9UQP3	-1.1876356	-4.1071102	0.00023673	0.037992375	0.538550296	down	TNN
Q16610	-1.0644286	-4.0921547	0.00024718	0.037992375	0.500096316	down	ECM1
B2CPU0	-1.0855654	-3.8179736	0.00054156	0.045668472	-0.19721747	down	MATN3

High Grade vs Low Grade

Accession	logFC	AveExpr	t	P.Value	adj.P.Val	B	dir	name
E7EQL8	-0.86941022	0.08025458	-4.254905	0.000154194	0.107952858	0.8554155	down	TUBGCP6
E9PFN4	-0.88005708	0.00969361	-4.045146	0.000283095	0.107952858	0.3374224	down	SLC4A7
E9PMV1	-1.04286148	-0.0209261	-4.370934	0.000109855	0.107952858	1.1444277	down	PLEC
H0Y6K5	-1.05030253	-0.0727427	-4.155118	0.000206059	0.107952858	0.608206	down	SP3
O43570	-1.51062141	-0.3023773	-4.766102	3.42E-05	0.107952858	2.1379815	down	CA12
O94910	-1.10362298	-0.2869269	-4.734554	3.76E-05	0.107952858	2.058277	down	ADGRL1
Q13586	-0.8408651	-0.0079982	-3.9885	0.000333128	0.107952858	0.1986968	down	STIM1
Q5T013	-0.93040701	0.06322512	-4.028516	0.000296968	0.107952858	0.2966379	down	HYI
Q63HQ0	-0.69819705	0.01756123	-4.460267	8.45E-05	0.107952858	1.3679287	down	AP1AR
Q8NE01	-0.8610068	-0.0556524	-4.15597	0.000205552	0.107952858	0.61031	down	CNNM3
Q8TB36	-1.24555853	0.12296138	-4.108467	0.000235841	0.107952858	0.4931129	down	GDAP1
Q92870	-0.86702075	0.114145	-4.012158	0.000311262	0.107952858	0.2565676	down	APBB2
Q96MH2	-0.88990368	0.03572679	-4.110107	0.000234727	0.107952858	0.4971518	down	HEXIM2
Q9NYQ6	-1.24596794	-0.0840087	-4.262066	0.000151009	0.107952858	0.8732068	down	CELSR1
C9J2C3	1.04269711	-0.1311889	4.2410856	0.000160526	0.107952858	0.8211016	up	GALNT3
E5RFJ1	0.86580015	-0.0280972	4.0711549	0.000262659	0.107952858	0.4012943	up	NSMCE2
P08243	1.07003822	-0.1083467	4.5444279	6.59E-05	0.107952858	1.5791647	up	ASNS
Q9UGI8	0.61136479	0.06941302	3.9863303	0.000335208	0.107952858	0.1933932	up	TES
Q9Y2J8	1.81745368	-0.4535788	4.3901601	0.000103835	0.107952858	1.1924609	up	PADI2
