# Supplementary material for: High‐throughput proteomics of breast cancer interstitial fluid: identification of tumor subtype‐specific serologically relevant biomarkers
Source: Mol Oncol. 2021 Jan 4;15(2):429–61. doi: 10.1002/1878-0261.12850 (PMC7858121; doi:10.1002/1878-0261.12850)
Supplement: Supplementary file 10 — Table S8. Results from differential abundance analysis, LASSO regression, and random forest. [file MOL2-15-429-s010.pdf]

Supplementary Table S8: *Results from differential abundance analysis, LASSO regression, and random forest.* Only proteins identified by at least two out of the three methods are included. Fourteen proteins are listed in bold font, and those with an asterisk were selected for further analysis. Arrows denote abundance directionality (up or down) in TIF samples for comparison. Arrows in parentheses are borderline significant (i.e., corrected p-value < 0.05, yet log-fold change was just shy of either 1 or -1). # indicate 2 proteins, GPC1 and HSPB1, which were excluded from the list (see Chapter 3.4)

Accession (SwissProt)	Gene Symbol	Differential Abundance Analysis	LASSO Regression	Random Forest	BC Subtypes			ER+	PgR+	Her2	High TILs (+3/+2)
					Luminal	Her2	TNBC				
Q8TD06	**AGR3***	Yes	Yes	No	↑	.	↓	.	.	.	.
P50895	**BCAM***	Yes	Yes	Yes	↑	↓	↓	.	↑	.	.
Q9NYQ6	**CELSR1***	Yes	No	Yes	↑	(↓)	↓	↑	.	.	.
Q7Z3D4	**LYSMD3**	No	Yes	Yes	↑	.	↓	.	.	.	.
Q9BRT3	**MIEN1***	Yes	Yes	No	↓	↑	↓	.	.	.	.
P18440	**NAT1***	Yes	Yes	No	↑	.	↓	.	.	.	.
Q9BVG4	**PBDC1**	Yes	Yes	No	↓	↑	↓	.	.	.	.
P78356	**PIP4K2B***	Yes	Yes	No	.	↑	↓	.	.	.	.
Q15437	**SEC23B***	Yes	Yes	No	.	↑	↓	.	.	.	.
Q969E4	**TCEAL3**	Yes	No	Yes	↑	↓	↑	.	.	.	.
Q9BU02	**THTPA***	No	Yes	Yes	↑	.	↓	↑	.	.	.
Q9NW97	**TMEM51***	Yes	Yes	No	↑	↓	↑	.	.	.	.
Q9BZM5	**ULBP2***	Yes	Yes	No	↓	.	↑	.	.	.	.
H0YIQ2	**YLPM1**	Yes	Yes	No	↑	↓	.	.	.	.	.
G3XAP6	COMP	Yes	Yes	No	.	.	.	.	↑	.	.
Q969E4	TCEAL3	Yes	Yes	No	.	.	.	↑	.	.	.
Q8TBC5	ZSCAN18	Yes	Yes	No	.	.	.	↑	.	.	.
Q9H7S9	ZNF703	Yes	Yes	No	.	.	.	↑	.	.	.
P04626	ERBB2	Yes	Yes	No	.	.	.	.	.	↑	.
H0Y6K5	SP3	Yes	Yes	No	.	.	.	.	.	↓	.
P17028	ZNF24	Yes	No	Yes	.	.	.	.	.	↓	.
P25940	COL5A3	Yes	Yes	No	.	.	.	.	.	.	↓
P35052	GPC1#	No	Yes	Yes	.	.	.	.	.	.	↓
P04792	HSPB1#	Yes	Yes	No	.	.	.	.	.	.	↓
P10636	MAPT	Yes	Yes	No	.	.	.	.	.	.	↓
E7EVV3	SPATA18	Yes	Yes	No	.	.	.	.	.	.	↓
