# Supplementary material for: High‐throughput proteomics of breast cancer interstitial fluid: identification of tumor subtype‐specific serologically relevant biomarkers
Source: Mol Oncol. 2021 Jan 4;15(2):429–61. doi: 10.1002/1878-0261.12850 (PMC7858121; doi:10.1002/1878-0261.12850)
Supplement: Supplementary file 11 — Table S9. Comparison of the expression profiles for eight of the TIF proteins identified in the present study with the PAM50 prognostic signature. [file MOL2-15-429-s011.pdf]

Supplementary Table S9. *Comparison of the expression profiles for eight of the TIF proteins identified in the present study with the PAM50 prognostic signature*. Entries in the table are sorted by protein set, and within the sets proteins are sorted alphabetically.

Gene Symbol	Directionality in TIF	Directionality in PAM50 signature
ANLN	Up in TNBC *vs* Luminal	Up in Basal-like and Her2
SFRP1	Up in TNBC *vs* Luminal	Up in Basal-like
ERBB2	Up in Her2 *vs* TNBC	Up in Her2
GRB7	Up in Her2 *vs* TNBC	Up in Her2
MLPH	Up in Luminal *vs* TNBC	Up in Luminal
NAT1	Up in Luminal *vs* TNBC	Up in Luminal
NDC80	Up in Her2 *vs* Luminal	Up in Her2 and Basal-like
NUF2	Up in Her2 *vs* Luminal	Up in Her2 and Basal-like
