# Supplementary material for: High‐throughput proteomics of breast cancer interstitial fluid: identification of tumor subtype‐specific serologically relevant biomarkers
Source: Mol Oncol. 2021 Jan 4;15(2):429–61. doi: 10.1002/1878-0261.12850 (PMC7858121; doi:10.1002/1878-0261.12850)
Supplement: Supplementary file 12 — Table S10. Protein‐protein interaction networks from analysis using the STRING database. [file MOL2-15-429-s012.pdf]

Supplementary Table S10. Protein-protein interaction networks from analysis using the STRING database. Networks included in the table are from sets of proteins differentially abundant between BC subtypes (Luminal, Her2 and TNBC). Each table contains protein node pairs, logFCs, adjusted p-values, STRING score (support for interaction) and rank (based on sum of absolute logFC of both node pair).

Luminal vs TNBC

node1	node2	score	logFC.node1	fdr.node1	dir.node1	logFC.node2	fdr.node2	dir.node2	comparison	rank
KRT86	KRT18	908	-1.59842456182153	0.034306135658	down	1.33680454995867	0.00427887797	up	Lum vs TNBC	99.75
NAT1	MPO	922	1.53509503707609	0.018436103610	up	-1.4940843722041	0.01621052705	down	Lum vs TNBC	99.5
RUNDC1	HID1	389	1.33761878271438	0.002353623305	up	1.58508532129184	0.00131802560	up	Lum vs TNBC	98.25
MMP9	MMP7	950	-1.55328306914342	0.00615469728	down	-1.4470721525541	0.04699025864	down	Lum vs TNBC	97.5
MMP7	GATA3	160	-1.4470721525541	0.046990258645	down	1.56099224227678	0.00525600525	up	Lum vs TNBC	97.25
SFRP1	PROM1	300	-1.60759664272772	0.00131802560	down	-1.3113246616220	0.01398817948	down	Lum vs TNBC	96.5
CA12	AGR3	336	1.21521589710955	0.025624814650	up	1.77364924191116	0.01189142896	up	Lum vs TNBC	96.25
AGR2	ADIRF	311	1.64766155608393	0.015441070152	up	1.25246291884499	0.01592813945	up	Lum vs TNBC	95.75
SULT2B1	NAT1	180	1.46812314870425	0.008256715953	up	1.53509503707609	0.0184361036	up	Lum vs TNBC	93.75
PADI2	MPO	923	-1.48240698028328	0.013988179488	down	-1.4940843722041	0.01621052705	down	Lum vs TNBC	93.5
KRT18	GATA3	474	1.33680454995867	0.004278877978	up	1.56099224227678	0.00525600525	up	Lum vs TNBC	92.5
MMP9	KRT18	371	-1.55328306914342	0.00615469728	down	1.33680454995867	0.00427887797	up	Lum vs TNBC	92.25
MPO	MMP7	348	-1.49408437220419	0.016210527050	down	-1.4470721525541	0.04699025864	down	Lum vs TNBC	91.5
PTX3	MMP8	921	-1.18856541021159	0.00615469728	down	-1.7168277925239	0.01189142896	down	Lum vs TNBC	90.75
GATA3	ARMT1	161	1.56099224227678	0.005256005259	up	1.26728421484745	0.01238752624	up	Lum vs TNBC	90.25
CA12	AGR2	262	1.21521589710955	0.025624814650	up	1.64766155608393	0.01544107015	up	Lum vs TNBC	89.25
CRISP3	AGR3	435	-1.14989332884811	0.048077614562	down	1.77364924191116	0.01189142896	up	Lum vs TNBC	88.5
GATA3	CA12	266	1.56099224227678	0.005256005259	up	1.21521589710955	0.02562481465	up	Lum vs TNBC	86.75
IGF2BP3	ELAVL2	655	-1.97911367542364	0.004278877978	down	-1.0388459328925	0.02191670140	down	Lum vs TNBC	86.25
AGR2	ABAT	165	1.64766155608393	0.015441070152	up	1.14368033838458	0.01989277062	up	Lum vs TNBC	86.25
ZSCAN18	SFRP1	409	1.2026942881533	0.002353623305	up	-1.6075966427277	0.00131802560	down	Lum vs TNBC	85.75
PSAT1	PKIB	278	-1.21539270860082	0.036012901814	down	1.56744312338406	0.01238752624	up	Lum vs TNBC	85.5
MLPH	AGR3	239	1.09944013441442	0.020408687162	up	1.77364924191116	0.01189142896	up	Lum vs TNBC	85.5
STUB1	CA9	164	1.06611625214478	0.00779349570	up	-1.9302827885203	0.00779349570	down	Lum vs TNBC	85.5
KRT86	CRISP3	176	-1.59842456182153	0.034306135658	down	-1.1498933288481	0.04807761456	down	Lum vs TNBC	84.75
PROM1	MMP9	526	-1.31132466162209	0.013988179488	down	-1.5532830691434	0.00615469728	down	Lum vs TNBC	83.25
MPO	IGF2BP2	208	-1.49408437220419	0.016210527050	down	-1.3340806034069	0.00195836230	down	Lum vs TNBC	82.75
PBLD	CA9	153	1.03669460114209	0.01935580495	up	-1.9302827885203	0.00779349570	down	Lum vs TNBC	82.75
CRISP3	AGR2	395	-1.14989332884811	0.048077614562	down	1.64766155608393	0.01544107015	up	Lum vs TNBC	81.5
LMNB1	IGF2BP3	156	-1.0266418392669	0.036012901814	down	-1.9791136754236	0.00427887797	down	Lum vs TNBC	80.75
MLPH	AGR2	239	1.09944013441442	0.020408687162	up	1.64766155608393	0.01544107015	up	Lum vs TNBC	78.5
PROM1	MPO	167	-1.31132466162209	0.013988179488	down	-1.4940843722041	0.01621052705	down	Lum vs TNBC	78.5
STARD10	HID1	200	1.15536241737845	0.010609342032	up	1.58508532129184	0.00131802560	up	Lum vs TNBC	78.25
SULT2B1	IGF2BP2	235	1.46812314870425	0.008256715953	up	-1.3340806034069	0.00195836230	down	Lum vs TNBC	77.75
MMP7	KRT18	168	-1.4470721525541	0.046990258645	down	1.33680454995867	0.00427887797	up	Lum vs TNBC	77.5
MMP9	CRISP3	915	-1.55328306914342	0.00615469728	down	-1.1498933288481	0.04807761456	down	Lum vs TNBC	77.25
SFRP1	CELSR1	206	-1.60759664272772	0.00131802560	down	1.10333427109445	0.0213913741	up	Lum vs TNBC	77.25
PLD1	MMP8	157	-1.03486290008617	0.023347093858	down	-1.7168277925239	0.01189142896	down	Lum vs TNBC	74.25
PROM1	MMP7	320	-1.31132466162209	0.013988179488	down	-1.4470721525541	0.04699025864	down	Lum vs TNBC	73.5
PKIB	GAMT	171	1.56744312338406	0.012387526245	up	1.05727260695512	0.01502551370	up	Lum vs TNBC	71.75
MPO	CRISP3	290	-1.49408437220419	0.016210527050	down	-1.1498933288481	0.04807761456	down	Lum vs TNBC	71.25
SHROOM3	MUC16	192	1.03069776557378	0.021724394758	up	-1.6911456895516	0.02942771016	down	Lum vs TNBC	70.5
MMP9	GPT	380	-1.55328306914342	0.00615469728	down	-1.1234839137186	0.0465410600	down	Lum vs TNBC	69.75
GATA3	GAMT	170	1.56099224227678	0.005256005259	up	1.05727260695512	0.01502551370	up	Lum vs TNBC	69.5
NAT1	GSTM3	240	1.53509503707609	0.018436103610	up	1.13326085359944	0.0138818699	up	Lum vs TNBC	69.25
KRT18	IGF2BP2	161	1.33680454995867	0.004278877978	up	-1.3340806034069	0.00195836230	down	Lum vs TNBC	69.25
PROM1	KRT18	543	-1.31132466162209	0.013988179488	down	1.33680454995867	0.00427887797	up	Lum vs TNBC	68.25
PTX3	MPO	474	-1.18856541021159	0.00615469728	down	-1.4940843722041	0.01621052705	down	Lum vs TNBC	68.25
SULT2B1	STARD10	290	1.46812314870425	0.008256715953	up	1.15536241737845	0.01060934203	up	Lum vs TNBC	67.75
S100A9	MMP8	236	-1.01114990788157	0.035199394830	down	-1.7168277925239	0.01189142896	down	Lum vs TNBC	67.75
MPO	GSTM3	271	-1.49408437220419	0.016210527050	down	1.13326085359944	0.0138818699	up	Lum vs TNBC	66.25
MPO	GPT	684	-1.49408437220419	0.016210527050	down	-1.1234839137186	0.0465410600	down	Lum vs TNBC	63.75
PTX3	MMP7	167	-1.18856541021159	0.00615469728	down	-1.4470721525541	0.04699025864	down	Lum vs TNBC	63.25
MMP9	LMNB1	198	-1.55328306914342	0.00615469728	down	-1.0266418392669	0.0360129018	down	Lum vs TNBC	61.25
STUB1	MMP9	187	1.06611625214478	0.00779349570	up	-1.5532830691434	0.00615469728	down	Lum vs TNBC	61.25

LMNB1	GATA3	192	-1.0266418392669	0.036012901814	down	1.56099224227679	0.00525600525	up	Lum vs TNBC	57.5
ZNF385A	PADI2	198	1.0930844430359	0.01282636098	up	-1.4824069802832	0.01398817948	down	Lum vs TNBC	56.75
PLD1	MMP9	236	-1.03486290008617	0.023347093858	down	-1.5532830691434	0.00615469728	down	Lum vs TNBC	56.5
PBLD	MPO	157	1.03669460114209	0.01935580495	up	-1.4940843722041	0.01621052705	down	Lum vs TNBC	53.75
PSAT1	ANLN	200	-1.21539270860082	0.036012901814	down	-1.1972358662187	0.02040868716	down	Lum vs TNBC	51.25
KRT18	GPT	574	1.33680454995867	0.004278877978	up	-1.1234839137186	0.04654106001	down	Lum vs TNBC	50.25
STARD10	ARMT1	197	1.15536241737845	0.010609342032	up	1.26728421484745	0.01238752624	up	Lum vs TNBC	48.25
PLD1	MMP7	158	-1.03486290008617	0.023347093858	down	-1.4470721525541	0.04699025864	down	Lum vs TNBC	46.75
PROM1	GPT	161	-1.31132466162209	0.013988179488	down	-1.1234839137186	0.04654106001	down	Lum vs TNBC	45.75
PSAT1	ABAT	190	-1.21539270860082	0.036012901814	down	1.14368033838458	0.01989277062	up	Lum vs TNBC	45.5
SMOC1	SHROOM3	167	-1.37637457642024	0.024028630379	down	1.03069776557378	0.02172439475	up	Lum vs TNBC	45.5
S100A9	MPO	537	-1.01114990788157	0.035199394830	down	-1.4940843722041	0.01621052705	down	Lum vs TNBC	45.25
SIGIRR	PTX3	570	1.15575005295883	0.00615469728	up	-1.1885654102115	0.00615469728	down	Lum vs TNBC	42.75
IGF2BP2	ELAVL2	282	-1.3340806034069	0.00195836230	down	-1.0388459328925	0.02191670140	down	Lum vs TNBC	42.75
PSAT1	GPT	333	-1.21539270860082	0.036012901814	down	-1.1234839137186	0.04654106001	down	Lum vs TNBC	40.5
S100A9	MMP7	186	-1.01114990788157	0.035199394830	down	-1.4470721525541	0.04699025864	down	Lum vs TNBC	40.25
LMNB1	KRT18	193	-1.0266418392669	0.036012901814	down	1.33680454995867	0.00427887797	up	Lum vs TNBC	37.75
PLD1	FRS2	240	-1.03486290008617	0.023347093858	down	1.26534691423939	0.00689028886	up	Lum vs TNBC	34.25
GPT	ABAT	457	-1.12348391371864	0.046541060010	down	1.14368033838458	0.01989277062	up	Lum vs TNBC	33.5
CMBL	ABAT	359	1.0724889567209	0.011972535429	up	1.14368033838458	0.01989277062	up	Lum vs TNBC	30.5
SHROOM3	ANLN	250	1.03069776557378	0.021724394758	up	-1.1972358662187	0.02040868716	down	Lum vs TNBC	27.75
S100A9	CA12	168	-1.01114990788157	0.035199394830	down	1.21521589710955	0.02562481465	up	Lum vs TNBC	25.25
ZNF385A	GAMT	157	1.0930844430359	0.01282636098	up	1.05727260695512	0.01502551370	up	Lum vs TNBC	21.75
PLD1	GSTM3	238	-1.03486290008617	0.023347093858	down	1.13326085359944	0.01388186991	up	Lum vs TNBC	21.5
GAMT	CMBL	212	1.05727260695512	0.015025513708	up	1.0724889567209	0.01197253542	up	Lum vs TNBC	18.5
STUB1	PLD1	167	1.06611625214478	0.00779349570	up	-1.0348629000861	0.02334709385	down	Lum vs TNBC	16.75
S100A8	CAMP	516	-1.89881380908075	0.016210527050	down	-2.0508096507284	0.01476609731	down	Lum vs TNBC	158.5
S100A8	IGF2BP3	215	-1.89881380908075	0.016210527050	down	-1.9791136754236	0.00427887797	down	Lum vs TNBC	154.5
STUB1	LMNB1	165	1.06611625214478	0.00779349570	up	-1.0266418392669	0.0360129018	down	Lum vs TNBC	15.25
PKIB	FABP7	153	1.56744312338406	0.012387526249	up	-2.0632447723907	0.01621052705	down	Lum vs TNBC	148.75
MUC16	CA9	189	-1.69114568955163	0.02942771016	down	-1.9302827885203	0.00779349570	down	Lum vs TNBC	144.75
S100A8	MMP8	350	-1.89881380908075	0.016210527050	down	-1.7168277925239	0.01189142896	down	Lum vs TNBC	144.25
MMP9	CAMP	937	-1.55328306914342	0.00615469728	down	-2.0508096507284	0.01476609731	down	Lum vs TNBC	139.25
GATA3	CA9	161	1.56099224227678	0.005256005259	up	-1.9302827885203	0.00779349570	down	Lum vs TNBC	135.75
MMP9	IGF2BP3	432	-1.55328306914342	0.00615469728	down	-1.9791136754236	0.00427887797	down	Lum vs TNBC	135.25
MPO	CAMP	670	-1.49408437220419	0.016210527050	down	-2.0508096507284	0.01476609731	down	Lum vs TNBC	133.25
MMP9	CA9	680	-1.55328306914342	0.00615469728	down	-1.9302827885203	0.00779349570	down	Lum vs TNBC	131.5
PADI2	CAMP	329	-1.48240698028328	0.013988179488	down	-2.0508096507284	0.01476609731	down	Lum vs TNBC	130.25
MPO	IGF2BP3	213	-1.49408437220419	0.016210527050	down	-1.9791136754236	0.00427887797	down	Lum vs TNBC	129.25
S100A8	MMP9	468	-1.89881380908075	0.016210527050	down	-1.5532830691434	0.00615469728	down	Lum vs TNBC	126.5
MMP7	CAMP	223	-1.4470721525541	0.046990258649	down	-2.0508096507284	0.01476609731	down	Lum vs TNBC	124.5
S100A8	MPO	468	-1.89881380908075	0.016210527050	down	-1.4940843722041	0.01621052705	down	Lum vs TNBC	121.75
SFRP1	GATA3	225	-1.60759664272772	0.00131802560	down	1.56099224227678	0.00525600525	up	Lum vs TNBC	121.5
MUC16	MMP9	243	-1.69114568955163	0.02942771016	down	-1.5532830691434	0.00615469728	down	Lum vs TNBC	120.5
PROM1	FABP7	451	-1.31132466162209	0.013988179488	down	-2.0632447723907	0.01621052705	down	Lum vs TNBC	118.25
S100A8	MMP7	280	-1.89881380908075	0.016210527050	down	-1.4470721525541	0.04699025864	down	Lum vs TNBC	116.75
MMP7	CA9	255	-1.4470721525541	0.046990258649	down	-1.9302827885203	0.00779349570	down	Lum vs TNBC	116.75
SFRP1	MMP9	250	-1.60759664272772	0.00131802560	down	-1.5532830691434	0.00615469728	down	Lum vs TNBC	116.75
MMP8	MMP7	153	-1.71682779252394	0.011891428964	down	-1.4470721525541	0.04699025864	down	Lum vs TNBC	113.25
PROM1	IGF2BP3	236	-1.31132466162209	0.013988179488	down	-1.9791136754236	0.00427887797	down	Lum vs TNBC	111.25
MUC16	MMP7	311	-1.69114568955163	0.02942771016	down	-1.4470721525541	0.04699025864	down	Lum vs TNBC	110.75
SULT2B1	AGR2	153	1.46812314870425	0.008256715953	up	1.64766155608393	0.01544107015	up	Lum vs TNBC	109.75
PROM1	CA9	398	-1.31132466162209	0.013988179488	down	-1.9302827885203	0.00779349570	down	Lum vs TNBC	107.5
MUC16	KRT18	265	-1.69114568955163	0.02942771016	down	1.33680454995867	0.00427887797	up	Lum vs TNBC	105.5
CA9	CA12	255	-1.93028278852033	0.00779349570	down	1.21521589710955	0.02562481465	up	Lum vs TNBC	103.75
IGF2BP3	ANLN	215	-1.97911367542364	0.004278877978	down	-1.1972358662187	0.02040868716	down	Lum vs TNBC	102.75
SFRP1	KRT18	150	-1.60759664272772	0.00131802560	down	1.33680454995867	0.00427887797	up	Lum vs TNBC	101.75

MPO	MMP9	714	-1.49408437220419	0.016210527050	down	-1.5532830691434	0.006154697288	down	Lum vs TNBC	101.25
KRT18	AGR2	452	1.33680454995867	0.004278877978	up	1.64766155608393	0.015441070155	up	Lum vs TNBC	101.25
SMOC1	SFRP1	196	-1.37637457642024	0.024028630379	down	-1.6075966427277	0.001318025600	down	Lum vs TNBC	101.25
SMOC1	KRT86	350	-1.37637457642024	0.024028630379	down	-1.5984245618215	0.034306135655	down	Lum vs TNBC	100.5
MMP8	CAMP	933	-1.71682779252394	0.011891428964	down	-2.0508096507284	0.01476609731	down	Lum vs TNBC	155
AGR3	AGR2	765	1.77364924191116	0.011891428964	up	1.64766155608393	0.015441070155	up	Lum vs TNBC	138
MUC16	AGR2	248	-1.69114568955163	0.02942771016	down	1.64766155608393	0.015441070155	up	Lum vs TNBC	134
GATA3	AGR3	234	1.56099224227678	0.005256005259	up	1.77364924191116	0.011891428966	up	Lum vs TNBC	132
MMP9	MMP8	964	-1.55328306914342	0.00615469728	down	-1.7168277925239	0.011891428966	down	Lum vs TNBC	125
GATA3	AGR2	251	1.56099224227678	0.005256005259	up	1.64766155608393	0.015441070155	up	Lum vs TNBC	125
MPO	MMP8	626	-1.49408437220419	0.016210527050	down	-1.7168277925239	0.011891428966	down	Lum vs TNBC	119
FRS2	FABP7	400	1.26534691423939	0.006890288862	up	-2.0632447723907	0.016210527055	down	Lum vs TNBC	115
MMP9	GATA3	304	-1.55328306914342	0.00615469728	down	1.56099224227678	0.005256005255	up	Lum vs TNBC	112
KRT18	CA9	293	1.33680454995867	0.004278877978	up	-1.9302827885203	0.007793495700	down	Lum vs TNBC	112
IGF2BP3	IGF2BP2	301	-1.97911367542364	0.004278877978	down	-1.3340806034069	0.001958362300	down	Lum vs TNBC	111
S100A8	IGF2BP2	211	-1.89881380908075	0.016210527050	down	-1.3340806034069	0.001958362300	down	Lum vs TNBC	108
MMP8	KRT18	169	-1.71682779252394	0.011891428964	down	1.33680454995867	0.00427887797	up	Lum vs TNBC	108
SFRP1	MMP7	330	-1.60759664272772	0.00131802560	down	-1.4470721525541	0.046990258644	down	Lum vs TNBC	107
MPO	GATA3	274	-1.49408437220419	0.016210527050	down	1.56099224227678	0.005256005255	up	Lum vs TNBC	106
MMP7	AGR2	230	-1.4470721525541	0.046990258645	down	1.64766155608393	0.015441070155	up	Lum vs TNBC	106
PSAT1	IGF2BP3	215	-1.21539270860082	0.036012901814	down	-1.9791136754236	0.00427887797	down	Lum vs TNBC	106
PTX3	CAMP	923	-1.18856541021159	0.00615469728	down	-2.0508096507284	0.01476609731	down	Lum vs TNBC	105
ZNF703	HID1	298	1.44938753695443	0.004278877978	up	1.58508532129184	0.001318025600	up	Lum vs TNBC	103
ARMT1	AGR3	250	1.26728421484745	0.012387526245	up	1.77364924191116	0.011891428966	up	Lum vs TNBC	101
SULT2B1	GATA3	185	1.46812314870425	0.008256715953	up	1.56099224227678	0.005256005255	up	Lum vs TNBC	101
CRISP3	CAMP	916	-1.14989332884811	0.048077614562	down	-2.0508096507284	0.01476609731	down	Lum vs TNBC	100
ZNF703	GATA3	384	1.44938753695443	0.004278877978	up	1.56099224227678	0.005256005255	up	Lum vs TNBC	99
FABP7	ABAT	171	-2.06324477239071	0.016210527050	down	1.14368033838458	0.019892770622	up	Lum vs TNBC	98
MMP8	CRISP3	938	-1.71682779252394	0.011891428964	down	-1.1498933288481	0.048077614566	down	Lum vs TNBC	93
PROM1	GATA3	210	-1.31132466162209	0.013988179488	down	1.56099224227678	0.005256005255	up	Lum vs TNBC	88
MMP8	GSTM3	156	-1.71682779252394	0.011891428964	down	1.13326085359944	0.01388186991	up	Lum vs TNBC	88
S100A9	CAMP	496	-1.01114990788157	0.035199394830	down	-2.0508096507284	0.01476609731	down	Lum vs TNBC	82
ULBP2	IGF2BP3	410	-1.00310699181023	0.021916701402	down	-1.9791136754236	0.00427887797	down	Lum vs TNBC	76
SULT2B1	PROM1	222	1.46812314870425	0.008256715953	up	-1.3113246616220	0.013988179488	down	Lum vs TNBC	76
PTX3	MMP9	939	-1.18856541021159	0.00615469728	down	-1.5532830691434	0.006154697288	down	Lum vs TNBC	73
S100A9	S100A8	999	-1.01114990788157	0.035199394830	down	-1.8988138090807	0.016210527055	down	Lum vs TNBC	72
GATA3	CELSR1	164	1.56099224227678	0.005256005259	up	1.10333427109445	0.02139137411	up	Lum vs TNBC	72
STUB1	HID1	321	1.06611625214478	0.00779349570	up	1.58508532129184	0.001318025600	up	Lum vs TNBC	70
STUB1	GATA3	240	1.06611625214478	0.00779349570	up	1.56099224227678	0.005256005255	up	Lum vs TNBC	66

SIGIRR	MPO	171	1.15575005295883	0.00615469728	up	-1.4940843722041	0.0162105270	down	Lum vs TNBC	66
KRT18	CA12	338	1.33680454995867	0.00427887797	up	1.2152158971095	0.0256248146	up	Lum vs TNBC	63
PROM1	FRS2	289	-1.31132466162209	0.01398817948	down	1.2653469142393	0.0068902888	up	Lum vs TNBC	61
SEC16A	KRT18	223	1.13623842473654	0.00427887797	up	1.3368045499586	0.0042788779	up	Lum vs TNBC	52
S100A9	MMP9	496	-1.01114990788157	0.03519939483	down	-1.5532830691434	0.0061546972	down	Lum vs TNBC	50
PBLD	DNAJC12	300	1.03669460114209	0.01935580495	up	1.3590582798781	0.0042788779	up	Lum vs TNBC	46
STARD10	PSAT1	157	1.15536241737845	0.01060934203	up	-1.2153927086008	0.0360129018	down	Lum vs TNBC	46
PTX3	CRISP3	903	-1.18856541021159	0.00615469728	down	-1.1498933288481	0.0480776145	down	Lum vs TNBC	43
PSAT1	PBLD	269	-1.21539270860082	0.03601290181	down	1.0366946011420	0.0193558049	up	Lum vs TNBC	34
PSAT1	LMNB1	184	-1.21539270860082	0.03601290181	down	-1.0266418392669	0.0360129018	down	Lum vs TNBC	32
MLPH	GSTM3	315	1.09944013441442	0.02040868716	up	1.1332608535994	0.0138818699	up	Lum vs TNBC	30
SYTL2	MLPH	761	1.12611146885043	0.02473841315	up	1.0994401344144	0.0204086871	up	Lum vs TNBC	26
PTX3	CFHR5	610	-1.18856541021159	0.00615469728	down	-1.0013255787287	0.0477994365	down	Lum vs TNBC	26
LMNB1	ANLN	319	-1.0266418392669	0.03601290181	down	-1.1972358662187	0.0204086871	down	Lum vs TNBC	26
SHROOM3	CELSR1	350	1.03069776557378	0.02172439475	up	1.1033342710944	0.0213913741	up	Lum vs TNBC	15

Her2 vs Luminal

node1	node2	score	logFC.node1	fdr.node1	dir.node1	logFC.node2	fdr.node2	dir.node2	comparison	rank
ERBB2	BCAM	159	2.92106666941437	0.000357457690	up	-1.29359579264557	0.03974505825	down	Her2 vs Lum	99.5
STIM1	SRCIN1	158	-1.3611532710236	0.036066329929	down	1.92302156351116	0.01516544880	up	Her2 vs Lum	99.5
TUBGCP6	MYO18A	207	-1.5050689171198	0.011824613205	down	1.54871371557162	0.04412625575	up	Her2 vs Lum	97.75
MYO18A	CEP97	177	1.54871371557162	0.044126255757	up	-1.52955248402465	0.01523572254	down	Her2 vs Lum	97.75
IDI1	COASY	646	1.6724125944539	0.003910303078	up	-1.47586288399591	0.02568911614	down	Her2 vs Lum	96.5
CSNK1E	BCAS1	203	1.31920050899314	0.040134609003	up	2.35354000342654	0.04975077423	up	Her2 vs Lum	96.5
ZNF24	COIL	167	-1.4407932485367	0.040134609003	down	1.59306519564466	0.04233468622	up	Her2 vs Lum	96.25
TUBGCP6	CEP97	363	-1.5050689171198	0.011824613205	down	-1.52955248402465	0.01523572254	down	Her2 vs Lum	95.25
WWP1	BCAS1	169	-1.3096600090512	0.039745058250	down	2.35354000342654	0.04975077423	up	Her2 vs Lum	94.25
PTGFRN	PRELP	199	-1.3085298649130	0.042962593308	down	-2.01676334108493	0.04233468622	down	Her2 vs Lum	90.5
PLEC	MYO18A	248	-1.4136757101834	0.042334686226	down	1.54871371557162	0.04412625575	up	Her2 vs Lum	89.75
NDC80	ERBB2	189	1.25018093170811	0.049750774235	up	2.92106666941437	0.00035745769	up	Her2 vs Lum	89.25
NCOR2	BCAS1	176	-1.2905116463869	0.042962593308	down	2.35354000342654	0.04975077423	up	Her2 vs Lum	89.25
TUBGCP6	CSNK1E	925	-1.5050689171198	0.011824613205	down	1.31920050899314	0.04013460900	up	Her2 vs Lum	82.5
ZFP91	NCOR2	185	-1.5954304443201	0.011824613205	down	-1.29051164638699	0.04296259330	down	Her2 vs Lum	82.25
ERBB2	CIC	395	2.92106666941437	0.000357457690	up	-1.20165347129357	0.03997117237	down	Her2 vs Lum	81.5
EVL	ATAD2B	165	-1.7533294392377	0.037284905115	down	1.26227649129492	0.01614314166	up	Her2 vs Lum	81.5
FDFT1	ATAD2B	154	1.69835178662458	0.017122040596	up	1.26227649129492	0.01614314166	up	Her2 vs Lum	80.25
PPP1R12C	DNM1L	150	-1.3287042640665	0.007922153838	down	-1.49181449009785	0.04233468622	down	Her2 vs Lum	79.25
CSNK1E	CEP97	911	1.31920050899314	0.040134609003	up	-1.52955248402465	0.01523572254	down	Her2 vs Lum	77.75
NCOR2	COIL	230	-1.2905116463869	0.042962593308	down	1.59306519564466	0.04233468622	up	Her2 vs Lum	76.25
GAPVD1	ERBB2	175	-1.0588495188131	0.041619322188	down	2.92106666941437	0.00035745769	up	Her2 vs Lum	73.75
CEP97	ATAD2B	163	-1.5295524840246	0.015235722547	down	1.26227649129492	0.01614314166	up	Her2 vs Lum	73.75
ZNF24	NCOR2	185	-1.4407932485367	0.040134609003	down	-1.29051164638699	0.04296259330	down	Her2 vs Lum	73.5
HABP4	COIL	347	-1.2811868223981	0.015235722547	down	1.59306519564466	0.04233468622	up	Her2 vs Lum	73.25
WWP1	DNM1L	226	-1.3096600090512	0.039745058250	down	-1.49181449009785	0.04233468622	down	Her2 vs Lum	73.25

SP3	SETD1A	175	-1.9262381507408	0.003950868024	down	-1.19695436451262	0.01523572254	down	Her2 vs Lum	71.75
TUBGCP6	ATAD2B	155	-1.5050689171198	0.011824613205	down	1.26227649129492	0.01614314166	up	Her2 vs Lum	71.75
PPP1R12C	KCMF1	220	-1.3287042640665	0.007922153838	down	-1.39319346444928	0.01523572254	down	Her2 vs Lum	70.75
PLEC	NCOR2	200	-1.4136757101834	0.042334686226	down	-1.29051164638699	0.04296259330	down	Her2 vs Lum	70.25
DNM1L	ATAD2B	230	-1.4918144900978	0.042334686226	down	1.26227649129492	0.01614314166	up	Her2 vs Lum	69.5
TUBGCP6	NDC80	656	-1.5050689171198	0.011824613205	down	1.25018093170811	0.04975077423	up	Her2 vs Lum	67.25
ROR2	CSNK1E	184	-1.3214790868354	0.016555128697	down	1.31920050899314	0.04013460900	up	Her2 vs Lum	66.25
TBCB	MIEN1	174	-1.0152635020120	0.037284905115	down	2.72369274172922	1.68370046045	up	Her2 vs Lum	65.75
COIL	CIC	349	1.59306519564466	0.042334686226	up	-1.20165347129351	0.03997117237	down	Her2 vs Lum	65.5
CDK12	ATAD2B	345	1.41613740097588	0.009103052591	up	1.26227649129492	0.01614314166	up	Her2 vs Lum	65.25
THOC5	COIL	259	1.22903842509459	0.042962593308	up	1.59306519564466	0.04233468622	up	Her2 vs Lum	65.25
PPP1R12C	NCOR2	219	-1.3287042640665	0.007922153838	down	-1.29051164638699	0.04296259330	down	Her2 vs Lum	64.5
TGFB1I1	DNM1L	193	-1.2806773327620	0.017122040596	down	-1.49181449009785	0.04233468622	down	Her2 vs Lum	63.25
NCOR2	CSNK1E	346	-1.2905116463869	0.042962593308	down	1.31920050899314	0.04013460900	up	Her2 vs Lum	57.75
NUF2	NDC80	999	1.38800430624274	0.049750774235	up	1.25018093170811	0.04975077423	up	Her2 vs Lum	56.5
ZCCHC8	COIL	224	-1.1385248920078	0.019152522334	down	1.59306519564466	0.04233468622	up	Her2 vs Lum	56.5
SETD1A	CEP97	171	-1.1969543645126	0.015235722547	down	-1.52955248402465	0.01523572254	down	Her2 vs Lum	56.5
NUF2	NSMCE2	160	1.38800430624274	0.049750774235	up	1.23289758226539	0.04013460900	up	Her2 vs Lum	55.5
ROR2	ATAD2B	154	-1.3214790868354	0.016555128697	down	1.26227649129492	0.01614314166	up	Her2 vs Lum	55.5
ZNF24	SETD1A	153	-1.4407932485367	0.040134609003	down	-1.19695436451262	0.01523572254	down	Her2 vs Lum	55.25
TBCB	ASNS	235	-1.0152635020120	0.037284905115	down	1.65425734469617	0.02194040005	up	Her2 vs Lum	54.75
ZNF24	AP1AR	304	-1.4407932485367	0.040134609003	down	-1.16432055108445	0.00792215383	down	Her2 vs Lum	53.5
VPS13B	MYO18A	183	1.10545186054487	0.010225662626	up	1.54871371557162	0.04412625575	up	Her2 vs Lum	52.25
VAMP3	DNM1L	207	-1.1669088202251	0.025689116144	down	-1.49181449009785	0.04233468622	down	Her2 vs Lum	50.75
SETD1A	CDK12	516	-1.1969543645126	0.015235722547	down	1.41613740097588	0.00910305259	up	Her2 vs Lum	49.25
MYO1B	DNM1L	212	1.13932564792412	0.039745058250	up	-1.49181449009785	0.04233468622	down	Her2 vs Lum	49.25
PPP1R12C	CIC	156	-1.3287042640665	0.007922153838	down	-1.20165347129351	0.03997117237	down	Her2 vs Lum	48.75
ZSCAN18	NCOR2	185	-1.2458428385503	0.049750774235	down	-1.29051164638699	0.04296259330	down	Her2 vs Lum	45.5
ZSCAN18	HABP4	269	-1.2458428385503	0.049750774235	down	-1.28118682239812	0.01523572254	down	Her2 vs Lum	43.25
KCMF1	GAPVD1	207	-1.3931934644492	0.015235722547	down	-1.05884951881310	0.04161932218	down	Her2 vs Lum	43.25
PPP1R12C	ERF	153	-1.3287042640665	0.007922153838	down	-1.06170979676141	0.02613232678	down	Her2 vs Lum	41.25
NCOR2	NCOA4	220	-1.2905116463869	0.042962593308	down	-1.23110306205865	0.04975077423	down	Her2 vs Lum	40.25
WWP1	SETD1A	191	-1.3096600090512	0.039745058250	down	-1.19695436451262	0.01523572254	down	Her2 vs Lum	40.25
CDK13	CDK12	842	-1.0374699914864	0.040134609003	down	1.41613740097588	0.00910305259	up	Her2 vs Lum	39.75
SETD1A	NCOR2	342	-1.1969543645126	0.015235722547	down	-1.29051164638699	0.04296259330	down	Her2 vs Lum	39.5
NCOR2	CIC	419	-1.2905116463869	0.042962593308	down	-1.20165347129351	0.03997117237	down	Her2 vs Lum	37.75
GAPVD1	CSNK1E	654	-1.0588495188131	0.041619322188	down	1.31920050899314	0.04013460900	up	Her2 vs Lum	35.5
TGFB1I1	NCOA4	584	-1.2806773327620	0.017122040596	down	-1.23110306205865	0.04975077423	down	Her2 vs Lum	35.25
PTGFRN	MYO1B	372	-1.3085298649130	0.042962593308	down	1.13932564792412	0.03974505825	up	Her2 vs Lum	35.25
WWP1	GAPVD1	229	-1.3096600090512	0.039745058250	down	-1.05884951881310	0.04161932218	down	Her2 vs Lum	33.25
VPS13B	NCOR2	241	1.10545186054487	0.010225662626	up	-1.29051164638699	0.04296259330	down	Her2 vs Lum	32.75
NCOR2	CRTC2	297	-1.2905116463869	0.042962593308	down	-1.08661338070162	0.02613232678	down	Her2 vs Lum	31.25
NCOR2	ERF	288	-1.2905116463869	0.042962593308	down	-1.06170979676141	0.02613232678	down	Her2 vs Lum	30.25
VAMP3	ATAD2B	165	-1.1669088202251	0.025689116144	down	1.26227649129492	0.01614314166	up	Her2 vs Lum	29.5
NCOR2	GAPVD1	183	-1.2905116463869	0.042962593308	down	-1.05884951881310	0.04161932218	down	Her2 vs Lum	28.25
SETD1A	NSMCE2	275	-1.1969543645126	0.015235722547	down	1.23289758226539	0.04013460900	up	Her2 vs Lum	27.5
TGFB1I1	MYO1B	182	-1.2806773327620	0.017122040596	down	1.13932564792412	0.03974505825	up	Her2 vs Lum	27.25
ZSCAN18	SETD1A	152	-1.2458428385503	0.049750774235	down	-1.19695436451262	0.01523572254	down	Her2 vs Lum	27.25
GAPVD1	ATAD2B	278	-1.0588495188131	0.041619322188	down	1.26227649129492	0.01614314166	up	Her2 vs Lum	24.75
SETD1A	CIC	199	-1.1969543645126	0.015235722547	down	-1.20165347129351	0.03997117237	down	Her2 vs Lum	23.75
CDK13	ATAD2B	327	-1.0374699914864	0.040134609003	down	1.26227649129492	0.01614314166	up	Her2 vs Lum	23.5
NCOA4	AP1AR	238	-1.2311030620586	0.049750774235	down	-1.16432055108445	0.00792215383	down	Her2 vs Lum	23.5
TSTD2	ATAD2B	539	1.03268660527333	0.036335792497	up	1.26227649129492	0.01614314166	up	Her2 vs Lum	22.75
VAMP3	CTNNBIP1	224	-1.1669088202251	0.025689116144	down	-1.21752978631102	0.04233468622	down	Her2 vs Lum	21.75
SETD1A	ERF	347	-1.1969543645126	0.015235722547	down	-1.06170979676141	0.02613232678	down	Her2 vs Lum	16.25
PADI2	ERBB2	451	3.11288295028304	0.003377470650	up	2.92106666941437	0.00035745769	up	Her2 vs Lum	141.75
MIEN1	GRB7	733	2.72369274172922	1.683700460458	up	3.3545727279356	1.68370046045	up	Her2 vs Lum	141.5
MIEN1	ERBB2	635	2.72369274172922	1.683700460458	up	2.92106666941437	0.00035745769	up	Her2 vs Lum	137.25

GRB7	BCAS1	189	3.3545727279356	1.683700460458	up	2.35354000342654	0.04975077423	up	Her2 vs Lum	136.25
SP3	ERBB2	447	-1.9262381507408	0.003950868024	down	2.92106666941437	0.00035745769	up	Her2 vs Lum	132.5
SRCIN1	ERBB2	465	1.92302156351116	0.015165448809	up	2.92106666941437	0.00035745769	up	Her2 vs Lum	131.25
EVL	ERBB2	201	-1.7533294392377	0.037284905115	down	2.92106666941437	0.00035745769	up	Her2 vs Lum	130.5
ERBB2	CCL21	244	2.92106666941437	0.000357457690	up	1.84813241510186	0.0174017415	up	Her2 vs Lum	129.5
TMEM51	PRELP	440	-2.0684505586513	0.001927988161	down	-2.0167633410849	0.04233468622	down	Her2 vs Lum	127.75
ERBB2	ASNS	152	2.92106666941437	0.000357457690	up	1.65425734469617	0.02194040005	up	Her2 vs Lum	123.75
HMGCS1	FDFT1	939	1.96054332885551	0.015235722547	up	1.69835178662458	0.01712204059	up	Her2 vs Lum	121.5
SETD1A	CDK13	495	-1.1969543645126	0.015235722547	down	-1.0374699914864	0.04013460900	down	Her2 vs Lum	12.75
HMGCS1	ASNS	176	1.96054332885551	0.015235722547	up	1.65425734469617	0.02194040005	up	Her2 vs Lum	118.25
PRELP	ECM2	291	-2.0167633410849	0.042334686226	down	-1.5939932482587	0.04975077423	down	Her2 vs Lum	117.75
IDI1	HMGCS1	988	1.6724125944539	0.003910303078	up	1.96054332885551	0.0152357225	up	Her2 vs Lum	117.5
IDI1	EVL	188	1.6724125944539	0.003910303078	up	-1.7533294392377	0.0372849051	down	Her2 vs Lum	115.75
FDFT1	ASNS	210	1.69835178662458	0.017122040596	up	1.65425734469617	0.02194040005	up	Her2 vs Lum	113.25
PLEC	ERBB2	266	-1.4136757101834	0.042334686226	down	2.92106666941437	0.00035745769	up	Her2 vs Lum	112.75
GRB7	CDK12	492	3.3545727279356	1.683700460458	up	1.41613740097588	0.00910305259	up	Her2 vs Lum	110.25
MIEN1	CDK12	510	2.72369274172922	1.683700460458	up	1.41613740097588	0.00910305259	up	Her2 vs Lum	104.5
ZNF24	METTL2B	227	-1.4407932485367	0.040134609003	down	1.70499050669285	0.0256891161	up	Her2 vs Lum	104.25
DNM1L	DNAJC12	159	-1.4918144900978	0.042334686226	down	-1.6686261967259	0.03974505825	down	Her2 vs Lum	104.25
SP3	COASY	158	-1.9262381507408	0.003950868024	down	-1.4758628839959	0.0256891161	down	Her2 vs Lum	102.25
COASY	ASNS	541	-1.4758628839959	0.025689116144	down	1.65425734469617	0.02194040005	up	Her2 vs Lum	101.75
PRELP	PLEC	245	-2.0167633410849	0.042334686226	down	-1.4136757101834	0.04233468622	down	Her2 vs Lum	100.5
VAMP3	GAPVD1	903	-1.1669088202251	0.025689116144	down	-1.0588495188131	0.04161932218	down	Her2 vs Lum	10.75
GRB7	ERBB2	991	3.3545727279356	1.683700460458	up	2.92106666941437	0.00035745769	up	Her2 vs Lum	143
ERBB2	BCAS1	464	2.92106666941437	0.000357457690	up	2.35354000342654	0.04975077423	up	Her2 vs Lum	133
GRB7	EVL	179	3.3545727279356	1.683700460458	up	-1.7533294392377	0.0372849051	down	Her2 vs Lum	132
ERBB2	COIL	185	2.92106666941437	0.000357457690	up	1.59306519564466	0.04233468622	up	Her2 vs Lum	120
IDI1	FDFT1	986	1.6724125944539	0.003910303078	up	1.69835178662458	0.01712204059	up	Her2 vs Lum	114
IDI1	FGD5	259	1.6724125944539	0.003910303078	up	1.67785745418119	0.04233468622	up	Her2 vs Lum	113
ZNF24	MIEN1	248	-1.4407932485367	0.040134609003	down	2.72369274172922	1.6837004604	up	Her2 vs Lum	111
ERBB2	CDK12	278	2.92106666941437	0.000357457690	up	1.41613740097588	0.00910305259	up	Her2 vs Lum	107
PPP1R12C	ERBB2	225	-1.3287042640665	0.007922153838	down	2.92106666941437	0.00035745769	up	Her2 vs Lum	107
HMGCS1	COASY	828	1.96054332885551	0.015235722547	up	-1.4758628839959	0.0256891161	down	Her2 vs Lum	104
WWP1	ERBB2	294	-1.3096600090512	0.039745058250	down	2.92106666941437	0.00035745769	up	Her2 vs Lum	101
FDFT1	COASY	700	1.69835178662458	0.017122040596	up	-1.4758628839959	0.0256891161	down	Her2 vs Lum	99
CSNK1E	CRYM	416	1.31920050899314	0.040134609003	up	2.85561936906157	0.00730469958	up	Her2 vs Lum	99
FGD5	COASY	165	1.67785745418119	0.042334686226	up	-1.4758628839959	0.0256891161	down	Her2 vs Lum	98
NCOR2	ERBB2	293	-1.2905116463869	0.042962593308	down	2.92106666941437	0.00035745769	up	Her2 vs Lum	96
KCMF1	ASNS	240	-1.3931934644492	0.015235722547	down	1.65425734469617	0.02194040005	up	Her2 vs Lum	95
PLEC	COIL	154	-1.4136757101834	0.042334686226	down	1.59306519564466	0.04233468622	up	Her2 vs Lum	93
TGFB1I1	ERBB2	164	-1.2806773327620	0.017122040596	down	2.92106666941437	0.00035745769	up	Her2 vs Lum	91
SP3	NCOR2	226	-1.9262381507408	0.003950868024	down	-1.2905116463869	0.0429625933	down	Her2 vs Lum	90
ZSCAN18	ERBB2	172	-1.2458428385503	0.049750774235	down	2.92106666941437	0.00035745769	up	Her2 vs Lum	88
NCOA4	ERBB2	290	-1.2311030620586	0.049750774235	down	2.92106666941437	0.00035745769	up	Her2 vs Lum	86
TUBGCP6	NUF2	355	-1.5050689171198	0.011824613205	down	1.38800430624274	0.04975077423	up	Her2 vs Lum	84
SETD1A	ERBB2	167	-1.1969543645126	0.015235722547	down	2.92106666941437	0.00035745769	up	Her2 vs Lum	82
TUBGCP6	CCDC12	191	-1.5050689171198	0.011824613205	down	-1.3026089115524	0.03633579249	down	Her2 vs Lum	81
PPP1R12C	PLEC	224	-1.3287042640665	0.007922153838	down	-1.4136757101834	0.04233468622	down	Her2 vs Lum	72
TGFB1I1	MYO18A	193	-1.2806773327620	0.017122040596	down	1.54871371557162	0.04412625575	up	Her2 vs Lum	68
ZFP91	SETD1A	152	-1.5954304443201	0.011824613205	down	-1.1969543645126	0.0152357225	down	Her2 vs Lum	64
THOC5	MYO18A	606	1.22903842509459	0.042962593308	up	1.54871371557162	0.04412625575	up	Her2 vs Lum	62
PPP1R12C	ATAD2B	215	-1.3287042640665	0.007922153838	down	1.26227649129492	0.01614314166	up	Her2 vs Lum	58
TBCB	FDFT1	188	-1.0152635020120	0.037284905115	down	1.69835178662458	0.01712204059	up	Her2 vs Lum	58
ZFP91	GAPVD1	186	-1.5954304443201	0.011824613205	down	-1.0588495188131	0.04161932218	down	Her2 vs Lum	57
TGFB1I1	PLEC	188	-1.2806773327620	0.017122040596	down	-1.4136757101834	0.04233468622	down	Her2 vs Lum	56
MYO1B	MYO18A	326	1.13932564792412	0.039745058250	up	1.54871371557162	0.04412625575	up	Her2 vs Lum	54
WWP1	ATAD2B	240	-1.3096600090512	0.039745058250	down	1.26227649129492	0.01614314166	up	Her2 vs Lum	52
PLEC	MYO1B	158	-1.4136757101834	0.042334686226	down	1.13932564792412	0.03974505825	up	Her2 vs Lum	49

SETD1A	PLEC	215	-1.1969543645126	0.015235722547	down	-1.41367571018343	0.04233468622	down	Her2 vs Lum	47
NCOR2	ATAD2B	167	-1.2905116463869	0.042962593308	down	1.26227649129492	0.01614314166	up	Her2 vs Lum	47
HABP4	ATAD2B	194	-1.2811868223981	0.015235722547	down	1.26227649129492	0.01614314166	up	Her2 vs Lum	44
VPS13B	CSNK1E	524	1.10545186054487	0.010225662626	up	1.31920050899314	0.04013460900	up	Her2 vs Lum	37
SETD1A	ATAD2B	197	-1.1969543645126	0.015235722547	down	1.26227649129492	0.01614314166	up	Her2 vs Lum	33
NSMCE2	NCOA4	151	1.23289758226539	0.040134609003	up	-1.23110306205865	0.04975077423	down	Her2 vs Lum	31

Her2 vs TNBC

node1	node2	score	logFC.node1	fdr.node1	dir.node1	logFC.node2	fdr.node2	dir.node2	comparison	rank
IDI1	HMGCS1	988	1.6635397765768	0.01470633802	up	2.0044853749669	0.0480372894	up	Her2 vs TNBC	8.75
GRB7	CDK12	492	2.7774513452071	0.00163368428	up	1.3747660988497	0.0435251960	up	Her2 vs TNBC	8.75
PIP4K2B	MIEN1	187	1.0685668170218	0.03488910209	up	2.9398201769112	3.8693442981	up	Her2 vs TNBC	8.75
HMGCS1	FDFT1	939	2.0044853749669	0.04803728941	up	1.7501983292950	0.0480372894	up	Her2 vs TNBC	7.75
SRCIN1	PIP4K2B	223	2.5564067741863	0.00333627479	up	1.0685668170218	0.0348891020	up	Her2 vs TNBC	5.25
ERBB2	BCAS1	464	3.1573192314075	0.00058981874	up	3.4833127054331	0.0147063380	up	Her2 vs TNBC	19.25
MIEN1	ERBB2	635	2.9398201769112	3.86934429811	up	3.1573192314075	0.0005898187	up	Her2 vs TNBC	16.25
GRB7	BCAS1	189	2.7774513452071	0.00163368428	up	3.4833127054331	0.0147063380	up	Her2 vs TNBC	16.25
GRB7	ERBB2	991	2.7774513452071	0.00163368428	up	3.1573192314075	0.0005898187	up	Her2 vs TNBC	14.75
MIEN1	GRB7	733	2.9398201769112	3.86934429811	up	2.7774513452071	0.0016336842	up	Her2 vs TNBC	14.25
ERBB2	CADPS2	193	3.1573192314075	0.00058981874	up	1.9984952133714	0.0329099258	up	Her2 vs TNBC	14.25
ERBB2	CDK12	278	3.1573192314075	0.00058981874	up	1.3747660988497	0.0435251960	up	Her2 vs TNBC	11.75
MIEN1	CDK12	510	2.9398201769112	3.86934429811	up	1.3747660988497	0.0435251960	up	Her2 vs TNBC	10.25
CRYM	CADPS2	283	2.7466388713562	0.03488910209	up	1.9984952133714	0.0329099258	up	Her2 vs TNBC	10.25
VPS13B	IDE	219	1.0492897259217	0.04803728941	up	1.0996353807747	0.0480372894	up	Her2 vs TNBC	1.5
SRCIN1	ERBB2	465	2.5564067741863	0.00333627479	up	3.1573192314075	0.0005898187	up	Her2 vs TNBC	13
PIP4K2B	ERBB2	174	1.0685668170218	0.03488910209	up	3.1573192314075	0.0005898187	up	Her2 vs TNBC	10
PIP4K2B	GRB7	250	1.0685668170218	0.03488910209	up	2.7774513452071	0.0016336842	up	Her2 vs TNBC	8
IDI1	FDFT1	986	1.6635397765768	0.01470633802	up	1.7501983292950	0.0480372894	up	Her2 vs TNBC	7
PIP4K2B	CDK12	245	1.0685668170218	0.03488910209	up	1.3747660988497	0.0435251960	up	Her2 vs TNBC	4
