# Supplementary material for: High‐throughput proteomics of breast cancer interstitial fluid: identification of tumor subtype‐specific serologically relevant biomarkers
Source: Mol Oncol. 2021 Jan 4;15(2):429–61. doi: 10.1002/1878-0261.12850 (PMC7858121; doi:10.1002/1878-0261.12850)
Supplement: Supplementary file 13 — Table S11. A panel of 10 proteins identified in TIF samples in the present study are segregated according to immunohistochemistry (IHC) scores from paired tumor tissues and BC subtype (Her2, luminal, TNBC). [file MOL2-15-429-s013.pdf]

Supplementary Table S11: *A panel of 10 proteins identified in TIF samples in the present study are segregated according toimmunohistochemistry (IHC) scores from paired tumor tissues and BC subtype (Her2, luminal, TNBC).* The last column shows the p-values obtained from Fisher's exact test when the distribution of IHC scores of luminal versus TNBC samples were compared. Please, note that Her2 was not included in Fisher's exact test due to the low number of samples. * Denotes a significant p-value, no adjustment for multiple testing was needed as only 10 tests were performed at alpha-level 5%.

Variable	IHC Score	Her2	Her2 %	Luminal	Luminal %	TNBC	TNBC %	P-values Luminal vs TNBC
**AGR3**	+1	0	0.0	1	5.3	10	83.3	
	+2	3	100.0	8	42.1	2	16.7	
	+3	0	0.0	10	52.6	0	0.0	
	all	3	100.0	19	100.0	12	100.0	< 2.2e-16*
**BCAM**	+1	2	66.6	4	21.1	11	91.7	
	+2	1	33.3	6	31.6	1	8.3	
	+3	0	0.0	9	47.4	0	0.0	
	all	3	100.0	19	100.0	12	100.0	2e-04*
**CELSR1**	+1	3	100.0	4	21.1	8	66.7	
	+2	0	0.0	10	52.6	3	25.0	
	+3	0	0.0	5	26.3	1	8.3	
	all	3	100.0	19	100.0	12	100.0	0.048*
**MIEN1**	+1	0	0.0	1	5.3	3	25.0	
	+2	0	0.0	13	68.4	8	66.7	
	+3	3	100.0	5	26.3	1	8.3	
	all	3	100.0	19	100.0	12	100.0	0.17
**NAT1**	+1	2	66.6	1	5.3	8	66.7	
	+2	1	33.3	9	47.4	4	33.3	
	+3	0	0.0	9	47.4	0	0.0	
	all	3	100.0	19	100.0	12	100.0	< 2.2e-16*
**PIP4K2B**	+1	0	0.0	0	0.0	5	41.7	
	+2	0	0.0	16	84.2	7	58.3	
	+3	3	100.0	3	15.8	0	0.0	
	all	3	100.0	19	100.0	12	100.0	0.0025*
**SEC23B**	+1	0	0.0	1	5.3	5	41.7	
	+2	0	0.0	12	63.2	7	58.3	
	+3	3	100.0	6	31.6	0	0.0	
	all	3	100.0	19	100.0	12	100.0	0.0078*
**THTPA**	+1	3	100.0	2	10.5	12	100.0	
	+2	0	0.0	12	63.2	0	0.0	
	+3	0	0.0	5	26.3	0	0.0	
	all	3	100.0	19	100.0	12	100.0	< 2.2e-16*
**TMEM51**	+1	3	100.0	3	15.8	4	33.3	
	+2	0	0.0	10	52.6	5	41.7	
	+3	0	0.0	6	31.6	3	25.0	
	all	3	100.0	19	100.0	12	100.0	0.63
**ULBP2**	+1	1	33.3	7	36.8	1	8.3	
	+2	1	33.3	11	57.9	3	25.0	
	+3	1	33.3	1	5.3	8	66.7	
	all	3	100.0	19	100.0	12	100.0	0.0015*
