# Supplementary material for: High‐throughput proteomics of breast cancer interstitial fluid: identification of tumor subtype‐specific serologically relevant biomarkers
Source: Mol Oncol. 2021 Jan 4;15(2):429–61. doi: 10.1002/1878-0261.12850 (PMC7858121; doi:10.1002/1878-0261.12850)
Supplement: Supplementary file 14 — Table S12. Test‐statistics and p‐values from ordinal logistic regression with ten protein candidates. [file MOL2-15-429-s014.pdf]

Supplementary Table S12. Table with test-statistics and p-values from ordinal logistic regression with ten protein candidates. Regression analysis was performed using normalized protein abundances from tumor interstitial fluids (TIF) as the predictor (continuous) and immunohistochemistry (IHC) scores from tumor tissue as outcome (discrete, 0, 1, 2 and 3). Stars indicate significant p-values.

	IHC Group	Value	Std.Error	t.value	p.value
**MIEN1**	*Overall*	3.6854	1.17	3.16	1.6e-03*
	0/1	-14.5945	53.69	-0.27	7.9e-01
	1/2	-3.3555	1.04	-3.22	1.3e-03*
	2/3	2.1113	0.93	2.26	2.4e-02*
**PIP4K2B**	*Overall*	7.2733	2.16	3.37	7.6e-04*
	0/1	-12.8887	30.32	-0.43	6.7e-01
	1/2	-1.1632	0.54	-2.14	3.2e-02*
	2/3	2.7723	0.78	3.57	3.6e-04*
**SEC23B**	*Overall*	4.1978	1.21	3.46	5.3e-04*
	0/1	-12.2727	49.24	-0.25	8.0e-01
	1/2	-0.6963	0.45	-1.55	1.2e-01
	2/3	2.7032	0.70	3.86	1.2e-04*
**AGR3**	*Overall*	2.5518	0.59	4.33	1.5e-05*
	0/1	-4.4695	1.02	-4.37	1.3e-05*
	1/2	-1.1839	0.71	-1.66	9.7e-02
	2/3	1.4000	0.66	2.11	3.5e-02*
**BCAM**	*Overall*	2.4641	0.68	3.64	2.7e-04*
	0/1	-1.9597	0.60	-3.29	1.0e-03*
	1/2	0.0048	0.46	0.01	9.9e-01
	2/3	1.3309	0.50	2.69	7.2e-03*
**CELSR1**	*Overall*	1.9959	0.62	3.22	1.3e-03*
	0/1	-1.9942	0.59	-3.40	6.8e-04*
	1/2	-0.3831	0.50	-0.77	4.4e-01
	2/3	4.8710	1.34	3.65	2.7e-04*

**THTPA**	*Overall*	9.6605	2.16	4.47	7.9e-06*
	0/1	-1.6373	0.69	-2.39	1.7e-02*
	1/2	1.5103	0.65	2.34	1.9e-02*
	2/3	5.7611	1.32	4.36	1.3e-05*
**NAT1**	*Overall*	4.6384	1.27	3.66	2.5e-04*
	0/1	-6.9991	1.85	-3.79	1.5e-04*
	1/2	-2.9050	1.24	-2.35	1.9e-02*
	2/3	1.7937	1.01	1.78	7.5e-02
**TMEM51**	*Overall*	6.5402	2.03	3.23	1.2e-03*
	0/1	-28.1039	48.14	-0.58	5.6e-01
	1/2	-0.6843	0.51	-1.35	1.8e-01
	2/3	4.1506	1.20	3.47	5.3e-04*
**ULBP2**	*Overall*	4.2175	1.14	3.70	2.2e-04*
	0/1	-13.3352	44.52	-0.30	7.6e-01
	1/2	-1.3229	0.56	-2.36	1.8e-02*
	2/3	2.5832	0.81	3.20	1.4e-03*
