# Supplementary material for: High‐throughput proteomics of breast cancer interstitial fluid: identification of tumor subtype‐specific serologically relevant biomarkers
Source: Mol Oncol. 2021 Jan 4;15(2):429–61. doi: 10.1002/1878-0261.12850 (PMC7858121; doi:10.1002/1878-0261.12850)
Supplement: Supplementary file 15 — Table S13. Expression profiles for 10 selected proteins in 33 normal human tissues (MNO661; Pantomics, USA) according to immunohistochemistry (IHC) scores. [file MOL2-15-429-s015.pdf]

Supplementary Table S13: *Expression profiles for 10 selected proteins in 33 normal human tissues (MNO661; Pantomics, USA) according to immunohistochemistry (IHC) scores.* Scoring was determined as described in Supplementary Table 1.

**Anatomic site**	**Histology**	**AGR3**	**BCAM**	**CELSR1**	**MIEN1**	**NAT1**	**PIP4K2B**	**SEC23B**	**THTPA**	**TMEM51**	**ULBP2**
**Adrenal gland**	Hyperplasia	0+	1+	0+	1+	1+	1+	1+	0+	1+	1+
**Bladder**	Normal	**2+**	**2+**	0+	1+	**2+**	1+	1+	1+	**2+**	0+
**Bone marrow**	Normal	0+	0+	0+	0+	0+	1+	nt	0+	0+	0+
**Eye**	Normal	0+	1+	1+	1+	1+	1+	**2+**	0+	0+	0+
**Breast**	Normal	1+	1+	1+	1+	0+	0+	1+	0+	0+	1+
**Cerebellum**	Normal	0+	1+	0+	0+	0+	1+	0+	0+	1+	0+
**Cerebral cortex**	Normal	0+	1+	0+	0+	0+	1+	0+	0+	0+	0+
**Fallopian tube**	Normal	**2+**	**2+**	0+	1+	1+	1+	1+	1+	1+	1+
**GI-Esophagus**	Normal	0+	**2+**	0+	1+	0+	1+	1+	1+	1+	1+
**GI-Stomach**	Normal	**2+**	0+	0+	1+	1+	1+	1+	0+	1+	0+
**GI-Small intestine**	Normal	**2+**	1+	1+	1+	1+	1+	1+	0+	1+	0+
**GI-Colon**	Normal	**2+**	1+	0+	1+	0+	1+	0-1+	0+	1+	0+
**GI-Rectum**	Normal	**2+**	1+	0+	1+	0+	1+	0+	0+	1+	0+
**Heart**	Normal	0+	**2+**	0+	1+	0+	0+	1+	0+	0+	**2+**
**Kidney**	Normal	1+	**2+**	0+	1+	1+	1+	0+	0+	**2+**	1+
**Liver**	Normal	1+	1+	0+	1+	1+	0+	1+	0+	0+	1+
**Lung**	Normal	0+	**2+**	0+	1+	0+	0+	1+	0+	0+	0+
**Ovary**	Normal	0+	1+	0+	0+	0+	0+	0+	0+	0+	0+
**Pancreas**	Normal	1+	1+	0+	0+	1+	1+	1+	1+	**2+**	0+
**Parathyroid**	Adenoma	0+	**2+**	0+	1+	0+	1+	1+	0+	1+	1+
**Pituitary gland**	Normal	0+	**2+**	0+	1+	0+	1+	1+	0+	0+	0+
**Placenta**	Normal	0+	1+	0+	1+	1+	1+	0+	0+	0+	0+
**Prostate**	Normal	0+	**2+**	0+	1+	0+	0+	1+	0+	1+	0+
**Skin**	Normal	0+	1+	0+	1+	0+	0+	0+	1+	1+	1+
**Spinal cord**	Normal	0+	1+	0+	1+	0+	0+	1+	0+	0+	0+
**Spleen**	Normal	0+	1+	0+	1+	0+	0+	0+	0+	0+	0+
**Skeletal muscle**	Normal	0+	0+	0+	1+	0+	0+	1+	0+	0+	0+
**Testis**	Normal	1+	1+	0+	1+	1+	0+	1+	0+	0+	0+
**Thymus**	Normal	1+	1+	0+	0+	0+	1+	0+	0+	0+	0+
**Thyroid**	Normal	1+	**2+**	0+	1+	0+	1+	1+	1+	1+	1+
**Tonsil**	Normal	1+	1+	0+	1+	1+	1+	1+	0+	1+	1+
**Uterus-cervix**	Normal	0+	1+	0+	1+	0+	1+	0+	0+	1+	0+
**Uterus-endometrium**	Normal	1+	0+	0+	1+	1+	1+	0+	0+	0+	1+
