# Supplementary material for: High‐throughput proteomics of breast cancer interstitial fluid: identification of tumor subtype‐specific serologically relevant biomarkers
Source: Mol Oncol. 2021 Jan 4;15(2):429–61. doi: 10.1002/1878-0261.12850 (PMC7858121; doi:10.1002/1878-0261.12850)
Supplement: Supplementary file 16 — Supplementary Material [file MOL2-15-429-s016.docx]

**SUPPLEMENTARY INFORMATION:
FIGURES:**

**Supplementary Figure S1:** *Figure showing the effects of batch correction on sample clustering and variance of protein abundance*.

The top panel muntidimensional scaling (MDS) plots highlight how batch correction affects the clustering of samples from different BC subtypes. A: before batch correction, and B: after batch correction (essentially Figure 3). The lower panel of the plot (C) are side-by-side boxplots (before and after batch correction) of 12 randomly selected proteins. The boxplots show how a correction for batch makes the variance of protein abundances more comparable across groups.

**Supplementary Figure S2:**  *Six set-wise heatmaps (A-F) with differentially abundant proteins from DAA comparisons*. The color bar at the top of each heatmap denotes the clinicopathological group of interest. A = Subtypes (luminal, Her2, TNBC), B: Estrogen receptor status (ER+, ER-), C: Her2 receptor status (0, +1, +2, +3), D: Progesterone receptor status (PgR+, PgR-), E: Degree of tumor-infiltrating lymphocytes (0, +1, +2, +3) and F: Tumor grade (1, 2, 3).

**TABLES:**

**Supplementary Table S1.** *List of the antibodies used in this study.*

**Supplementary Table S2.** *Clinicopathological* *characteristics* *of the breast cancer TIF samples which were examined in this study.*

**Supplementary Table S3.** *Proteins returned from LASSO regression and Random Forest models.*

A table of proteins returned from LASSO regression and Random Forest models with BC subtypes (luminal, Her2 and TNBC), estrogen receptor (ER), progesterone receptor (PgR), Her2 receptor (Her2), tumor-infiltrating lymphocytes (TILs), and tumor grade. The table contains Uniprot accession, gene name and weight. Proteins are ordered set wise according to weight. In addition, the table contains out-of-bag (OOB) errors, cross-validation errors (CVs), class errors, and accuracies with confidence intervals from LASSO and Random Forest models.

**Supplementary Table S4:** *An alphabetical list of proteins and their detection profile in tumor, normal, and fat interstitial fluid samples (TIF, NIF, and FIF, respectively).*

[.] denotes that a protein was not in a given set. Only proteins identified in more than one set are included in this table.

**Supplementary Table S5.** *Table with fractions (percentages) of BC samples within clusters belonging to each clinicopathological subgroup.*

This table contains information about the fraction (percentage) of samples belonging to each clinicopathological subgroup within the two clusters (Cluster 1 and Cluster 2) observed in Figure 4. The table has two subtables, one containing percentages within clusters and one with percentages across clusters.

**Supplementary Table S6.** *An* *alphabetical list of 174 (176) differentially abundant TIF proteins according to limma analysis.*

Increases or decreases in expression are presented according to BC subtype, hormone receptor status, and TIL level. It is also indicated whether each protein is detected (Y) or absent (.) in BC cell lines [42], in the human plasma proteome, in TIF samples from another cohort of BC patients [31], and/or is externalized via exosomes [103] or plasma microvesicles [57]. Criterion for significance was an adjusted p.value (FDR) < 0.05 and a log2 fold change of +1 (up-regulated) and -1 (down-regulated). Two of the genes, POSTN and SYTL2, have two different UniProt IDs assigned.

**Supplementary Table S7.** *The results of the differential abundance analysis with limma*.

There are eight subtables from contrasts with BC subtypes (pairwise), estrogen receptor, progesterone receptor, Her2 receptor, degree of tumor-infiltrating lymphocytes, and tumor grade. Each table contains protein ID, gene name, test-statistics, p-values, and log fold changes (logFCs). All proteins in this table were significantly DA after correction for multiple testing and filtering on logFC.

**Supplementary Table S8:** *Results from differential abundance analysis, LASSO regression, and random forest.*

Only proteins identified by at least two out of the three methods are included. Fourteen proteins are listed in bold font, and those with an asterisk were selected for further analysis. Arrows denote abundance directionality (up or down) in TIF samples for comparison. Arrows in parentheses are borderline significant (i.e., corrected p-value < 0.05, yet log-fold change was just shy of either 1 or -1).

**Supplementary Table S9.** *Comparison of the expression profiles for eight of the TIF proteins identified in the present study with the PAM50 prognostic signature* [2,96].

Entries in the table are sorted by protein set, and within these sets, proteins are sorted alphabetically.

**Supplementary Table S10***. Protein-protein interaction networks from analysis using the STRING database*.

Networks included in the table are from sets of proteins differentially abundant between BC subtypes (luminal, Her2 and TNBC). Each table contains protein node pairs, logFCs, adjusted p-values, STRING score (support for interaction), and rank (based on sum of absolute logFC of both node pair).

**Supplementary Table S11:** *A panel of* *10 proteins identified in TIF samples in the present study are segregated according to immunohistochemistry (IHC) scores from paired tumor tissues and BC subtype (Her2, luminal, TNBC).*

The last column shows the p-values obtained from Fisher's exact test when the distribution of IHC scores of luminal versus TNBC samples were compared. Please, note that Her2 was not included in Fisher’s exact test due to the low number of samples. * Denotes a significant p-value, no adjustment for multiple testing was needed as only 10 tests were performed at alpha-level 5%.

**Supplementary Table S12**. *Test-statistics and p-values from ordinal logistic regression with ten protein candidates*. Regression analysis was performed using normalized protein abundances from tumor interstitial fluids (TIF) as the predictor (continuous) and immunohistochemistry (IHC) scores from tumor tissue as the outcome (discrete, 0, 1, 2 and 3). Stars indicate significant p-values.

**Supplementary Table S13:** *Expression profiles for 10 selected proteins in 33 normal human tissues (MNO661; Pantomics, USA) according to immunohistochemistry (IHC) scores.*

Scoring was determined as described in Supplementary Table 1.
